# Immunoregulatory Effects of Myeloid-Derived Suppressor Cell Exosomes in Mouse Model of Autoimmune Alopecia Areata

**DOI:** 10.3389/fimmu.2018.01279

**Published:** 2018-06-06

**Authors:** Margot Zöller, Kun Zhao, N. Natali Kutlu, Nathalie Bauer, Jan Provaznik, Thilo Hackert, Martina Schnölzer

**Affiliations:** ^1^Tumor Cell Biology, Department of Surgery, University Hospital of Heidelberg, Heidelberg, Germany; ^2^Gene Core Unit, EMBL Heidelberg, Heidelberg, Germany; ^3^Pancreas Section, Department of Surgery, University Hospital of Heidelberg, Heidelberg, Germany; ^4^Functional Proteome Analysis, German Cancer Research Center, Heidelberg, Germany

**Keywords:** myeloid-derived suppressor cells, exosomes, alopecia areata, therapy, regulatory T cells

## Abstract

The treatment of autoimmune diseases still poses a major challenge, frequently relying on non-specific immunosuppressive drugs. Current efforts aim at reestablishing self tolerance using immune cells with suppressive activity like the regulatory T cells (Treg) or the myeloid-derived suppressor cells (MDSC). We have demonstrated therapeutic efficacy of MDSC in mouse Alopecia Areata (AA). In the same AA model, we now asked whether MDSC exosomes (MDSC-Exo) can replace MDSC. MDSC-Exo from bone marrow cells (BMC) cultures of healthy donors could substantially facilitate treatment. With knowledge on MDSC-Exo being limited, their suitability needs to be verified in advance. Protein marker profiles suggest comparability of BMC- to *ex vivo* collected inflammatory MDSC/MDSC-Exo in mice with a chronic contact dermatitis, which is a therapeutic option in AA. Proteome analyses substantiated a large overlap of function-relevant molecules in MDSC and MDSC-Exo. Furthermore, MDSC-Exo are taken up by T cells, macrophages, NK, and most avidly by Treg and MDSC-Exo uptake exceeds binding of MDSC themselves. In AA mice, MDSC-Exo preferentially target skin-draining lymph nodes and cells in the vicinity of remnant hair follicles. MDSC-Exo uptake is accompanied by a strong increase in Treg, reduced T helper proliferation, mitigated cytotoxic activity, and a slight increase in lymphocyte apoptosis. Repeated MDSC-Exo application in florid AA prevented progression and sufficed for partial hair regrowth. Deep sequencing of lymphocyte mRNA from these mice revealed a significant increase in immunoregulatory mRNA, including FoxP3 and arginase 1. Downregulated mRNA was preferentially engaged in prohibiting T cell hyperreactivity. Taken together, proteome analysis provided important insights into potential MDSC-Exo activities, these Exo preferentially homing into AA-affected organs. Most importantly, changes in leukocyte mRNA seen after treatment of AA mice with MDSC-Exo sustainably supports the strong impact on the adaptive and the non-adaptive immune system, with Treg expansion being a dominant feature. Thus, MDSC-Exo could potentially serve as therapeutic agents in treating AA and other autoimmune diseases.

## Introduction

Autoimmune disease incidence is steadily increasing (1). Upon progression, autoimmune diseases severely affect the quality of life and can become life threatening (2). Corticosteroid therapy, prevalently used in progressive disease stages (3), is burdened by severe side effects including dampening immune responses against bacteria and viruses (4). This boosted the search for new therapeutic concepts that focus on correcting the breakdown of tolerance by a deficit in regulatory T cells (Treg) (5). One option is myeloid-derived suppressor cells (MDSC) which are supposed to stimulate Treg expansion and activation (6, 7).

Myeloid-derived suppressor cells were originally described to be a hindrance in chronic infections by promoting ζ-chain downregulation (8). Recently, they received much attention for a crucial role in tumor progression, which includes their power in immune response suppression (9, 10). On the other hand, the immunosuppressive features of MDSC were noted to support allogeneic bone marrow transplantation (11). Finally, in several autoimmune diseases, a paucity of MDSC assists disease progression (7).

Myeloid-derived suppressor cells are a heterogeneous group of cells, characterized by myeloid origin, immature state, and in humans mostly by functional activity (12, 13). In mice, MDSC are defined as CD11b+Gr1+. Subgroups are differentiated on the basis of Ly6Chigh (M<macrophage>-MDSC) or Ly6Ghigh (G<granulocyte>-MDSC), M-MDSC exerting stronger suppressive activity (12). The major effector molecules of MDSC are arginase 1 which contributes to ζ-chain downregulation, and iNOS, which induces NO. NO and ROS inhibit T cell proliferation and induce apoptosis. HO-1 inhibits T cell proliferation *via* CO production. IL10 promotes TH2 deviation and type 2 macrophage (Mϕ) polarization. Membrane-bound TGFβ1 supports NK cell anergy and induces Treg. Finally, ADAM17 leads to CD62L cleavage which inhibits T cell homing (13–15).

Recently, it has been accepted that intercellular communication does not essentially depend on cell–cell contact or soluble mediators, and can be efficiently mediated by exosomes (Exo) (16, 17).

Exosomes are small 30–100 nm vesicles (18), which derive from the fusion of multivesicular bodies (MVB) with the plasma membrane (19, 20). Exo are released by many cells and can distribute throughout the body (21). They are composed of a lipid bilayer and contain selected membrane and cytosolic proteins, and coding and non-coding RNA and DNA (22–26). Besides a common set of membrane and cytosolic proteins, which are related to Exo biogenesis (17, 27), Exo also contain cell-type specific proteins (27, 28). There is a difference in the relative abundance of proteins, mRNAs, and miRNAs in Exo and donor cells that implies active sorting into MVB (25, 26, 28). Exo bind to and are taken-up by selective target cells, which can severely alter the fate of these cells (29–33), supporting use of Exo as a therapeutic which were first applied in immunotherapy, where dendritic cells (DC)-derived Exo are equipped for T cell activation and can replace DC (34). Recent comprehensive studies on “inflammatory” MDSC Exo isolated from tumor tissue demonstrated subtle differences to MDSC and suggested functional relevance of some of these Exo components (35, 36). Finally, there is one report of MDSC Exo attenuating DSS-induced colitis in mice (37).

Building on good response rates seen upon using MDSC in treating autoimmune diseases, such as myasthenia gravis, arthritis, inflammatory bowel disease, etc (7, 38–43)., we characterized MDSC Exo that were generated *ex vivo* and *in vitro* to get a hint toward their mode of action. To control the *in vivo* activity, including therapeutic efficacy, a mouse Alopecia areata (AA) model that closely resembles human AA was chosen (44).

Alopecia areata is a T cell-mediated autoimmune disease of the skin with a non-scarring hair loss due to destruction of anagen stage hair follicles (45–47). In humans as well as mice (44, 47) AA is characterized by a perifollicular infiltration of CD4+ and CD8+ T cells and aberrant MHC-I and MHC-II expression on hair follicle epithelium (48). Hair follicle destruction is mediated by CD8+ T cells, transfer studies supporting a specific contribution of both CD8+ and CD4+ T cells (49). AA induction also relies on expansion of TH17, which abundantly secrete TGFβ, IL6, and IL1β (50). TH17 inversely correlate with CD4+CD25+FoxP3+ Treg (51), which inhibit contact-dependent T cell proliferation, induce anergy and IL10 secretion in helper T cells (TH) creating a milieu of “infectious tolerance” (52, 53). Notably, the transfer of Treg can prevent AA induction (49). AA is efficiently treated by induction of a chronic contact eczema by squaric acid dibutylester (SADBE) with a success rate of 50–70% in patients with severe AA and close to 100% in C3H/HeJ mice developing AA spontaneously or after AA-affected skin transplantation (54–56). Important for selecting AA as model, the therapeutic effect of SADBE treatment relies on MDSC expansion and activation (57, 58), which was an important consideration for selecting AA as the model. Notably, SADBE can be replaced by MDSC (59, 60).

We here established the suitability of bone marrow cells (BMC) culture-derived MDSC-Exo with regards to composition and *in vitro* activity. We controlled in the AA model for therapeutic efficacy and elaborated the impact of intravenously injected MDSC-Exo on *in vivo* immune response regulation including Treg.

## Materials and Methods

### Mice

Female C3H/HeJ mice were purchased from The Jackson Laboratory, Bar Harbor, ME, USA.

### Leukocyte Preparation and Separation

Mice were sacrificed by cervical dislocation or were anesthetized in CO_2_ for collecting heparinized peripheral blood by heart puncture. Dorsal skin samples were embedded in OCT compound (Tissue Tek, Sakura, Zoeterwoude, Netherlands) and snap frozen in liquid nitrogen. For the isolation of skin-infiltrating leukocytes (SkIL), skin was layered epidermis uppermost on sterile gauze and incubated 3 × 30 min with a 1 mg/ml trypsin/EDTA solution collecting the isolated cells in RPMI/10% FCS after each incubation. After the final trypsin treatment, pooled cells were washed and incubated for 2 h at 37°C in RPMI/10% FCS/10^−3^M HEPES to allow for surface molecule recovery. Single cell suspensions of skin-draining lymph nodes and spleen [lymph node cells (LNC), SC] were prepared by pressing through fine gauze. BMC were collected by flushing femora and tibiae with PBS. Peritoneal exudate cells were obtained by flushing the peritoneal cavity with PBS/heparin. Peripheral blood leukocytes (PBL) were collected after Ficoll–Hypaque centrifugation. Treg, CD4+, CD8+, NK, and CD11b+Gr1+MDSC cells were enriched by magnetic bead isolation according to the manufacturer’s protocol (Miltenyi, Bergisch Gladbach, Germany). Flow-cytometry revealed an >95% purity of magnetic bead-enriched subpopulations. Viability (trypan blue exclusion) of SkIL ranged between 70 and 85% and of BMC, LNC, SC, and subpopulations between 95 and 98%.

### Antibodies and Reagents

Please see Table S1 in Supplementary Material.

### *In Vitro* MDSC and DC Preparation

Dendritic cells and MDSC were generated from BMC. For DC induction, BMC (2 × 10^6^) were cultured in 10 cm diameter petri dishes in 10 ml RPMI 1640/10% FCS, supplemented with recombinant mouse GMCSF (10 ng/ml) and IL4 (2 ng/ml). Additional 10 ml medium was added after 3 days, and half of the medium was exchanged after 6 days. Loosely adherent cells were harvested at 8 days and seeded in new petri dishes in 10 ml medium adding 0.25 µg/ml LPS for 24 h to induce DC maturation. Matured DC, harvested after 9 days, were washed and suspended in serum-free RPMI containing 10 µg/ml synthetic 9mer H-2K^k^ and H-2Ea^k^ binding peptides of Ha1 and K6irs (Table S2 in Supplementary Material). DC loading with peptides was terminated after 2h-3h (61). MDSC were generated from BMC by culturing in RPMI 1640/10% FCS supplemented with 20 ng/ml GMCSF, 5 ng/ml IL6, and 1 nM PGE2 for 5 days. Where indicated, MDSC were carboxyfluorescein-succinimidylester (CFSE) labeled (5 μM/10^6^ cells).

### Exo Preparation

Exosomes were collected from *in vitro* generated MDSC and from *ex vivo* isolated CD11b+Gr1+SC and BMC by culturing cells for 48 h in the presence of RPMI 1640 with 3% Exo-depleted FCS. Cleared supernatants (2 × 10 min, 500 *g*, 1 × 20 min, 2,000 *g*, 1 × 30 min, 10,000 *g*) were centrifuged (120 min, 100,000 *g*), the pellet was washed (PBS, 2 h, 100,000 *g*), suspended in 40% sucrose overlaid by a discontinuous sucrose gradient (30–5%) and centrifuged (16 h, 100,000 *g*). Exo were collected from the 10–5% sucrose interface. The protein concentration was determined by Bradford. Where indicated, Exo were labeled with SP-Dio_18_(3). After quenching (15 ml Exo-depleted FCS) and washing (2 × 90–120 min, 100,000 *g*) Exo were suspended in 30 ml PBS, layered over 10 ml 40% sucrose and centrifuged for 90 min at 100,000 *g*, collecting the Exo pellet at the bottom (62). Fluorescence was measured with Fluoroskan Ascent, using Ex 485 nm/Em 538 nm filter pairs.

### Pulldown Procedure

Membrane lysates (5 mg) were incubated (o/n, 4°C) with 1 ml pre-swollen CNBr-activated Sepharose (GE Healthcare, Munich, Germany) (1 mM HCl, 4°C) in coupling buffer (0.1 M NaHCO_3_, 0.5 M NaCl, pH 8.3). After washing, free binding sites were blocked (1 M Tris, pH 9.0, 6 h, 4°C). Coupling efficacy was 60–80%. Lysed (Lubrol) Exo membranes (1 mg) were passed. Bound proteins were eluted (50 mM glycine, pH 2.5). Before loading, columns were washed two times with elution buffer (62).

### Protein Elution Tryptic Digestion and Mass Spectrometry and Database Searches

Cells and Exo were lysed and proteins were separated by 1D SDS gel electrophoresis. After staining with Coomassie lanes were cut into 10 slices. Proteins in the individual gel slices were reduced with DTT, alkylated with iodoacetamide, and in-gel digested with trypsin (Promega, Mannheim, Germany) overnight. Tryptic peptides were extracted from the gel pieces, evaporated to dryness in a speed-vac concentrator and dissolved in 5 µl 0.1% TFA/2.5% hexafluoro-2-propanol prior to analysis by nanoLC–ESI-MS/MS.

Peptide mixtures were separated using a nanoAcquity UPLC system. For trapping, we used a C18 pre-column (180 µm × 20 mm) with a particle size of 5 µm (Waters GmbH, Eschborn, Germany). Liquid chromatography separation was performed on a BEH130 C18 main-column (100 µm × 100 mm) with a particle size of 1.7 µm (Waters GmbH, Eschborn, Germany). Peptide mixtures were loaded on the trap column at a flow rate of 5 µl/min and were eluted with a gradient at a flow rate of 400 nl/min. The nanoUPLC system was coupled online to an LTQ-Orbitrap XL mass spectrometer (Thermo Scientific, Bremen, Germany). The mass spectrometer was operated in data-dependent mode to automatically measure MS1 and MS2. Data were acquired by scan cycles of one FTMS scan with a resolution of 60,000 at *m/z* 400 and a range from 300 to 2,000 *m/z* in parallel with six MS/MS scans in the linear ion trap of the most abundant precursor ions.

The mgf-files generated by Xcalibur software (Thermo Scientific, Bremen, Germany) were used for database searches with the MASCOT search engine (version 2.4.1, Matrix Science, London, UK) against the SwissProt database (SwissProt 2015_08, 549008 sequences; 195692017 residues) with taxonomy human (20278 sequences). Peptide mass tolerance for database searches was set to 7 ppm and fragment mass tolerance to 0.4 Da. Carbamidomethylation of C was set as fixed modification. Variable modifications included oxidation of M and deamidation of NQ. One missed cleavage site in case of incomplete trypsin hydrolysis was allowed. Furthermore, proteins were considered as identified if more than one unique peptide had an individual ion score exceeding the MASCOT identity threshold (63).

### mRNA Preparation and Deep Sequencing

mRNA was extracted using the miRNeasyMinikit following the supplier’s suggestion (Qiagen, Hildesheim, Germany). Deep sequencing mRNA analysis was performed at the Core facility of EMBL, Heidelberg (ENA database, accession number: PRJEB25444). The alignment software used was STAR aligner version 2.5.2a, reference mm10. Mean values of normalized data were compared. Differential recovery was defined by ≥2-fold changes in mRNA signal strength in untreated versus MDSC-Exo-treated cells.

### Flow-Cytometry Analysis

Cells (5 × 10^5^) were stained according to routine procedures. In case of double or triple fluorescence, the same procedure was repeated with adequate antibodies and blocking steps, where required. Exo (10 µg) were coupled to 1 µl latex beads. After blocking (100 mM glycine) and washing, Exo-loaded beads were stained using the same protocol as for cells. For intracellular staining, cells or bead-coated Exo were fixed (1% formalin, 10 min on ice), washed with PBS/1% BSA, and incubated with 0.1% Tween (15 min on ice). Apoptosis was determined by AnnV/PI staining. Samples were analyzed in a FACSCalibur using the CellQuest program.

### Confocal Microscopy

Snap frozen tissue sections (8 µm) from AA mice that had received an i.v. injection of Dio-18(3)-labeled MDSC-Exo were fixed, permeabilized, and blocked. After washing, sections were counterstained with DAPI. Slides were mounted in Elvanol. Digitized images were generated using a Leica LMS800 microscope and the Carl Zeiss Vision software for evaluation.

### Trogocytosis Assay

Myeloid-derived suppressor cells (10 × 10^6^) were suspended in PBS containing 1 mg/ml Sulfobiotin-X-NHS and incubated for 10 min at 25°C (64). After adding an equivalent volume of FCS, cells were incubated for an additional 10 min at 4°C. Washed biotinylated MDSC were co-cultured with LNC at a ratio of 2:1 (2 h, 37°C). After washing (2 mM EDTA/PBS), cells were stained with APC-labeled antibody and counterstained with Streptavidin-FITC. Trogocytosis (transfer of biotinylated membrane particles) was evaluated by flow-cytometry.

### T Cell Proliferation Assay

Lymph node cells and SkIL were seeded in U-shaped 96-well plate (1 × 10^5^–2.5 × 10^4^ cells/well) in the absence or presence of MDSC at the indicated ratios or MDSC-Exo (20 µg/ml). Cells were stimulated by IL2 (100 U/ml) or 1 × 10^4^ DC loaded with keratin peptides (DC-AApept)/well. Cells were cultured for 3 days adding 3H-thymidine (10 μCi/ml) during the last 16 h. Plates were harvested and 3H-thymidine incorporation was evaluated in a β-counter. Mean cpm ± SD of triplicates (after subtracting counts for DC) are presented.

### Cytotoxicity Assay

Cytotoxic T cells activity was evaluated after *in vitro* (re)stimulation of LNC (100 U/ml IL2 or 1 × 10^5^ DC-AApept/10^6^ LNC) by 3H-thymidine release from labeled (12 h, 10 μCi/ml 3H-thymidine) syngeneic ConA blasts (10^4^/well), which were loaded with AA peptides, where indicated. Effector cells were titrated (1 × 10^6^–6 × 10^4^). After 6 h at 37°C, cells were harvested, and radioactivity was determined in a β-counter. Cytotoxicity is presented as % cytotoxicity = 100× (counts in control wells − counts in test wells)/(counts in control wells). The spontaneous release of syngeneic lymphoblasts (ConA stimulated LNC) ranged between 8 and 20%. Mean values ± SD of triplicates are presented.

### Animal Experiments

Alopecia areata was induced by skin transplantation (65). AA-affected female C3H/HeJ mice were sacrificed and recipient female C3H/HeJ mice were anesthetized by injecting 0.12–0.15 ml ketamine, i.p. Antero-posterior midline of the graft recipients was shaved. Disinfection of the skin before grafting was done with an ethanol pad. For grafting 1 cm pieces in diameter of AA-affected skin from the donor were cut and collected in PBS. Round pieces of skin from the graft recipient were removed, the graft was put onto the gap, stitches were made on four sides, gaps were sealed with histoacryl-glue applying bandages after drying. Drinking water (days 0–4, days 7–11) contained Sulfamidin (1 g/1l). Hair loss mostly starts at the snoot or the extremities or by thinning of hair in the lower belly. After 6–10 weeks mice present with AA totalis (100% hair loss) or partialis (>50% hair loss), the latter may proceed with time. AA incipient is defined as beginning hair loss (snoot, extremities, lower belly) <10–15% at 3–4 weeks after grafting. Without treatment, AA incipient progresses toward partialis or totalis. Delayed-type hypersensitivity (DTH) was induced after shaving the back of mice and sensitizing with 1% SADBE (squaric acid dibutylester) in acetone on the dorsal side 3–4 times with a cotton bud. Topical applications of 0.5% SADBE in acetone on the back and the abdominal wall was repeated three times at 10 days intervals to induce a moderately severe contact dermatitis lasting for 2–3 days. Mice were sacrificed by cervical dislocation 3 days after the last challenge. To control MDSC and MDSC-Exo distribution, naive and AA mice received a single injection of CFSE-labeled MDSC (1 × 10^7^ in 200 µl NaCl, i.v.) or 100 µg SP-Dio_18_(3)-labeled MDSC-Exo in 100 µl NaCl, i.v., sacrificing mice after 8–48h. Lymphoid organs were excised to evaluate the distribution of MDSC and MDSC-Exo. Where indicated, mice with AA partialis or AA totalis received i.v. injections of 100 µg MDSC-Exo in 100 µl NaCl, two-times/week. Control groups received 100 µl NaCl, i.v., two-times/week. AA progression and/or hair growth was controlled weekly. Animal experimentations were approved by the local governmental authorities of Baden Wuerttemberg, Germany.

### Statistics

Significance was evaluated by the two-tailed Student’s *t*-test (*in vitro* assays) or by Wilcoxon Rank sum test (*in vivo* assays). *P* values <0.05 were considered significant. Functional assays were repeated at least three times. Mean ± SD of *in vitro* studies are based on 3–4 replicates.

## Results

Myeloid-derived suppressor cells and Treg are important in tolerance maintenance, deficits in these regulatory immune cells frequently being accompanied by exacerbation of autoimmune disease. Aiming to use *in vitro* generated MDSC-Exo as a therapeutic in autoimmune diseases, we had to clarify potential differences of *in vitro* generated MDSC-Exo to MDSC-Exo isolated from healthy and diseased donors as well as potential function-relevant differences to MDSC. As autoimmune disease model AA was chosen. AA is efficiently treated by induction of a chronic contact eczema (topical application of SADBE), which promotes MDSC expansion and activation (56), where SADBE treatment can be replaced by MDSC (60).

### MDSC and MDSC-Exo Characterization

**Figure 1:**
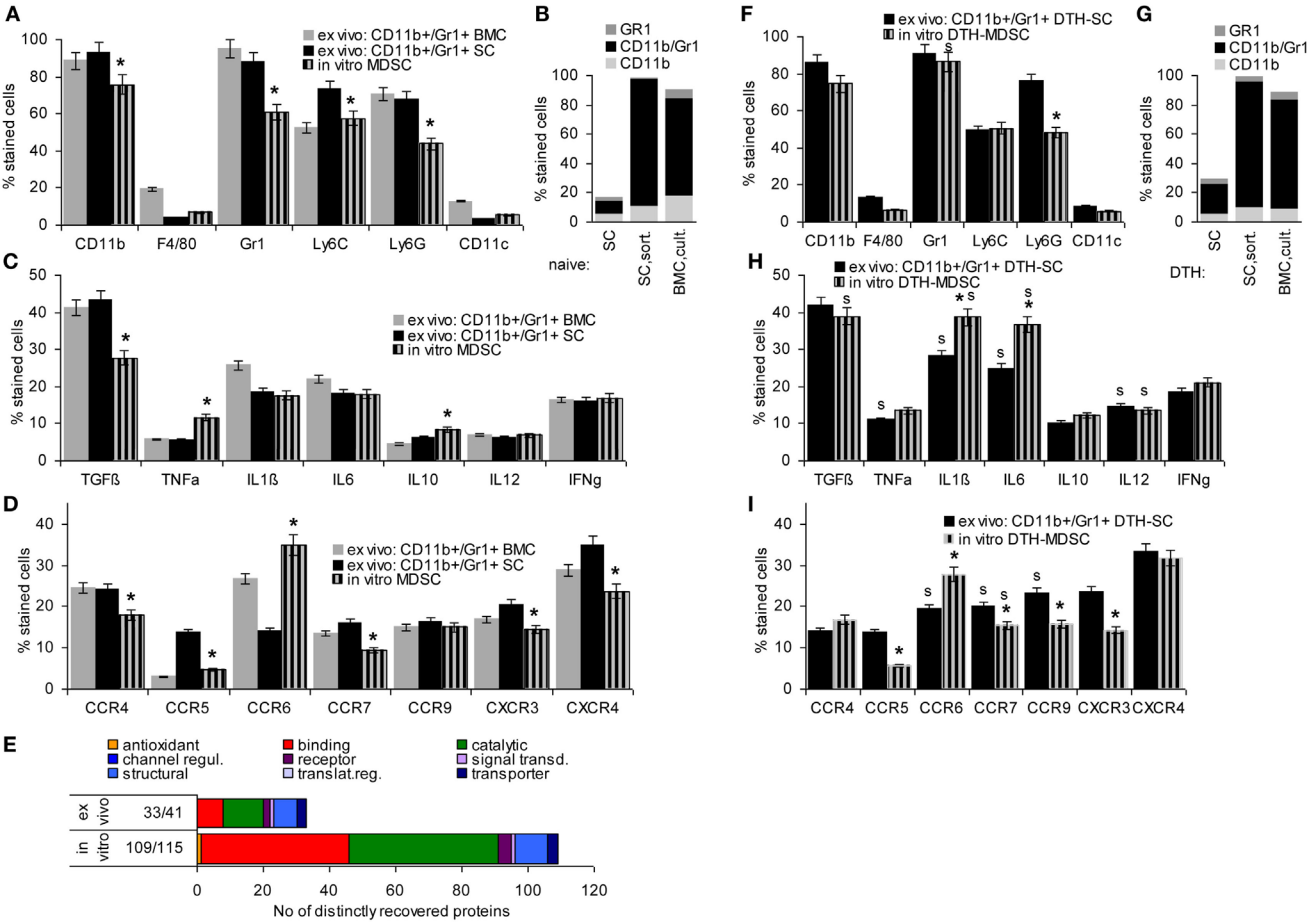
Comparison of *ex vivo* isolated versus *in vitro* generated myeloid-derived suppressor cells (MDSC). MDSC were isolated from spleen cells (SC) or bone marrow cells (BMC) by magnetic bead sorting for CD11b+Gr1+ cells or were generated *in vitro* from BMC cultured in the presence of GMCSF, IL6, and PGE2. Flow-cytometry analysis of **(A)** MDSC membrane markers, **(B)** CD11b+GR1+ versus Gr1+CD11b− and CD11b+Gr1− cells, **(C)** cytokines **(D)** chemokine receptors; mean percent stained cells ± SD of three experiments are shown; significant differences between *in vitro* generated versus *ex vivo* isolated MDSC from the spleen are indicated by *. **(E)** Proteins identified by mass spectrometry in *ex vivo* or *in vitro* generated MDSC were clustered according to molecular function (Panther Gene Analysis). The number of proteins enriched in *ex vivo* or *in vitro* generated MDSC are shown. **(F–I)** Flow-cytometry analysis of MDSC isolated from SC by sorting for CD11b+Gr1+ cells or generated *in vitro* from BMC of mice with a chronic delayed type hypersensitivity (DTH) reaction [squaric acid dibutylester (SADBE) treatment] was preformed as in **(A–D)**; mean percent stained cells ± SD of three experiments are shown; significant differences between *in vitro* generated versus *ex vivo* isolated MDSC from the spleen are indicated by *; significant differences between MDSC from naïve and DTH-affected mice are indicated by “s.” MDSC (CD11b+Gr1+) surface marker expression does not strongly differ between *ex vivo* and *in vitro* generated MDSC. There is, however, a slight decrease in TGFβ and a shift toward pronounced CCR6 expression. Similar to MDSC from naïve mice, no strong differences were seen between *ex vivo* versus *in vitro* generated MDSC from SADBE-treated mice. Instead, the lower recovery of TGFß in *in vitro* generated MDSC from naïve mice was mitigated and IL1β and IL6 expression was upregulated.

Mouse MDSC are CD11b+Gr1+, two subpopulations being differentiated by Ly6Chigh (M-MDSC) or Ly6Ghigh (G-MDSC). They have a very low expression of the mature Mϕ marker F4/80 and the DC marker CD11c. Comparing *in vitro* BMC-derived MDSC with CD11b+Gr1+ cells isolated by magnetic bead sorting from SC or BMC of naïve mice revealed that cultured BMC were strongly enriched for MDSC, nearly reaching the level of magnetic bead-sorted CD11b+Gr1+ cells with a slight preference for M-MDSC. Only ~5% expressed the mature Mϕ and a DC marker, which was similar to *ex vivo* sorted MDSC from SC and BMC (Figures 1A,B).

TGFβ, inflammatory, and immunosuppressive cytokines are important mediators of MDSC activity. Expression of TGFβ and the inflammatory cytokines TNFα, IL1β, and IL6 did not or not strongly differ between *ex vivo* sorted and culture-derived MDSC. IL10 expression was slightly higher in culture-derived MDSC. IL12 and IFNγ expression did not differ between the two MDSC preparations (Figure 1C).

Myeloid-derived suppressor cells migrate from the BM toward inflamed organs, which involves predominantly CCR7, CXCR3, and CXCR4. Chemokine receptor expression differed between *ex vivo* sorted and culture-derived MDSC with CCR5, CCR7, CXCR3, and CXCR4 expression being slightly reduced, but CCR6 expression being higher in culture- than sorting-derived MDCS (Figure 1D).

Finally, STAT6, MyD88, NFκB, HIF1α, Myc, iNOS, BclXl, and survivin are important mediators of MDSC signaling. Only iNOS and survivin were expressed slightly higher in culture- than sorting-derived MDSC (data not shown).

This overview on culture- versus sorting-derived MDSC suggesting comparability, a proteome analysis was an essential prerequisite for an estimate on functional activity. The analysis identified >1,000 proteins in culture- and sorting-derived MDSC. Using stringent conditions for distinctly recovered proteins (≥3 specific peptides, ≥2-fold difference) revealed 109 and 33 proteins solely or preferentially recovered in culture- and sorting-derived MDSC, respectively (Figure 1E; Table S3 in Supplementary Material). Classification of these distinctly recovered proteins (reactome) according to engagement in the immune system, in proliferation, transport, the cytoskeleton, binding and migration, metabolism, proteolysis, apoptosis, and signaling showed higher recovery of some proteins engaged in antigen processing and presentation, cell division, and metabolism in culture- than in sorting-derived MDSC. The abundance of molecules engaged in transcription and RNA processing in *in vitro* generated MDSC is likely due to the culture condition promoting myeloid progenitor expansion. However, besides higher expression of arginase 1 in culture-derived MDSC, differences to *ex vivo* selected MDSC were not function-relevant. This also accounts for the few proteins preferentially recovered in *ex vivo* isolated MDSC (Tables S3B,C and Figure S1A in Supplementary Material).

Chronic inflammation, like a persisting contact eczema that serves as a therapeutic in AA promotes MDSC expansion. Thus, it became important, whether *in vitro* generated MDSC differ from CD11b+Gr1+SC of SADBE-treated mice. The overview on mouse MDSC markers revealed no differences except for the lower level of Ly6Ghigh in BMC-derived MDSC and a slight increase in Gr1+ cells compared to naïve mice (Figures 1F,G). Inflammatory cytokines were mostly upregulated in *ex vivo* sorted and *in vitro* generated MDSC from SADBE-treated compared to naïve mice. In addition, IL1β and IL6 were upregulated in culture-derived MDSC (Figure 1H). Expression of the chemokine receptors CCR6, CCR7, and CCR9 was also upregulated in MDSC from SADBE-treated compared to naïve mice. CCR6 expression remained higher in *in vitro* generated than *ex vivo* sorted MDSC (Figure 1I).

The higher recovery of inflammatory cytokines in MDSC from mice with a chronic eczema was expected. Aiming for MDSC-Exo derived from healthy donor BMC, this had to be kept in mind as possibly weakening the therapeutic efficacy in autoimmune disease. On the other hand, the few differences in culture- versus *ex vivo* sorting-derived MDSC did not argue against culture-derived MDSC as Exo provider. Thus, we aimed to use MDSC-Exo collected from culture-derived BMC-MDSC of healthy mice.

**Figure 2:**
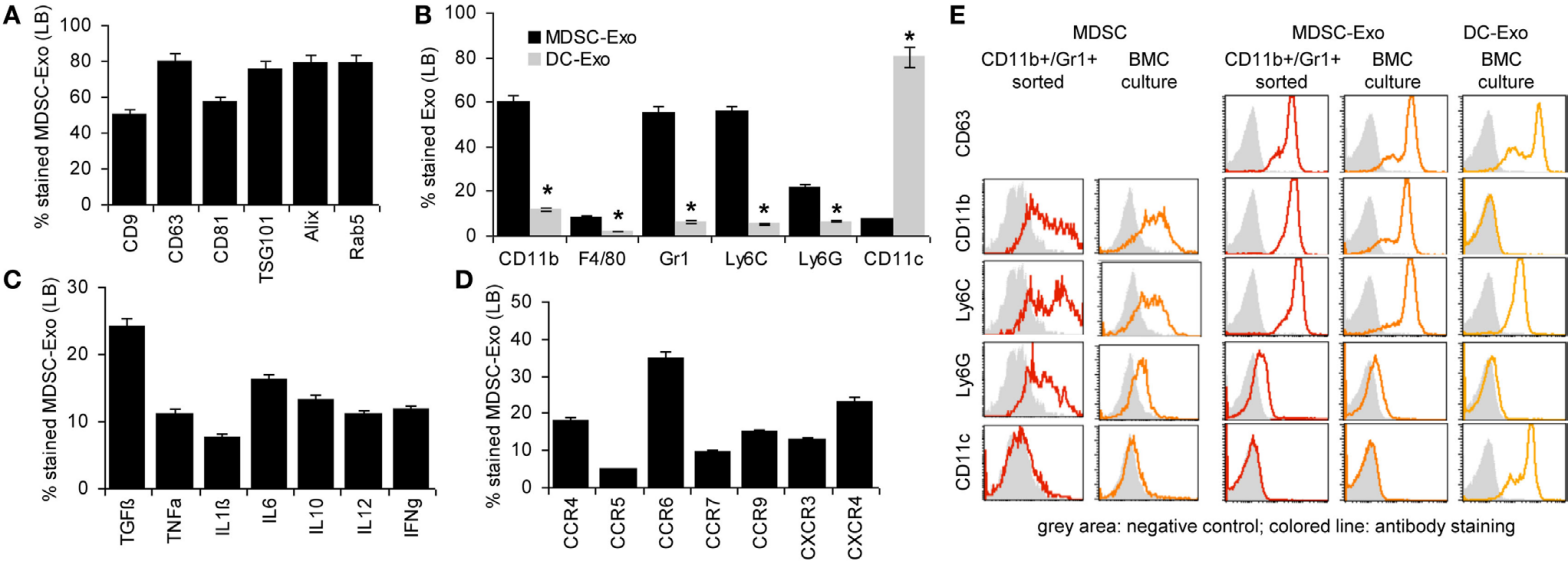
Characterization of myeloid-derived suppressor cells (MDSC) exosomes (Exo). MDSC and dendritic cells (DC) were generated *in vitro* from bone marrow cells (BMC) of naïve mice. After maturation, MDCS and DC were cultured for 2 days in medium with 3% Exo-depleted FCS. After purification, Exo were coupled to latex beads (LB). Flow-cytometry analysis of **(A)** constitutive Exo marker expression in MDSC-Exo, **(B)** MDSC surface marker expression in comparison to DC marker expression in MDSC-Exo and DC-Exo, **(C)** cytokine, and **(D)** chemokine receptor expression. Mean percent stained LB ± SD of three experiments is shown. Significant differences between MDSC-Exo and DC-Exo are indicated by *. **(E)** Overlay (flow-cytometry) of stainings of *ex vivo* sorted and BMC-culture-derived MDSC and MDSC-Exo and for comparison BMC-culture-derived DC-Exo. With the exception of constitutive Exo markers, MDSC-Exo differed from cells by a slightly reduced recovery of cytokines, whereas recovery of chemokine receptors is increased. MDSC-Exo significantly differs from DC-Exo, both being derived from cultured BMC.

To briefly introduce Exo, flow-cytometry analysis (Latex bead <LB>-coupled Exo) revealed that culture-derived MDSC-Exo express the most prominent constitutive Exo marker like tetraspanins, TSG101, Alix, and rab5 (Figure 2A). MDSC-Exo also abundantly express the MDSC markers, such as CD11b, Gr1, Ly6C and, less abundantly, Ly6G. They poorly express F4/80 and CD11c, which clearly differentiates them from BMC-derived DC-Exo (Figure 2B). They express TGFβ and inflammatory cytokines and, at a high level chemokine receptors. Like in BMC-derived MDSC, highest expression is seen for CCR6, but the expression of CXCR3 and CXCR4 is also high (Figures 2C,D). An overlay example of a flow-cytometry analysis of MDSC and MDSC-Exo confirms the comparability of the standard MDSC markers in cells and Exo (Figure 2E). A proteome analysis did not uncover major differences between MDSC-Exo collected from sorting- versus culture-derived MDSC-Exo. This accounted for the overall distribution according to molecular functions, where it should be noted that 321 from 397 proteins were identified in both MDSC-Exo populations and only 46, respectively, 44 were distinctly expressed in Exo collected from *in vitro*- versus *ex vivo*-derived MDSC (Table S4, Figure S1B in Supplementary Material). Grouping the distinctly recovered proteins according to Reactome pathways into immune response engagement, signaling, and transcription/translation revealed that only proteins engaged in cell division were far more abundant in culture-derived MDSC-Exo (Figure S1C in Supplementary Material). We interpret this finding as a result of the culture condition-promoted expansion of MDSC. Irrespective of this difference, the finding justifies the use of culture-derived MDSC-Exo in therapy.

There remained the question on the comparability of MDSC-Exo versus MDSC. Expectedly, a smaller number of proteins (~50%) were recovered from MDSC-Exo than MDSC. This was independent of whether the Exo were collected from sorted- or cultured-derived MDSC. Also, no major differences were seen with respect to the molecular functions (Figure S2A in Supplementary Material). Furthermore, there was a large overlap of MDSC and MDSC-Exo proteins, which was consistent for both MDSC-Exo preparations (Tables S5A,B and Figure S2B in Supplementary Material). Thus, we searched in detail for differentially expressed proteins that are engaged in immune response regulation. This revealed 75 proteins enriched in MDSC and 32 in MDSC-Exo. The majority of the engaged proteins was also engaged in immune system-related signaling cascades. The latter included Arg1, BAX, and SOD2, which are MDSC function-relevant proteins and were recovered at a lower level in MDSC-Exo than in MDSC. On the other hand, complement and proteasome components and tetraspanins were more abundant in MDSC-Exo (Figure S2C in Supplementary Material).

Taken together, MDSC-Exo express the standard mouse MDSC markers. Enrichment of tetraspanins and other molecules engaged in Exo biogenesis, vesicle trafficking, and vesicle-mediated transport as well as the abundance of complement (C’) components and proteasome subunits meet expectation (66). Abundance of the latter could possibly cope with higher level of Arg1, SOD2, and BAX expression in MDSC. High recovery of tetraspanins and chemokine receptors could facilitate targeting (62) and homing (67, 68). Thus, we proceeded searching for preferred targets and target cell structures of MDSC and MDSC-Exo as a starting point to explore the impact of MDSC versus MDSC-Exo on immune response regulation in health and AA.

### Preferred Targets of MDSC and MDSC-Exo

Targets of MDSC were evaluated by trogocytosis, analyzed as the transfer of biotin from MDSC to target cells. Targets of MDSC-Exo were evaluated by flow-cytometry measuring uptake of Dio-labeled MDSC-Exo.

**Figure 3:**
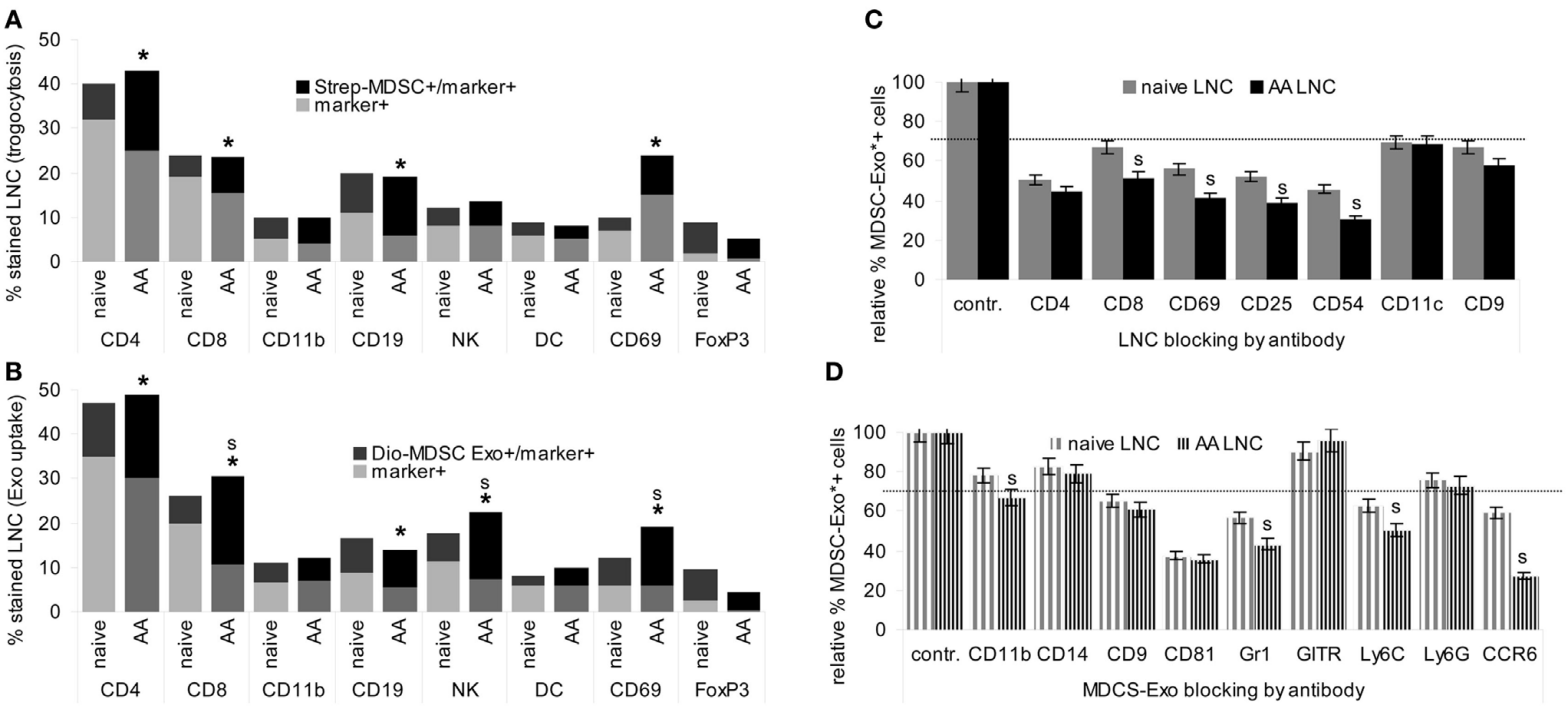
Targets of myeloid-derived suppressor cells (MDSC) and MDSC-Exo. MDSC and MDSC-Exo were generated from bone marrow cells (BMC) of naïve mice as described above. MDSC were biotinylated and MDSC-Exo were labeled with Dio_18_(3). MDSC and MDSC-Exo were cocultured with lymph node cells (LNC) of naïve and alopecia areata (AA) mice for 2 h at 37°C. MDSC/MDSC-Exo targets were evaluated by flow-cytometry defining **(A)** recovery of biotin (trogocytosis) in leukocyte subpopulations and **(B)** recovery of Dio_18_(3) in leukocyte subpopulations that were identified by counterstaining with the indicated markers. The experiment was run in triplicates and was repeated three times. Mean percent of marker+ and marker+/biotin+, respectively, marker+/Dio_18_(3)+ LNC of triplicates from three experiments are shown. Significant differences between naïve and AA mice are indicated by *, significant differences between trogocytosis and Exo uptake are indicated by “s.” **(C)** LNC of naïve and AA mice were incubated with the indicated antibodies for 30 min at room temperature. After washing, cells were incubated with Dio_18_(3)-labeled MDSC-Exo for 2 h at 4°C. **(D)** Dio_18_(3)-labeled MDSC-Exo were incubated with the indicated antibodies for 2 h at 4°C. Thereafter Dio_18_(3)-labeled MDSC-Exo were purified by ultracentrifugation through a 40% sucrose cushion. After washing the Exo pellet in PBS, the Dio_18_(3)-labeled Exo were incubated with LNC of naïve and AA mice for 4 h at 4°C. **(C,D)** The percent Dio_18_(3)-labeled cells was evaluated by flow-cytometry. Uptake by cells or Exo incubated with a control IgG was taken as 100%. The mean percent of Dio_18_(3)-label stained cells ± SD of triplicates from two experiments is shown. 30% reduction was judged as significant (dashed line), significant differences between naïve and AA LNC are indicated by **s**. MDSC and MDSC-Exo bind preferentially to regulatory T cells. They also bind to CD4+ cells, CD8+ cells, B cells, Mϕ, NK, and, weakly, dendritic cells. MDSC binding and particularly MDSC-Exo uptake is significantly stronger in activated AA T cell. All ligands for MDSC-Exo uptake, CD4, CD8, CD69, CD25, and CD54 are components of the immunological synapse. The most prominent MDSC-Exo receptor is CD81; Gr1/Ly6C and CCR6 also contribute to binding.

In cocultures, LNC took up biotin-labeled MDSC fragments. In lymphoid cells from naive mice uptake was the highest for FoxP3+ cells, followed by CD19+ B cells and a few CD69+ cells. In AA mice, a strong increase in uptake by CD4+ and a significant uptake by CD69+ cells was noted. The smaller population of FoxP3+ cells maintained the lead in uptake (Figure 3A).

Similar to MDSC trogocytosis, MDSC-Exo uptake was the highest for FoxP3+ cells, and uptake by CD4+, CD8+, CD69+, and NK was increased in AA compared to naïve LNC. Furthermore, MDSC-Exo uptake by CD8+, NK+, and CD69 + LNC significantly exceeded trogocytosis (Figure 3B).

To obtain a hint toward the target structure for MDCS-Exo, LNC, or Dio_18_(3)-labeled MDSC-Exo were preincubated with the indicated antibodies. After washing, LNC were incubated for 2 h at room temperature with the Dio_18_(3)-labeled MDSC-Exo. The percent of Exo+ cells was compared to Exo+ cells incubated with a control IgG. Obviously, MDSC-Exo target different molecules to enter T cells, DC, NK, Mϕ, and B cells, with CD4, CD69, CD25, and CD54 being the most efficient targets in activated T cells, whereas blocking of DC by anti-CD11c did not differ between naïve and AA LNC (Figure 3C). Blocking Mϕ with anti-CD11b, NK with anti-CD314, and B cells with anti-sIgM in SC from naïve and AA mice showed borderline significant MDSC-Exo uptake inhibition, which was alike in naïve and AA LNC (data not shown). MDSC-Exo mostly used CD81, Gr1/Ly6C, and CCR6 for LNC binding. Notably, antibody blocking of MDSC-Exo by anti-Gr1/Ly6C and most pronounced anti-CCR6 is significantly stronger, when targeting AA than naïve LNC (Figure 3D).

In brief, MDSC-Exo preferentially bind with FoxP3+ cells and activated T cells. Uptake by target cells is efficiently blocked by anti-CD54, -CD4, -CD25, and -CD69. MDSC-Exo binding involves the tetraspanin CD81, Ly6C, and CCR6. The antibody inhibition of MDSC-Exo uptake by several components of the TCR complex, as well as the stronger inhibition on activated T cells pointed toward MDSC-Exo binding to the TCR complex/the immune synapse such that blocking an individual component suffices to interfere with MDSC-Exo binding. This could explain the stronger antibody inhibition in AA LNC, where CD25 and CD69 are expressed at a higher level. The engagement of tetraspanins in Exo binding is known (62). The contribution of Ly6C may be MDSC-Exo-specific. The target structures that account for MDSC-Exo CCR6 binding, particularly to AA LNC remain to be explored.

During Exo biogenesis, most components of the invaginated membrane domain are retained. Thereby, Exo become enriched for components of the vesicle transport machineries, besides other proteins. As the lipid composition of invaginated membrane microdomains, particularly of tetraspanin-enriched microdomains is known as a signaling platform that harbors preferentially palmitoylated or myristoylated signaling pathway components (69), we considered it important to obtain a first hint toward the transfer of MDSC-Exo components into LNC. To answer the question, MDSC-Exo were mildly lysed (Lubrol) to avoid disruption of membrane/membrane-attached protein complexes. Lysed MDSC-Exo were passaged over Sepharose coupled LNC membrane lysates.

Lymph node cells membranes retained 192 MDSC-Exo membrane lysate proteins. The pulldown proteins contained a relatively high number of structural components and transporters (Figure S3A in Supplementary Material). Analyzing their preferential location in MDSC and engagement in Exo biogenesis revealed an enrichment of proteins contributing to complex formation, those engaged in vesicle biogenesis including vesicle loading and transport as well as those supporting synapse formation (Table S6 in Supplementary Material). Reactome clustering according to Exo biogenesis, transport, cytoskeleton, and immune response with emphasis on an engagement in signal transduction uncovered that there is an abundance of Exo membrane-attached proteins that are engaged in signal transduction, particularly those engaged in immune response. Finally, possibly due to the special composition of the Exo lipid bilayer, proteins engaged in lipid metabolism were also abundant (Figure S3B in Supplementary Material).

These features are well in line with protein complexes engaged in Exo biogenesis remaining attached/incorporated into Exo, which includes molecules engaged in assembling the special lipid composition of the Exo membrane. Importantly, MDSC-Exo membranes contain proteins that may affect signal transduction in target cells.

### Phenotypic and Functional Changes of Leukocytes Cocultured With MDSC and MDSC-Exo

Myeloid-derived suppressor cells are supposed to interfere with T cell and NK activation and to induce Treg expansion (12, 13).

**Figure 4:**
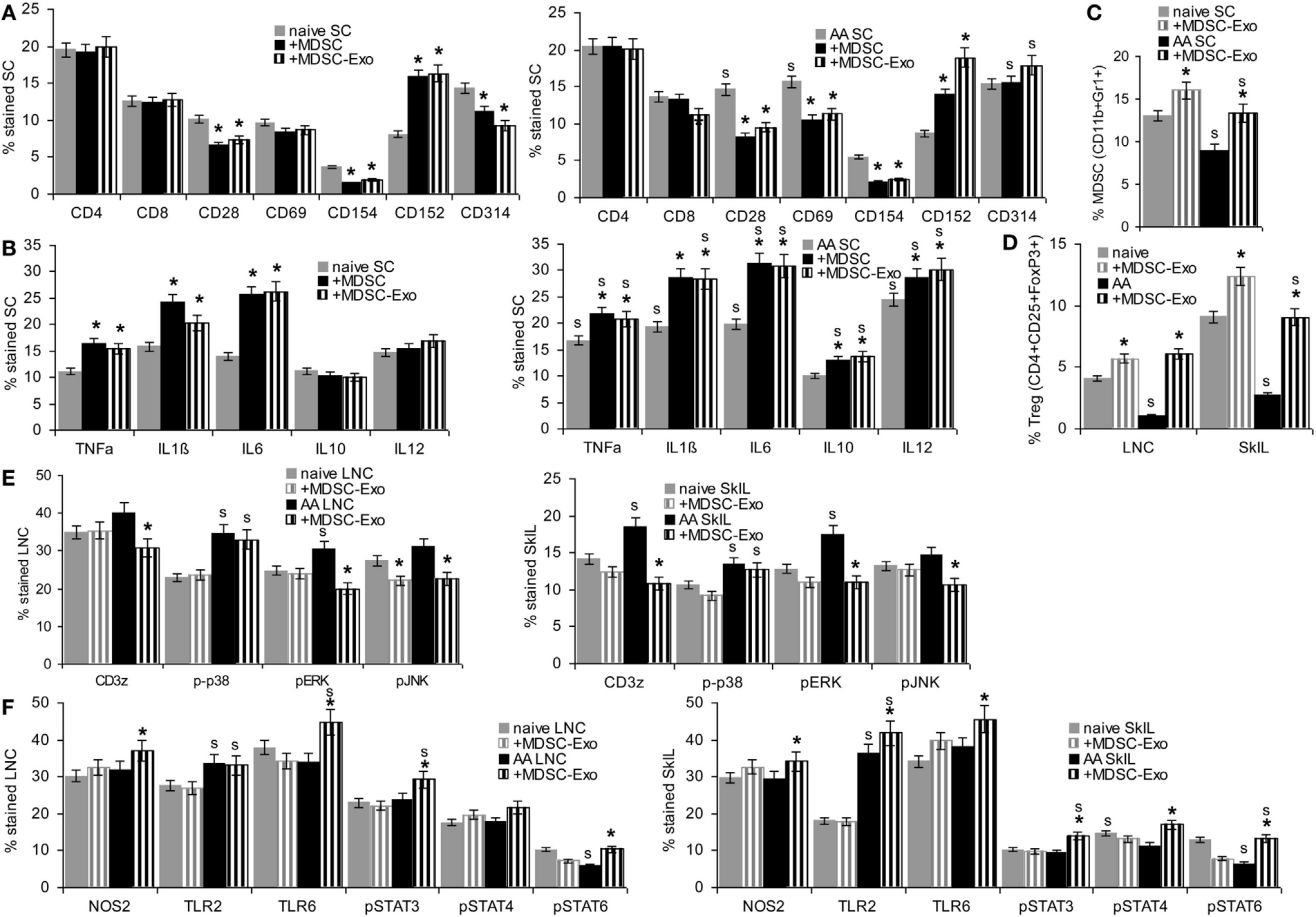
The impact of myeloid-derived suppressor cells (MDSC) and MDSC-Exo on leukocyte subpopulations and leukocyte activation. Lymph node cells (LNC), spleen cells (SC), and skin-infiltrating leukocytes (SkIL) of naïve and alopecia areata (AA) mice were cocultured for 2 days with MDSC or MDSC-Exo in RPMI/10%FCS/10^−3^M HEPES supplemented with NEAA and 10 U IL2/ml. Flow-cytometry analysis of **(A)** leukocyte subpopulations in SC and **(B)** cytokine expression in SC; significant differences by coculture with MDSC or MDSC-Exo are indicated by *, significant differences between naïve and AA leukocytes are indicated by “s.” **(C–F)** Flow-cytometry analysis of naïve and AA leukocytes after coculture with MDSC-Exo; **(C)** MDSC cells in SC and **(D)** regulatory T cells (Treg) in LNC and SkIL, **(E)** TCR signaling molecules in LNC and SkIL, **(F)** signaling molecules in innate immune responses, particularly MDSC, in LNC and SkIL. **(A–F)** Mean percent stained cells ± SD of three experiments are shown; significant differences by coculture with MDSC-Exo are indicated by *; significant differences between naïve and AA leukocytes are indicated by “s”. In naïve and AA mice, MDSC and MDSC-Exo affect T cell activation with a strong reduction of the accessory molecules, such as CD28, CD69, and CD154 and an increase in CD152 expressing cells, NK receptor CD314 expression also is slightly decreased in naïve SC. There is an increase in cells expressing inflammatory cytokines. Only in AA mice, a higher percentage of cells expresses IL10 and IL12. MDCS-Exo promote the expansion of MDSC in naïve and AA mice, which are significantly reduced in AA compared to naïve mice. This also accounts for Treg in LNC and SkIL. AA LNC showed upregulated expression of several T cell signaling molecules, MDSC-Exo exerted a minor impact on ζ-chain expression, ERK1/2 and JNK phosphorylation in AA LNC and SkIL. Except for upregulated TLR2 expression, AA LNC and SkIL did not strongly differ from that of naive mice. MDSC-Exo affected TLR6, Stat3, Stat4, and Stat6 activation.

Myeloid-derived suppressor cells and MDSC-Exo were cocultured for 2 days with lymphocytes from naïve and AA mice. Changes in marker expression were evaluated by flow-cytometry. In cocultures with MDSC, lymphocytes were separated by gating (smaller, less granulated). MDSC or MDSC-Exo did not affect the percentage of CD4+ and CD8+ T cells. Instead, MDSC and MDSC-Exo promoted a decrease in CD28+, CD69+ (only AA), and CD154+ and an increase in CD152+ T cells. NKD-ligand expression was slightly reduced (only naïve) (Figure 4A). TNFα, IL1β, and IL6 expression was increased after coculture with MDSC or MDSC-Exo; IL10 and IL12 expression was only affected in AA lymphocytes (Figure 4B). There was a slight increase in MDSC in the spleen of naïve and AA mice cocultured with MDSC-Exo. An increase in Treg was strongest in LNC and SkIL of AA mice (Figures 4C,D). MDSC-Exo particularly affected signaling in AA LNC and, even more in AA SkIL. CD3ζ chain expression, ERK and JNK phosphorylation became reduced (Figure 4E). Differences in innate immune system signaling cascades were less pronounced, where particularly high expression of TLR2 in AA SkIL should be noted. MDSC-Exo promoted a slight upregulation of TLR6 as well as of STAT3, -4, and -6 phosphorylation, mostly in AA SkIL. NOS2 expression was slightly increased in AA LNC and SkIL (Figure 4F). Expression and activation of other components of TCR signaling pathways and innate immune cell signaling were not affected (data not shown).

**Figure 5:**
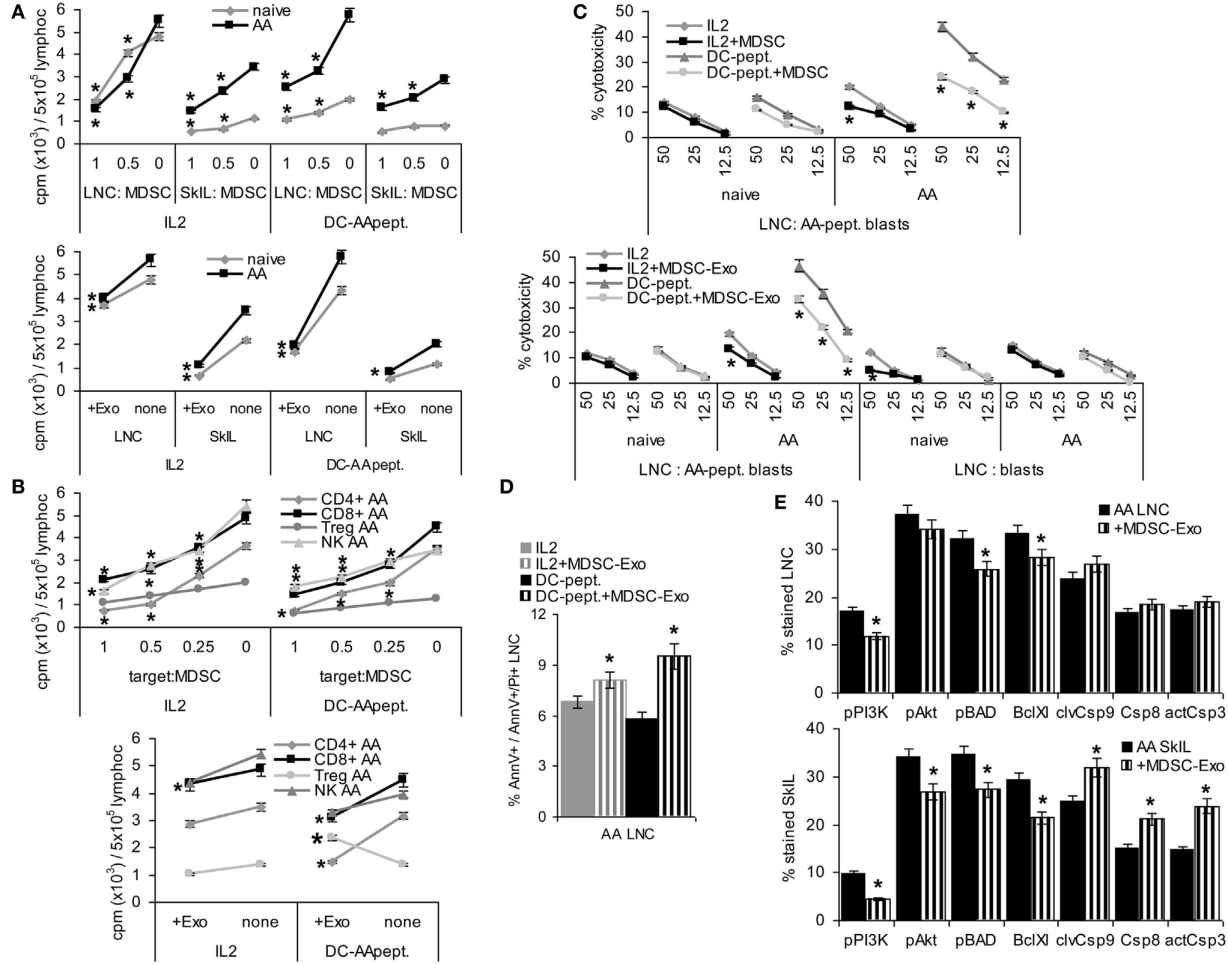
Myeloid-derived suppressor cells (MDSC) and MDSC-Exo suppress lymphocyte proliferation and cytotoxicity. Lymph node cells (LNC) and skin-infiltrating leukocytes (SkIL) of naïve and alopecia areata (AA) mice were cocultured with MDSC or MDSC-Exo. LNC and SkIL were stimulated by 100 U IL2/ml RPMI/10%FCS/10^−3^M HEPES supplemented with NEAA or by 1 × 10^5^ dendritic cells (DC)-AApept/10^6^ LNC in RPMI/10%FCS/10^−3^M HEPES supplemented with NEAA. **(A)** LNC and SkIL of naïve and AA mice were cocultured with MDCS or MDSC-Exo from naïve mice; **(B)** CD4+ cells, CD8+ cells, NK and regulatory T cells (Treg) (CD4+CD25high) of AA mice were cocultured with MDSC or MDSC-Exo. **(A,B)** Cells were cultured for 3 days in the presence of 100 U/ml IL2 or DC-AApeptide adding 3H-thymidine during the last 16 h of culture. 3H-thymidine incorporation was evaluated in a β-counter. CPM/5 × 10^5^ cells ± SD of triplicates after correction for incorporation by MDSC and/or DC are shown. Significant differences to 3H-thymidine incorporation in the absence of MDSC/MDSC-Exo are indicated by * **(C)** LNC were (re)stimulated *in vitro* with IL2 or DC-AApept for 8 days in the presence of MDSC or MDSC-Exo. (Re)stimulated lymphocytes were seeded on 3H-thymidine labeled AApeptide-loaded or unloaded syngeneic blasts. After 6 h of coculture the recovery of 3H thymidine labeled target cells was evaluated. The percent lysed target cells ± SD of triplicates is shown. Significant differences in lysis by (re)stimulation in the presence of MDSC or MDSC-Exo are indicated by *. **(D,E)** AA LNC and SkIL were cocultured for 2 days with MDSC-Exo in the presence of IL2 or DC-AApept. **(D)** Apoptosis was evaluated by flow-cytometry after AnnV-APC/PI staining. The % AnnV and AnnV/PI stained cells ± SD of triplicates is shown; **(E)** Flow-cytometry analysis of major components of the PI3K/Akt pathway and central caspases in AA LNC and SkIL. Significant differences in apoptosis, the PI3K/Akt pathway and caspases by coculture with MDSC-Exo are indicated by *. MDSC and MDSC-Exo suppress the proliferation of naïve and AA lymphocytes in response to IL2. Suppression of AA lymphocytes is stronger in response to DC-AApept. MDSC suppress proliferation of CD4+ and CD8+ T cells and NK of AA mice in response to IL2 and DC-AApept. Only in response to IL2, MDSC-Exo weakly suppress NK proliferation. MDSC-Exo suppress T cell proliferation in response to DC-AApept, but support proliferation of Treg in response to DC-AApept. Furthermore, MDSC and MDSC-Exo interfere with cytotoxic activity of AA-specific cytotoxic T cells and increase AA LNC apoptosis, which is accompanied by slightly impaired activation of the PI3K/Akt pathway. Only in AA SkIL, caspase 3, 8, and 9 expressions became slightly upregulated.

These phenotypic changes were accompanied by altered T cell activities. LNC of naïve and AA mice were cultured in the presence of MDSC or MDSC-Exo. Cells were stimulated by IL2 or AA peptide-loaded DC. In the presence of IL2, MDSC and MDSC-Exo suppressed the proliferation of naive and AA LNC and SkIL, proliferation of AA SkIL exceeding that of naïve SkIL. When stimulated with DC-AApept, the proliferative response of AA LNC and SkIL significantly exceed that of naïve LNC and SkIL and MDSC and MDSC-Exo more efficiently inhibited AA than naïve LNC and SkIL proliferation (Figure 5A). To define which leukocyte subpopulation become affected by MDSC/MDSC-Exo, CD4+ cells, CD8+ cells, NK, and Treg (CD4+ CD25high) of AA LNC were enriched by magnetic bead sorting in advance of coculture. MDSC suppressed the proliferation of CD4+, CD8+ cells of AA mice in response to IL2 and DC-AApept; NK proliferation was only affected in IL2-containing cultures. MDSC did not affect low proliferation of Treg. MDSC-Exo did not significantly affected proliferation of leukocyte subpopulations in response to IL2. Instead, MDSC-Exo efficiently suppressed proliferation of CD4+ and CD8+ AA LNC in response to DC-AApept. Notably, MDSC-Exo promoted AA Treg proliferation (Figure 5B).

Cytotoxic T cells activity was evaluated after (re)stimulation of naïve and AA LNC for 10 days with IL2 or DC-AApept. MDSC suppress AA CTL that was restimulated *in vitro* with DC-AApept. CTL of naive mice respond poorly to the primary *in vitro* stimulation with DC-AApept, which was hardly suppressed by MDSC. MDSC either do not or weakly suppress low cytotoxic activity of LNC stimulated by IL2. Similar findings account for MDSC-Exo. The low cytotoxic response to IL2 is not suppressed. Cytotoxic activity of LNC from AA mice restimulated with DC-AApept is significantly reduced, although weaker than by MDSC. Syngeneic blasts from C3H/HeJ mice are not affected significantly (Figure 5C). A potential impact of MDSC-Exo on apoptosis was evaluated after 2 days of culture in the presence of IL2 or DC-AApept. Apoptosis was measured by flow-cytometry after staining with AnnV and PI. MDSC-Exo promoted apoptosis, particularly in cultures containing DC-AApept (Figure 5D). Changes in the PI3K/Akt pathway, engaged in apoptosis protection, were mostly seen in SkIL, where PI3K and slightly Akt and BAD phosphorylation were reduced. This was accompanied by a decrease in BclXl. Also, the initiator Casp9 and weakly Casp8 and the effector Casp3 were upregulated in AA SkIL after coculture with MDSC-Exo. In draining LNC changes in the PI3K pathway were weaker and caspase upregulation was not significant (Figure 5E).

Myeloid-derived suppressor cells and, slightly less efficiently MDSC-Exo preferentially alopecia areata (AA) effector cells. Thus, we proceeded to search for *in vivo* efficacy.

### MDSC-Exo Homing

Homing of MDSC-Exo is a prerequisite for *in vivo* activity. Mice received an i.v. injection of CFSE-labeled MDSC or Dio-labeled MDSC-Exo. Recovery of cells and Exo was followed for 48 h in the naive and the AA host by flow-cytometry of dispersed lymphoid organs and confocal microscopy of shock frozen tissue sections.

**Figure 6:**
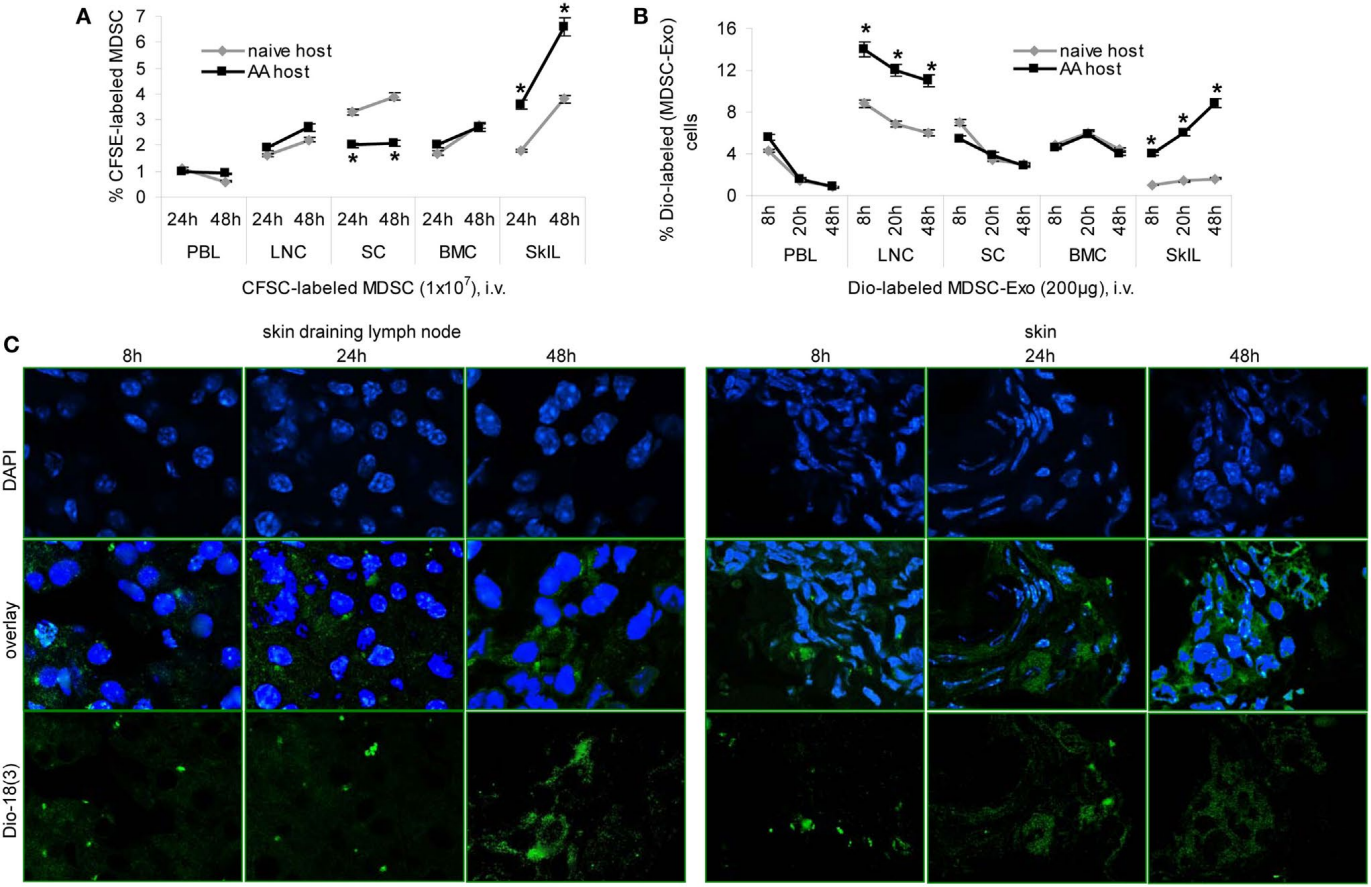
Myeloid-derived suppressor cells (MDSC) and MDSC-Exo homing *in vivo*. **(A)** Naïve and alopecia areata (AA) mice received an i.v. injection of carboxyfluorescein-succinimidylester (CFSE)-labeled MDSC or **(B)** of Dio_18_(3)-labeled MDSC-Exo. **(A,B)** Mice were sacrificed after 8–48 h, lymphoid organs were excised, dispersed, and recovery of CFSE-labeled or Dio_18_(3)-labeled cells was evaluated by flow-cytometry. The mean percent of CFSE-labeled or Dio_18_(3)-labeled cells ± SD of three mice/group is shown. Significant differences between naïve and AA lymphocyte are indicated by *. **(C)** Skin-draining LN and skin of AA mice that received Dio_18_(3)-labeled MDSC-Exo were excised after 8–48 h and shock frozen. Sections (10 µm) were stained with DAPI. Representative examples of DAPI staining, Dio_18_(3)-label recovery and overlays are shown. MDSC and MDSC-Exo preferentially home into skin-draining LN and the skin of AA mice, where they are retained for at least 48 h. In the skin, MDSC-Exo is preferentially recovered near remnant hair follicles.

Recovery of CFSE-labeled MDSC was slightly higher in skin-draining LNC and significantly higher in SkIL of AA than naive mice. Recovery in PBL, SC, and BMC did not differ between naive and AA mice. This accounted for the recovery after 24 and 48 h (Figure 6A). Dye-labeled MDSC-Exo injected i.v. are rapidly cleared from the blood, are enriched in draining LNC, and are retained in SkIL of AA mice for up to 48 h. In other organs, MDSC-Exo recovery declined after 8 and 24 h, respectively (Figure 6B). MDSC-Exo particularly located at the periphery of rudimentary hair follicles, where CD8+ cells are enriched (Figure 6C).

Thus, in AA mice MDSC-Exo are recruited with high efficacy toward activated T cells.

### AA Therapy by MDSC-Exo and *In Vivo* Impact on Immune Response Regulation

Mice with AA totalis, partialis, or incipient received 2×/week 100 µg MDSC-Exo, i.v. Treatment was maintained for 10 weeks, controlling after 6 weeks the impact on immune response regulation.

**Figure 7:**
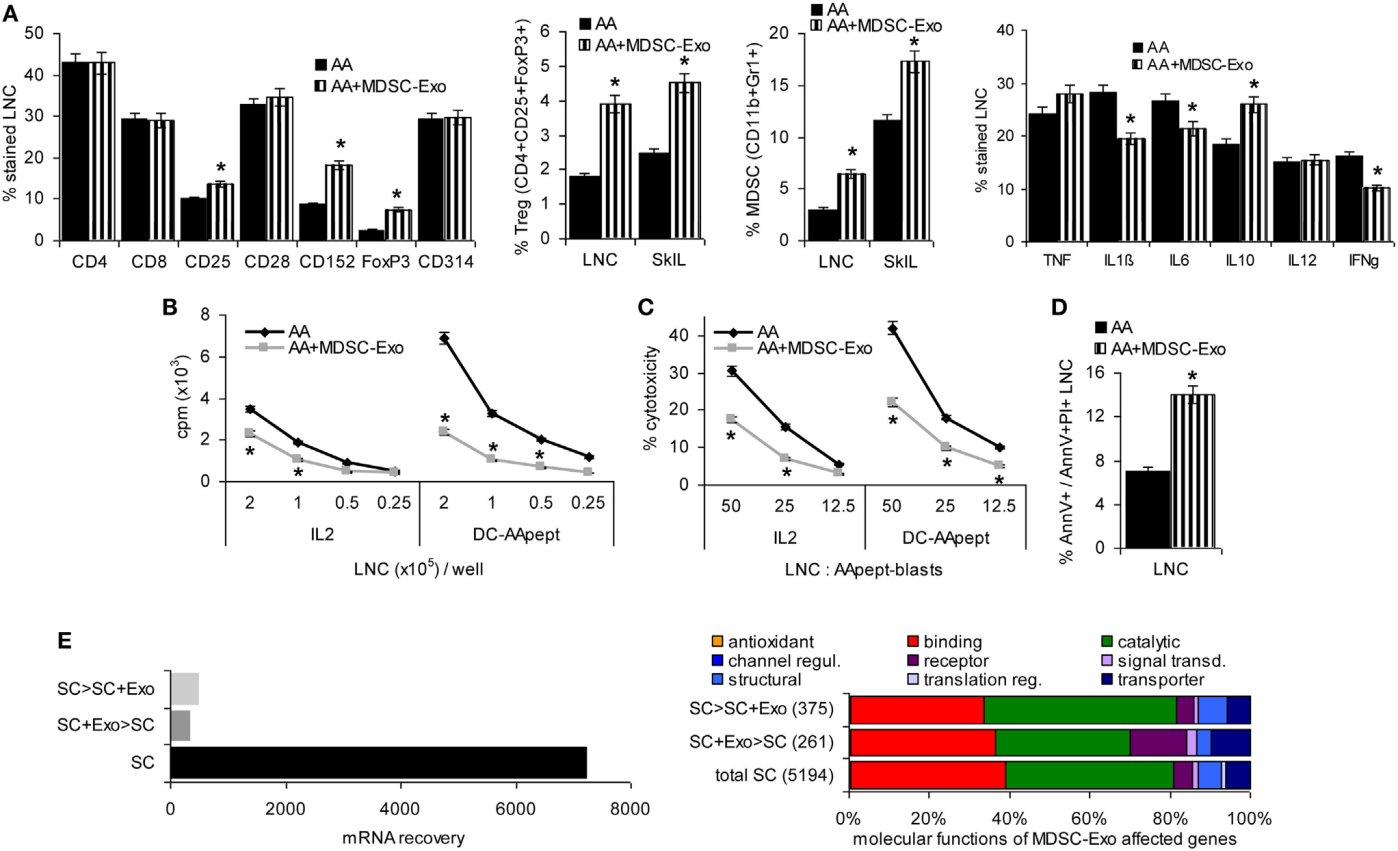

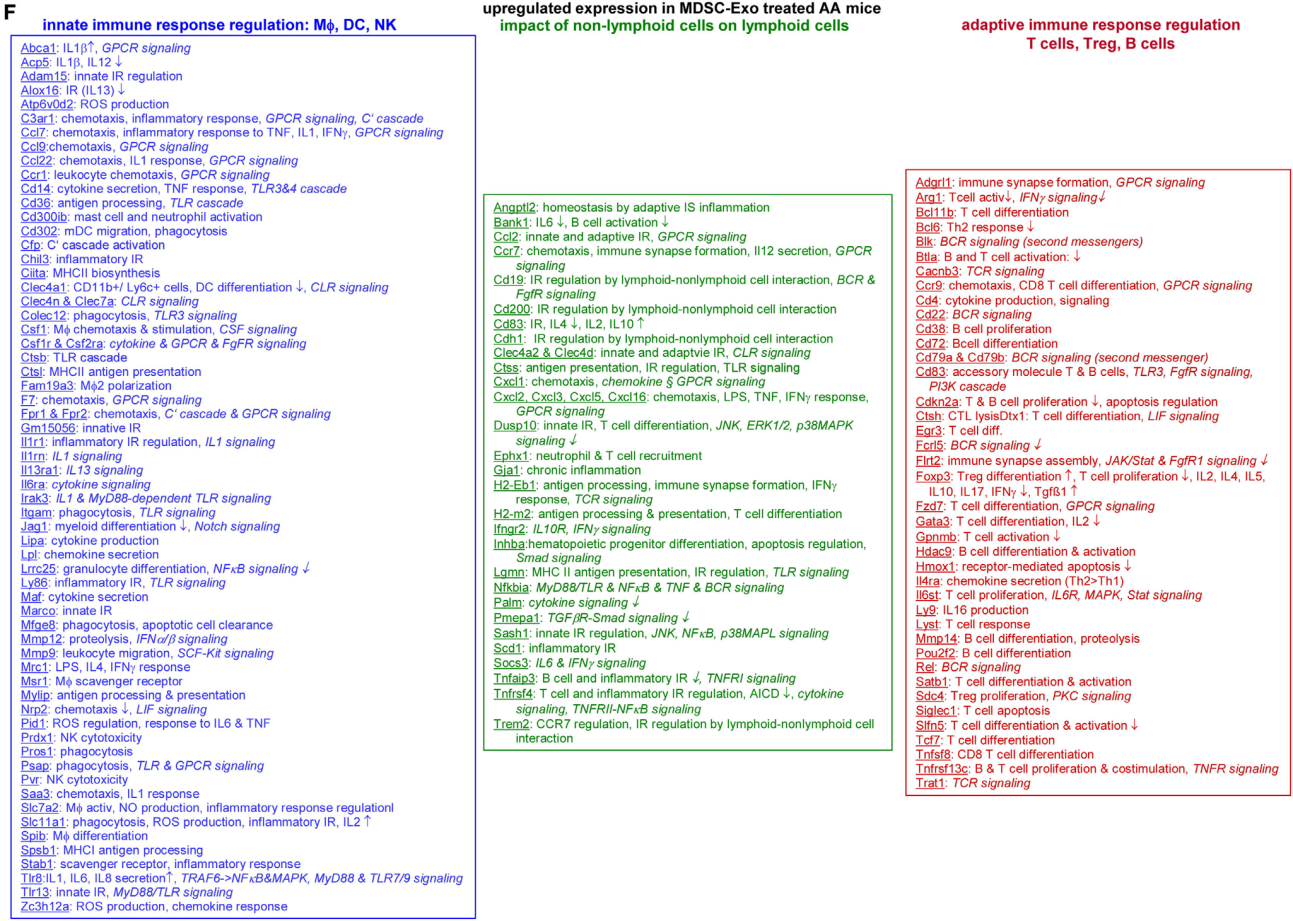

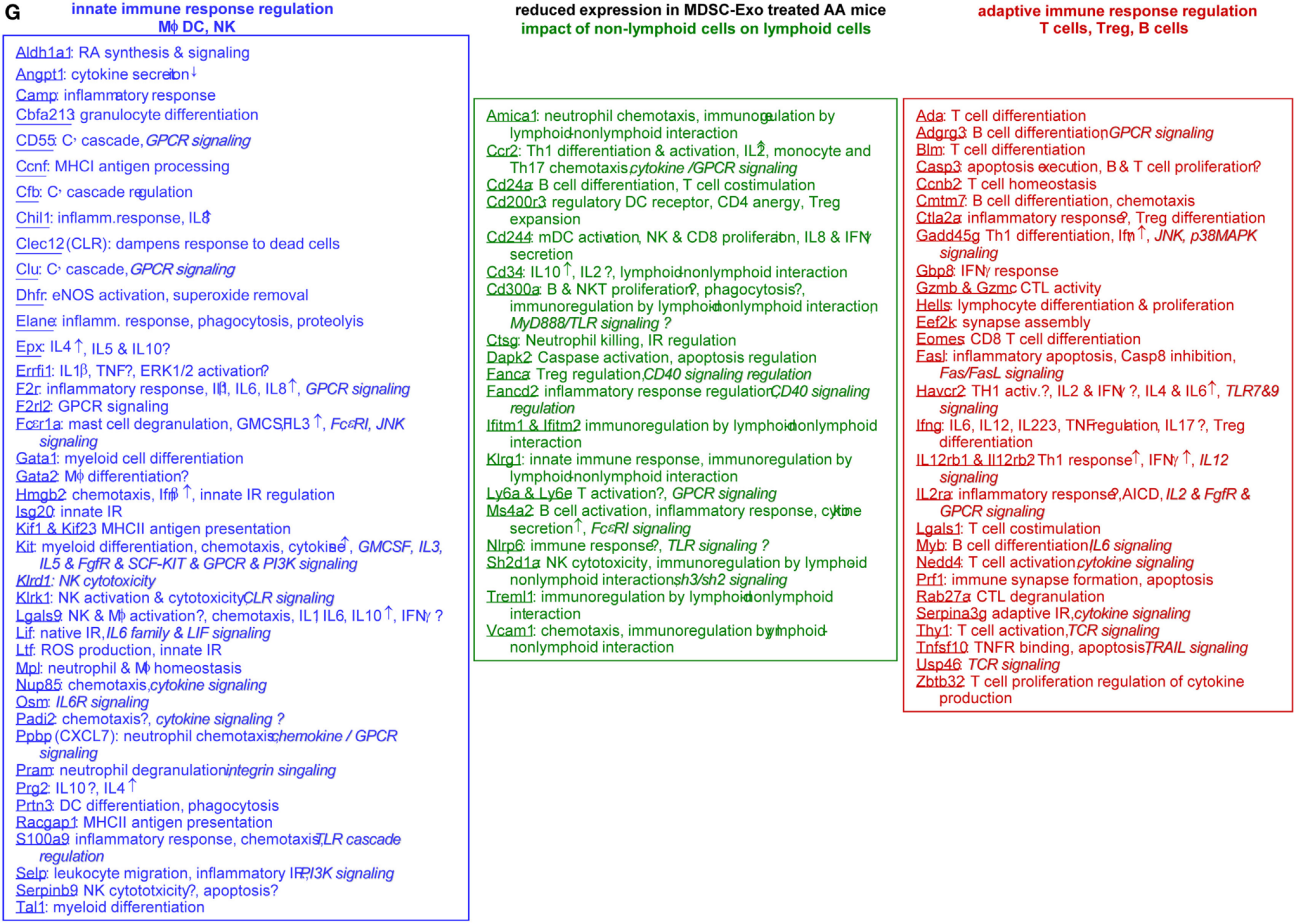
The impact of repeated myeloid-derived suppressor cells (MDSC) exosomes (Exo) application on AA leukocyte activation. Alopecia areata (AA) mice received i.v. injections of MDSC-Exo (100 μg/mouse, 2×/week) or NaCl. Mice were sacrificed after 6 weeks. **(A)** Flow-cytometry evaluation of leukocyte markers, regulatory T cells (Treg), MDSC, and cytokines in lymph node cells (LNC), or LNC and skin-infiltrating leukocytes was performed immediately after harvesting and preparation. For evaluating cytokine expression, cells were fixed and permeabilized in advance; the % stained cells ± SD of three mice/groups is shown. Significant differences by MDSC-Exo treatment are indicated by *. **(B)** LNC of untreated or MDSC-Exo-treated mice were cultured for 3 days in the presence of 100 U/ml IL2 or dendritic cells (DC)-AApept adding 3H-thymidine during the last 16 h of culture. 3H-thymidine incorporation was evaluated in a β-counter. CPM ± SD of triplicates are shown; significant differences to 3H-thymidine incorporation in lymphocytes from untreated AA mice are indicated by *. **(C)** LNC of untreated and MDSC-Exo-treated AA mice were restimulated *in vitro* with IL2 or DC-AApept for 8 days in the presence of MDSC-Exo. Lymphocytes were seeded on 3H-thymidine labeled AApeptide-loaded syngeneic blasts. After 8 h of coculture, the recovery of 3H thymidine labeled target cells was evaluated. The percent lysed target cells ± SD of triplicates is shown; significant differences in lysis by MDSC-Exo treatment are indicated by *. **(D)** LNC of untreated and MDSC-Exo-treated AA mice were cultured for 2 days in the presence of 10 U/ml IL2. Apoptosis was evaluated by flow-cytometry after AnnV-APC/PI staining. The % AnnV and AnnV/PI stained cells ± SD of triplicates is shown; significant differences in LNC apoptosis between untreated and MDSC-Exo-treated AA mice are indicated by *. **(E–G)** SC of untreated and MDSC-Exo-treated AA mice were excised, mRNA was isolated and subjected to deep sequencing. **(E)** mRNA up- or downregulated in SC after treatment of AA mice with MDSC-Exo were clustered according to molecular function (Panther Gene Analysis); **(F,G)** RNA engaged in adaptive and non-adaptive immune system activation or the communication between lymphoid and non-lymphoid cells that becomes **(F)** upregulated or **(G)** downregulated by MDSC-Exo treatment (Reactome). Abbreviations: IS, immune system; IR, immune response. Treatment of AA mice with MDSC-Exo promotes a shift toward immunosuppressive CD152 accessory molecule, an expansion of Treg and MDSC and pronounced immunosuppressive IL10 expression, but reduced inflammatory IL1β and IL6 expression. MDSC-Exo suppress helper T cells proliferation and cytotoxic T cells activity and promote lymphocyte apoptosis. mRNA analysis of SC from MDSC-Exo-treated AA mice confirmed strong upregulation of FoxP3 and arginase1, as well as a shift toward activation of innate immune responses and several molecules suppressing adaptive immune system activation. In line with this finding, several adaptive immunity-supporting mRNA were downregulated in SC of MDSC-Exo-treated AA mice.

The overall distribution of CD4+, CD8+, and NK cells was not altered after 6 weeks MDSC-Exo treatment. However, there was a slight increase in CD25+ and a strong increase in CD152+ and FoxP3+ cells. The latter corresponded to a significant increase of Treg in draining LNC and SkIL. In addition, MDSC became enriched in LNC and SkIL. Notably, too, there was a slight decrease in inflammatory IL1β and IL6 and a significant increase in IL10 expressing draining LNC. IFNγ expression became significantly reduced (Figure 7A). The proliferative response of LNC of MDSC-Exo-treated AA mice was reduced, the antigen-specific response being more strongly affected (Figure 7B). Cytotoxic activity of LNC in response to DC-AApept was also strongly impaired in MDSC-Exo-treated mice (Figure 7C). Finally, a higher number of apoptotic cells was seen in MDSC-Exo treated LN after *in vitro* restimulation for 2 days (Figure 7D). These findings suggest that repeated MDSC-Exo application affected AA-specific TH and CTL and promoted Treg expansion *in vivo*.

To sustain our interpretation, the spleen of mice with AA totalis repeatedly receiving i.v. injections of MDSC-Exo or NaCl as control was excised and mRNA was prepared immediately. Deep sequencing of SC mRNA from untreated and MDSC-Exo-treated AA mice confirmed the strong *in vivo* impact of MDSC-Exo. Panther analysis according to molecular functions indicated an overrepresentation mainly of receptor molecules in SC of MDSC-Exo-treated AA mice (Figure 7E). Reactome clustering of mRNA upregulated or downregulated in SC of MDSC-Exo-treated AA mice indicated an abundance of molecules engaged in transport, including vesicle transport, oxidation/reduction processes, and immunoregulation (Table S7 in Supplementary Material). Reactome analysis of the latter group showed strong changes in cytokine/chemokine and cytokine/chemokine receptor expression, C’ components, and proteases. Accordingly, cytokines, GPCR, TLR, FcR, and C’ signaling cascades were affected most frequently. This accounted for the impact on innate immune cells and their interaction with lymphoid cells. In the adaptive immune system, strong upregulation of FoxP3 and Arg1 need to be first mentioned. Furthermore, there was an abundance of molecules that inhibit B cell or T cell activation (Table S7A in Supplementary Material; Figure 7F). Fittingly, mRNA downregulated in the spleen of MDSC-Exo-treated AA mice revealed a dominance of molecules that balance overshooting immune reactivity, like Epx, and Errfi1 that interfere with inflammatory cytokine secretion or Dapk2 contributing to caspase activation and Fasl, which inhibits Casp8 activation and others that would promote T cell proliferation and activation (Hells, Lgals1, Nedd4, Thy1, Usp46, Zbtb32) (Table S7B in Supplementary Material; Figure 7G).

These findings unraveled that MDSC-Exo mitigate AA T cell hyperreactivity, correct for apoptosis resistance, strongly promote Treg recovery, and affect the crosstalk of the native with the adaptive immune system. This is the first *ex vivo* demonstration of MDSC-Exo activity at the mRNA level in autoimmune disease. The effects being even stronger than after *in vitro* coculture appear promising for MDSC-Exo as a therapeutic.

**Figure 8:**
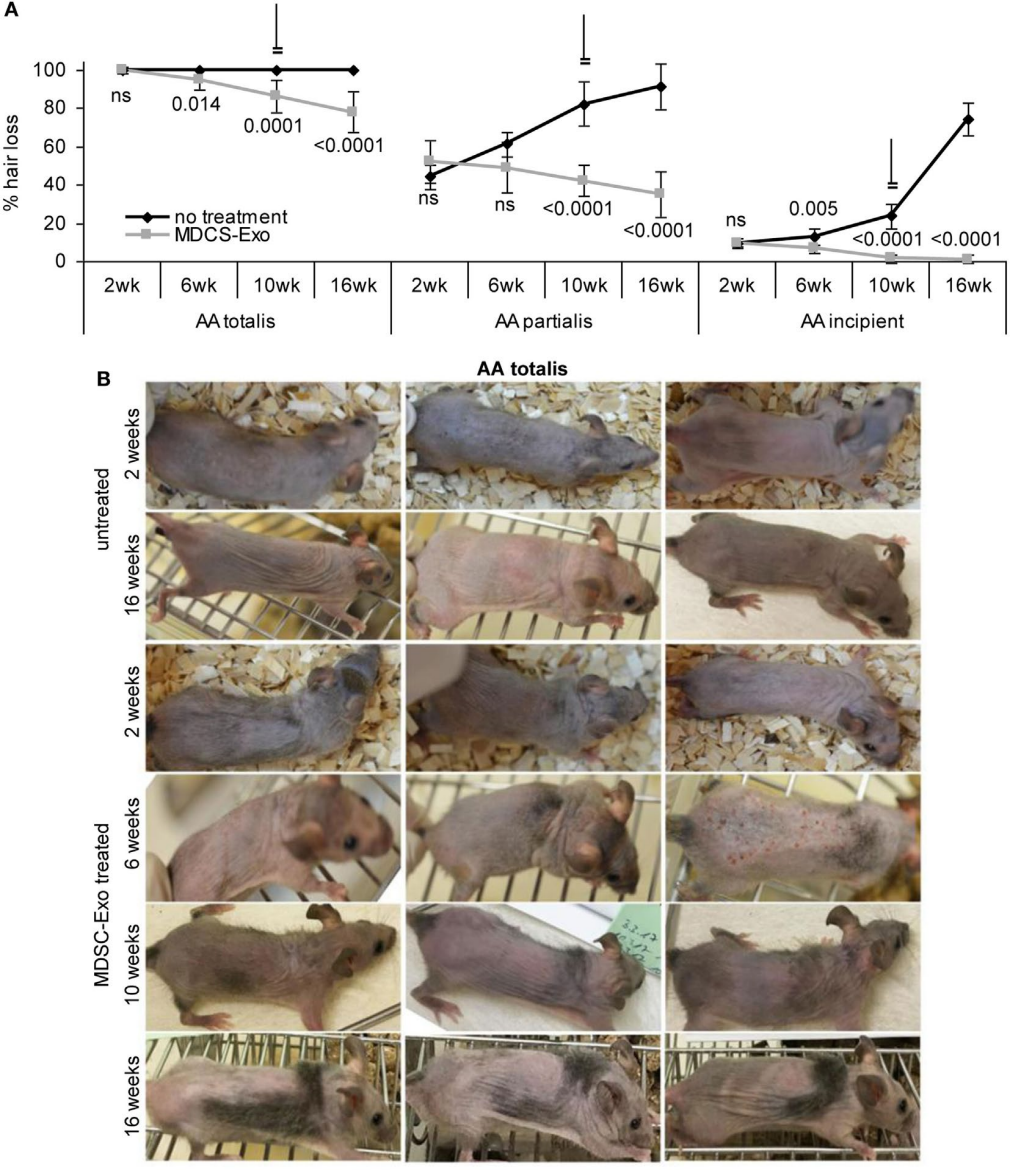
Hair growth in myeloid-derived suppressor cells (MDSC) exosome-treated alopecia areata (AA) mice. Ten mice per group with AA totalis, partialis, or incipient remained untreated or received (i.v.) 2×/week MDSC-Exo. Mice were treated for 10 weeks and controlled for hair growth for 16 weeks. **(A)** Hairless areas are documented in control and treated mice at 2, 6, and 10 weeks after treatment initiation as well as after 16 weeks (6 weeks after treatment termination). The mean hairless area ± SD of 10 mice/group is shown. Significant differences of treated versus untreated mice are indicated. **(B)** Three representative examples of the hair coat in control, and MDSC-Exo-treated mice with AA totalis after 2, 6, 10, and 16 weeks are shown. In incipient AA, treatment with MDSC-Exo is curative, in mice with AA totalis or partialis hair coat improves for ~30%. Though not curative, the therapeutic effect is long-lasting.

In mice (10/group) with AA totalis, partialis, and incipient the treatment was maintained for 10 weeks. Hair growth was controlled in 2-week intervals until 6 weeks after treatment termination. In mice with AA totalis, the impact of MDSC-Exo application became significant after 6 weeks of treatment and was highly significant after 10 and 16 weeks, i.e., was maintained and even slightly improved 6 weeks after treatment termination (Figure 8A). However, MDSC-Exo treatment was not curative for mice with AA totalis. Mostly thin hairs covered roughly 30% of the skin (Figure 8B).

Similar findings accounted for mice with AA partialis. Hair loss at the beginning varied between 50 and 30% (control group) and 70 and 35% (treatment group). In mice receiving MDSC-Exo, hair growth improved by 16% (hair cover range: 50–80%). Though after 6 weeks treatment the difference to the control group was not significant, it became highly significant after 10 and 16 weeks due to hair loss progression in the control group, which reached 91% (range: 65–100%) (Figure 8A; Figure S4A in Supplementary Material).

Myeloid-derived suppressor cells-Exo treatment was curative in mice with incipient AA. With a mean hair loss of 9% (range: 15–5 and 15–8%, respectively) in the control and treated group, hair loss was hardly visible in MDSC-Exo treated mice after 10 weeks treatment (hair coat: 95–100%). Hair growth remained stable after cessation of the therapy with a mean hair coat of 99% (range: 95–100%) after 16 weeks. With progressing hair loss in the control group, the impact of MDSC-Exo became significant already after 6 weeks and was highly significant after 10 and 16 weeks (Figure 8A; Figure S4B in Supplementary Material).

With a lasting curative effect in AA incipient, a clinical trial with MDCS-Exo appears worthwhile in this group of patients. The highly significant therapeutic impact of MDSC-Exo in AA totalis and partialis is encouraging for inclusion of MDSC-Exo in therapeutic settings. However, accompanying treatments of these groups of AA patients will be required.

## Discussion

Myeloid-derived suppressor cells are immature myeloid cells (70, 71), originally described to suppress immune responses in chronic infections (72). In cancer immunotherapy, too, MDSC are a major hindrance, hampering T cell recruitment and activation, while promoting M1 and Treg expansion (10, 73). On the other hand, MDSC are beneficial in overshooting immune reactions like autoimmune diseases (74) and allogeneic bone marrow transplantation (11), where the transfer of MDCS is a therapeutic option (75). As the logistics and costs for cell-based therapeutics are extremely high and their clinical application is frequently restricted to life threatening disease progression, Exo as the most efficient intercellular communicators (76) have come into focus. We here report on the characterization of MDSC-Exo in comparison to MDSC and explored the therapeutic use of MDSC-Exo in autoimmune disease using a mouse AA model.

### Comparison of *Ex Vivo* Isolated With *In Vitro* Generated MDSC and MDSC-Exo

Exosomes delivered by BMC culture-derived MDSC would greatly facilitate therapeutic application. MDSC-Exo could be stored. Provided MDSC do not differ significantly with the health status of the donor, large quantities of Exo could be collected from healthy volunteers that would circumvent collection-dependent variations. Furthermore, Exo are easy to manipulate *in vitro* (77, 78). Thus, their therapeutic potential could be fostered, where required. However, there is limited information on the comparability of MDSC-Exo and MDSC, which had to be clarified in advance. In addition, an answer was required on the impact of the originating organ and the health status of the donor.

We started comparing MDSC generated from the BM with MDSC isolated *ex vivo* by sorting for CD11b+Gr1+ cells. BMC-MDSC contained slightly fewer CD11b+ Gr1+ cells than the sorted cells. Also, TGFß expressing cells were slightly reduced that might not be relevant, as according to proteome analysis expression of TGFβ1-induced proteins is not affected. Inflammatory cytokines and immunosuppressive IL10 were comparable in the two MDSC populations. Chemokine receptor CCR5, CXCR3, and CXCR4 was reduced. Though CCR5 can support MDSC expansion and activity (79), reduced recovery might be balanced by higher CCR6 expression in BMC-MDSC (80). Importantly, a proteome analysis pointed toward higher Arg1 recovery and no deficits in proteins engaged in innate immune responses including inflammatory cytokine in BMC-MDSC. These findings argue for BMC-MDSC not being disadvantageous compared to *ex vivo* isolated MDSC.

The analysis was repeated with BMC-MDSC and *ex vivo* isolated CD11b+ Gr1+ cells from mice with a chronic inflammation induced by repeated treatment with the contact sensitizer SADBE. Both MDSC populations showed an increase in inflammatory cytokine and, less pronounced, CCR7 expression. Though this inflammation-induced difference in MDSC has to be kept in mind, differences between *ex vivo* isolated versus BMC-MDSC did not argue against the latter as Exo donor. Thus, we proceeded with a proteome analysis to manifest the suitability of BMC-MDSC for Exo collection.

Myeloid-derived suppressor cells-Exo abundantly express constitutive Exo membrane molecules (tetraspanins, TSG101, Alix, Annexins). Cytoplasmic molecules that are engaged in Exo biogenesis were also richly recovered. These include tetraspanin-associated molecules, components of the AP2 complex, the sorting complex, and rab proteins, which contribute to scission and fission of invaginated membrane domains leading to EE formation, the transport of EE to MVB, the transport of MVB to the cell membrane, and the exocytosis of the ILV (81). In addition, C’ (66, 82, 83) and proteasome components (84–86) were enriched in MDSC-Exo compared to MDSC. Enriched recovery of these proteins in Exo compared to donor cells is known and is not restricted to MDSC-Exo. This also accounts for the high recovery of GPCR, which are prone for internalization and integration into Exo during biogenesis (87, 88). The abundance of MDSC-Exo membrane-attached signaling molecules may additionally contribute to target cell reprogramming. On the other hand, TGFβ and IL1β expression was lower in MDSC-Exo than in MDSC. Further differences that might affect MDSC-Exo activity compared to MDSC are the lower recovery of Arg1, SOD2 and, possibly, BAX. Nonetheless, taking into account the limited number of proteins in MDSC-Exo (537) compared to MDSC (1008), only few MDSC-related proteins were underrepresented in MDSC-Exo and from these only three (Arg1, Sod2, and Bax) were possibly function-relevant. Thus, we speculated that reduction and abundance of proteins in MDSC-Exo versus MDSC might be balanced in concern about functional activity and that MDSC-Exo should have an advantage in targeting.

### MDSC Exo Targets and *In Vivo* Homing

The mode of Exo connected with potential targets as well as the mode of target selection is still disputed. Exo may bind *via* annexins, specific receptors, or fuse with the cell membrane (89). We provided evidence that protein complexes, particularly tetraspanin webs are a preferred unit for targeting, which binds to protein complexes on the targeted cell that could be located in membrane microdomains predetermined for internalization (62). Indeed, tetraspanins are constitutively enriched in Exo membranes due to their support in vesicle biogenesis (27, 90). Also, tetraspanin-associated molecules and components of the antigen-presenting complex on the one hand and components of the neuronal and the immune synapse on the other hand were reported to account for Exo binding (91–93), protein complexes rather than individual molecules facilitating binding stability.

In line with our finding that anti-CD81 most efficiently hampers MDSC-Exo uptake by LNC (94, 95), CD81 also is engaged in DC-Exo targeting the TCR synapse (96, 97). We are not aware of reports on the engagement of Ly6C and CCR6 in Exo binding. It could be due to the location of GPI-anchored molecules and GPCR in membrane domains that lipid composition supports invagination. T cells are DC-Exo targets (34). We here presented evidence for an engagement of synapse-incorporated CD4, CD8, and accessory molecules also in the MDSC-Exo—target interaction. Whether this also accounts for Treg, which express CD4 and CD25, both anti-CD4 and anti-CD25 inhibiting MDSC-Exo uptake, or whether Treg express additional ligands for MDSC-Exo receptors remains to be explored. The latter is likely, as Treg do not preferentially take up DC-Exo (unpublished). In B cells, and possibly NK, Mϕ, granulocytes, and mast cells FcRs may serve as targets (data not shown). Finally, we want to point out that there is a striking overlap of targets that take up MDSC-Exo and MDSC trogocytosis. This accounts for LNC of naïve mice and skin-draining LNC of AA mice, including preferential trogocytosis/uptake by activated T cells.

Myeloid-derived suppressor cells-Exo are also furnished for selective homing. Selective homing was described for tumor-Exo (98) as well as for embryonic and mesenchymal stem cell Exo (99). Thus, we expected enrichment in skin-draining LNC in AA. The striking recovery of MDSC-Exo in SkIL located in the vicinity of remnant hair follicles demands for further exploring engaged chemokine receptors and adhesion molecules, high CCR6 and CXCR4 expression might contribute. The target structures on SkIL remain to be defined.

Despite some open questions, the data demonstrate highly efficient MDSC-Exo uptake by activated T cells and Treg and preferential MDSC-Exo homing into skin-draining LN and the skin of AA mice.

### MDSC-Exo Affect Target Cell Activity

Myeloid-derived suppressor cells-Exo home, are taken up and are equipped to affect target cell activity. This demanded exploring target cell modulation *in vitro* and *ex vivo*.

Culturing lymphocytes obtained from naïve and AA mice in the presence of MDSC-Exo revealed a reduction in T cells expressing activation markers and in CD314 expressing NK cells. The number of CD152 expressing T cells and of Treg as well as the number of MDSC increased. Expression of inflammatory cytokines was increased in naïve and AA, IL10 expression was increased only in AA mice. The impact on TCR and TLR signaling cascades was minor. The impact on skin-draining LNC and SkIL was stronger in mice that were treated for 6 weeks with MDSC-Exo. This accounted particularly for the expansion of CD152+ and IL10 expressing LNC and Treg. On the other hand, the persisting exposure to MDSC-Exo was accompanied by a reduction in inflammatory cytokines and IFNγ.

mRNA sequencing of AA SC from untreated and repeatedly MDSC-Exo-treated mice showed an unexpectedly high number of mRNA that expression was changed significantly. mRNA increased in MDSC-Exo-treated AA mice were frequently engaged in transport, including Exo biogenesis, (co)transcription, and metabolism. When searching by the Reactome program exclusively for changes that affect the immune system, the strong upregulation of FoxP3 and arginase 1 should be mentioned first. Upregulation of the DC inhibitory Clec receptors may account for impaired antigen presentation and reduced proliferative activity (100, 101). High immunoregulatory cytokine and receptor expression could support reduced T cells activation. Whether the abundant chemokines and chemokine receptor expression supports or mitigates T cell and NK cell activity remains to be explored. This also accounts for the richness in proteases and their possible impact on apoptosis susceptibility. Similar concerns account for mRNA with reduced expression in SC of MDSC-Exo-treated AA mice. To name a few, reduced recovery of Camp (cationic antimicrobial peptides) (102), CD55 and Cfb (103), Chil1 (104), Clu (clusterin) (105), F2r (coagulation factor 2) (106), Fcer1a (107), Klrk1 (108), CXCL7 (109), S100a9 (110), CD244 (111), CTSG (112), Ms4a2 (membrane spanning 4-domains A2) (113), and VCAM1 (114) may contribute to impaired activity of the innate immune system and concomitantly dampen adaptive immune system responses. The impact of reduced CD34, Angp1, and Serpinb9 expression remains to be explored. Instead, reduced GZMB, GZMC, PRF1 (115), FasL (116), Rab27a (117), and TNFSF10 (TRAIL) (118) expression can account for mitigated CTL activity. Impaired IFNγ secretion, IL2R and IL12R expression may hamper overshooting reactivity of the adaptive immune system (119). B cell activation could become impeded by reduced adhesion G protein coupled receptor G3 (Adgrg3) (120), CKLF like MARVEL transmembrane domain containing 7 (CMTM7) (121), and Myb expression (122).

Taken together, the abundant as well as the reduced mRNA recovery in MDSC-Exo-treated AA SC documents the *in vivo* efficacy in dampening overshooting autoreactive T and, less pronounced NK activity as well as in supporting Treg expansion and MDSC activation.

The deep sequencing results were confirmed by impaired proliferation and cytotoxic activity and slightly reduced apoptosis resistance of LNC cocultured with MDSC-Exo and of LNC and SkIL of repeatedly MDSC-Exo-treated AA mice. First, it should be mentioned that the *in vitro* coculture of LNC with MDSC-Exo was concomitantly performed with MDSC and included LNC of naïve and AA mice. The impact of MDSC-Exo on LNC and SkIL proliferation and cytotoxicity resembled that of MDSC, but was slightly weaker. As MDSC/MDSC-Exo are supposed to affect NK and T cells, IL2 and AA-peptide-loaded DC were used as stimulus. Inhibition of proliferation and cytotoxicity was significantly stronger in AA lymphocytes stimulated with DC-AApept. However, as apparent by evaluating inhibition of leukocyte subpopulations, particularly MDSC-Exo inhibited NK proliferation poorly. This implies that MDSC/MDSC-Exo act preferentially on activated T cells. The poor response and insignificant suppression of CTL from naïve mice likely relies on AA-peptide-loaded DC being too weak a stimulus to induce CTL activation. Thus, as discussed for MDSC (6, 12), the seeming antigen-specificity of MDSC-Exo is due to their preferential uptake by activated T cells. This also accounts for the draining LNC of AA mice treated for 6 weeks with MDSC-Exo. The “AA-specific” T cells preferentially proliferate and display increased cytotoxic potential when restimulated with the nominal antigen. These activated T cells are more efficiently attacked by MDSC-Exo.

In brief, MDSC-Exo promote Treg expansion *in vitro* and more pronounced *in vivo*. They suppress the proliferative and cytotoxic response of activated T cells and slightly interfere with the relative apoptosis-resistance of activated T cells. MDSC-Exo additionally exert a strong impact on cytokine, chemokine, and receptor expression and may affect DC and antigen presentation. These latter aspects deserve further experimentations, as an impact of MDSC-Exo on antigen presentation could contribute to their therapeutic efficacy.

### Approaching AA Therapy With MDSC Exo

Myeloid-derived suppressor cells can replace a long-term maintained Th1-mediated DTH reaction induced by SADBE, where hair regrowth reached around 70% (60). MDSC-Exo sufficed for about 30% hair regrowth in AA totalis and partialis. The difference to untreated mice becoming more striking in the latter group due to hair loss progression in the absence of MDSC-Exo treatment. This also accounted for the treatment of mice with AA incipient, which was waved by MDSC-Exo. Notably, the therapeutic impact remained stable for 6 weeks after termination. This is due to MDSC-Exo affecting leukocyte proliferation, cytotoxic activity, and apoptosis resistance. According to their uptake, MDSC-Exo could directly affect activated T cells. Based on the strong expansion of Treg and the increase in IL10, we suggest a dominating contribution of MDSC-Exo in reestablishing peripheral tolerance.

## Conclusion and Outlook

Myeloid-derived suppressor cells are considered a therapeutic in autoimmune diseases and allogeneic BMC transplantation (7, 11). A comprehensive characterization of the protein profile of MDSC-Exo fortifies their potential activity in immune response regulation in autoimmune disease, explored in a mouse AA model. MDSC-Exo preferentially target activated T cells and strongly support Treg expansion *in vitro* and *in vivo*. Although their therapeutic efficacy remained slightly below that of a chronic DTH reaction or of transferred MDSC, efficacy can become uplifted by Exo transfection with few immunosuppression-relevant components that are under-represented in MDSC-Exo.

Based on the profound knowledge of the MDSC-Exo protein content and their impact on elements of the immune system *in vivo*, MDSC-Exo may become a leading therapeutics in autoimmune diseases in which progression relies on deficits in immunoregulation. As we worked in a syngeneic mouse model, possible advantages/disadvantages of an allogeneic system remain to be explored. Irrespective of this open question, the ease of generating, storing, and tailoring Exo from healthy volunteers facilitates their therapeutic application (77, 78). Last, but not least, compared to cell therapy, safety requirements should be easier to fulfill. Taken that patients receive intravenous injections of small vesicles in a physiological salt solution that are derived from healthy donors, side effects, or health risks are not be expected.

## Ethics Statement

Animal experimentations were approved by the local governmental authorities of Baden Wuerttemberg, Germany.

## Author Contributions

MZ, KZ, NK, and NB performed and analyzed experiments. MS performed the proteome and JP the deep sequencing analysis. MZ planned and analyzed experiments and wrote the manuscript, which was corrected by TH and MS and approved by all authors.

## Conflict of Interest Statement

The authors declare that the research was conducted in the absence of any commercial or financial relationships that could be construed as a potential conflict of interest.

## Abbreviations

AA, alopecia areata; BMC, bone marrow cells; C’, complement; CFSE, carboxyfluorescein-succinimidylester; CTL, cytotoxic T cells; DC, dendritic cells; DC-AApept, AA peptide-loaded DC; DTH, delayed-type hypersensitivity; Exo, exosomes; MDSC, myeloid-derived suppressor cells; MVB, multivesicular bodies; LNC, lymph node cells; PBL, peripheral blood leukocytes; PEC, peritoneal exudate cells; SADBE, squaric acid dibutylester; SC, spleen cells; SkIL, skin-infiltrating leukocytes; Treg, regulatory T cells; TH, helper T cells.
