# Erratum: Immunoregulatory Effects of Myeloid-Derived Suppressor Cell Exosomes in Mouse Model of Autoimmune Alopecia Areata

**DOI:** 10.3389/fimmu.2021.832980

**Published:** 2021-12-24

**Authors:** Frontiers Production Office

**Affiliations:** Frontiers Media SA, Lausanne, Switzerland

**Keywords:** myeloid-derived suppressor cells, exosomes, alopecia areata, therapy, regulatory T cells

Due to a production error, an author name was incorrectly spelled as “Natalia Kutlu”. The correct spelling is “N. Natali Kutlu”. The publisher apologizes for this mistake.

The original version of this article has been updated.

