# Supplementary material for: Immunoregulatory Effects of Myeloid-Derived Suppressor Cell Exosomes in Mouse Model of Autoimmune Alopecia Areata
Source: Front Immunol. 2018 Jun 6;9:1279. doi: 10.3389/fimmu.2018.01279 (PMC6008552; doi:10.3389/fimmu.2018.01279)
Supplement: Supplementary file 1 [file Presentation_1.PDF]

Supplement

Myeloid-derived suppressor cell exosomes have significant immunoregulatory effects in autoimmune disease as exemplified in mouse alopecia areata

Margot Zöller[1], Kun Zhao[1], Natalia Kutlu[1], Natalie Bauer[1], Jan Provaznik[2], Thilo Hackert[3], Martina Schnölzer[4]

[1] Tumor Cell Biology, University Hospital of Surgery, Heidelberg, [2] Gene Core Unit, EMBL Heidelberg, [3] Pancreas Section, University Hospital of Surgery, Heidelberg, [4] Functional Proteome Analysis, German Cancer Research Center, Heidelberg, Germany

Table S1	Antibodies and reagents
Table S2	Immunogenic Ha1 and K6irs keratinocyte peptides
Table S3	Comparison between *in vitro* versus *ex vivo* generated MDSC
S3A	Top score recovered peptides
S3B	Higher peptide recovery in *in vitro* than *ex vivo* MDSC
S3C	Higher peptide recovery in *ex vivo* than *in vitro* MDSC
Table S4	Protein profile of MDSC-Exo from *in vitro* / *ex vivo* MDSC
S4A	Top score recovered peptides
S4B	Higher peptide recovery in Exosomes from *in vitro* MDSC
S4C	Higher peptide recovery in Exosomes from *ex vivo* MDSC
Table S5	Differences in MDSC versus MDCS-Exo protein profile
S5A	Preferential peptide recovery in MDSC-Exo
S5B	Higher peptide recovery in MDSC than MDSC-Exo
Table S6	MDSC-Exo peptides recovered in pulldown from Sepharose-coupled lymph node cell membranes
Table S7	*In vivo* impact of MDSC-Exo on AA spleen cells
S7A	Increased mRNA recovery in spleen cells of MDSC-Exo treated AA mice
S7B	Reduced mRNA recovery in spleen cells of MDSC-Exo treated AA mice
Figure S1	Proteome analysis of *ex vivo* and *in vitro* generated MDCS and MDSC-exosomes
Figure S2	Proteome analysis comparing *ex vivo* and *in vitro* generated MDCS with MDSC-exosomes
Figure S3	MDSC-exosome membrane attached proteins
Figure S4	Examples of hair regrowth in MDSC exosome-treated mice with AA partialis and incipient

Table S1
Antibodies and reagents

**Antibody specificity**	**donor**	**supplier**
Akt	mouse	Becton Dickinson, HD, G
(p)Akt	mouse	Becton Dickinson, HD, G
Alix	rabbit	Santa Cruz, HD, G
BAD	hamster	Becton Dickinson, HD, G
pBAD	rabbit	Cell Signaling, Frankfurt, G
BclXl	rabbit	Cell Signaling, Frankfurt, G
Caspase 3 activ	rabbit	Becton Dickinson, HD, G
Caspase 8	rabbit	Becton Dickinson, HD, G
Caspase9-clv.	rabbit	Cell Signaling, Frankfurt, G
CCR3 (CD193)	rat	R&D Systems, G
CCR4 (CD194)	goat	abcam, Cambridge, UK
CCR5 (CD195)	rat	Becton Dickinson, HD, G
CCR6 (CD196)	rat	R&D Systems, G
CCR7 (CD197)	rat	R&D Systems, G
CCR8 (CDw198)	goat	abcam, Cambridge, UK
CCR9 (CDw199)	rat	R&D Systems, G
CD3ζ	rabbit	Abcam, Cambridge, UK
CD4	rat	EACC[b]
CD8	rat	EACC[b]
CD9	rat	Becton Dickinson, HD, G
CD11b (ITαM) (YBM6.6.10)	rat	EACC[b]
CD11c (ItgαX)	hamster	Becton Dickinson, HD, G
CD14	rat	Becton Dickinson, HD, G
CD16 (FcRIIIb)	rabbit	abcam, Cambridge, UK
CD16/32 (NK)	rat	Becton Dickinson, HD, G
CD19	rat	ImmunoTools, Friesoythe, G
CD25 (PC61)	rat	EACC[b]
CD28	monocl.hamster	Becton Dickinson, HD, G
CD32 (2.4G2)	rat	ATCC[c]
CD40	rat	Becton Dickinson, HD, G
CD47 (IAP)	rat	Becton Dickinson, HD, G
CD54 (ICAM1)	monocl.hamster	Becton Dickinson, HD, G
CD63 (Tspan30)	rabbit	Santa Cruz, HD, G
CD69	monocl.hamster	ImmunoTools, Friesoythe, G
CD80	rat	Becton Dickinson, HD, G
CD81 (Tspan28)	monocl.hamster	ImmunoTools, Friesoythe, G
CD86	rat	Becton Dickinson, HD, G
CD103 (ITαE)	rat	Becton Dickinson, HD, G
CD115 (M-CSFR)	rabbit	abcam, Cambridge, UK
CD117 (c-KIT)	rat	Becton Dickinson, HD, G
CD135 (FLT3)	rat	Becton Dickinson, HD, G
CD152	monocl.hamster	Becton Dickinson, HD, G
CD154	monocl.hamster	Becton Dickinson, HD, G
CD314 (NKG2D)	rat	Becton Dickinson, HD, G
CXCR3 (CD183)	rat/rabbit	Santa Cruz, HD, G
CXCR4 (CD184)	rat	Becton Dickinson, HD, G
Erk1/2	mouse	Becton Dickinson, HD, G
ERK1/2-p	mouse	Becton Dickinson, HD, G
F4/80	rat	Becton Dickinson, HD, G
FoxP3	rat	Becton Dickinson, HD, G
GM-CSF	rat	Becton Dickinson, HD, G
Gr1	rat	ImmunoTools, Friesoythe, G
HIF1α	mouse	Novus Biologicals, Littletown, USA
IFNγ	rat	Becton Dickinson, HD, G
IκB	rabbit	Santa Cruz, HD, G
IκB-p	mouse	Becton Dickinson, HD, G
IgM	rabbit	Dianova, Hamburg, G
IL1β	rat	BioTrend, Cologne, G
IL4	rat	Becton Dickinson, HD, G
IL6	rat	Becton Dickinson, HD, G
IL10	rat	Becton Dickinson, HD, G
IL12	rat	Becton Dickinson, HD, G
JAK2	rabbit	SantaCruz, HD, G
JAK2-p	rabbit	SantaCruz, HD, G
JAK3	mouse	Becton Dickinson, HD, G
JAK3-p	goat	SantaCruz, HD, G
JNK	rabbit	SantaCruz, HD, G
pJNK	mouse	Becton Dickinson, HD, G
Jun	rabbit	SantaCruz, HD, G
Jun-p	rabbit	SantaCruz, HD, G
Ly6C	rat	Becton Dickinson, HD, G
Ly6G	rat	Becton Dickinson, HD, G

Table S1 continued

**Antibody specificity**	**donor**	**supplier**
MHCI (K7-65)	mouse	Ref 1[d]
MHCII (K25-137)	mouse	Ref 1[d]
Myc-p	rabbit	Santa Cruz, HD, G
MyD88	rabbit	Santa Cruz, HD, G
NFκB	rabbit	Cell Signaling, Franfurt, G
NK (PK136)	rat	ATCC
NOS2	rabbit	SantaCruz, HD, G
P38	rabbit	SantaCruz, HD, G
P38-p	rabbit	SantaCruz, HD, G
PI3K	rabbit	Cell Signaling, Frankfurt, G
pPI3k	rabbit	Cell Signaling, Frankfurt, G
Rab5	mouse	Becton Dickinson, HD, G
SCA1 (Ly6A)	rat	Becton Dickinson, HD, G
SCF (KIT ligand)	rabbit	abcam, Cambridge, UK
Shc	rabbit	Cell Signaling, Franfurt, G
Shc-p	rabbit	Cell Signaling, Franfurt, G
Src	rabbit	Cell Signaling, Franfurt, G
Src-p	rabbit	Cell Signaling, Franfurt, G
STAT3-p	rabbit	Cell Signaling, Franfurt, G
STAT4-p	mouse	Becton Dickinson, HD, G
STAT6-p	rabbit	SantaCruz, HD, G
Survivin	rabbit	SantaCruz, HD, G
Ter119	rat	Becton Dickinson, HD, G
TGFβ1	rat	Becton Dickinson, HD, G
TLR2 (CD282)	rat	Becton Dickinson, HD, G
TLR3	rabbit	SantaCruz, HD, G
TLR4 (CD284)	rat	Becton Dickinson, HD, G
TNFα	rat	Becton Dickinson, HD, G
TSG101	rabbit	SantaCruz, HD, G
ZAP70	rabbit	Cell Signaling, Frankfurt, G
ZAP70-p	rabbit	Cell Signaling, Frankfurt, G
dye or biotin labeled secondary antibodies /Streptavidin		Dianova, Becton Dickinson, Amersham

**Reagent**	**Dose**	**Supplier**
AnnV-FITC / APC	0.2µl-0.5µl	Becton Dickinson, HD, G
CFSE (carboxyfluorescein-succinimidylester)	5µM	Invitrogen, Darmstadt, G
GM-CSF	(10-20ng/ml	ImmunoTools, Friesoythe, G
IL4	2ng/ml	ImmunoTools, Friesoythe, G
IL6	5ng/ml	ImmunoTools, Friesoythe, G
PGE2	1nM	BioTrend, Cologne, G
PI	0.08µg	Becton Dickinson, HD, G
Sulfobiotin-X-NHS	1mg/ml	Calbiochem, Darmstadt, G

[a] all rat and mouse antibodies are monoclonal, monoclonal hamster antibodies are marked
[b] EACC: European Collection of Animal Cell Cultures
[c] ATCC: American Type Cell Culture Collection
[d] Reference

1. Koch N, Hämmerling GJ, Tada N, Kimura S, Hämmerling U. Cross-blocking studies with monoclonal antibodies against I-A molecules of haplotypes b, d and k. Eur J Immunol (1982) 12: 909-14.

Table S2
Immunogenic Ha1 and K6irs keratinocyte peptides

**MHCI**		**MHCII**	
**Name-Position**	**Sequenz**	**Name-Position**	**Sequenz**
Ha1(K31)-147	T**E**LGLRQ**L**V	Ha1-204	**Q**LG**D**R**L**NVEVDAAPT
Ha1(K31)-211	V**E**VDAAP**T**V	Ha1-223	**R**VL**N**E**T**RCQYEAMVE
Ha1-157	S**D**INGLR**R**I	Ha1-249	**T**QT**E**E**L**NKQVVSSSE
Ha1-251	T**E**ELNKQ**V**V	Ha1-277	**R**TV**N**A**L**EIELQAQHC
K6irs(K71)-205	S**E**LRNVR**D**V	K6irs-296	**L**DL**D**S**I**IDEVRAQYE
K6irs-178	L**E**PILEG**H**I	K6irs-118	**V**EL**D**P**E**IQKVRAQER
K6irs-193	L**E**TLSGD**R**V	K6irs-382	**Q**RG**D**S**A**LKDARAKLD
K6irs-242	V**D**AAYAN**K**V	K6irs-289	**S**MD**N**N**R**NLDLDSIID

Table S3

Comparison between *in vitro* versus *ex vivo* generated MDSC

Table S3A **Highest score recovered proteins in *ex vivo* and *in vitro* generated MDSC**

		MDSC *in vitro*			MDSC *ex vivo*		
**Synonym**	**Protein Description**	**Score[a]**	**Prot.M.**	**Pept.M.**	**Score[a]**	**Prot.M.**	**Pept.M.**
Aco2	Aconitate hydratase, mitochondrial	**1252**	**78**	**20**	**1315**	**119**	**32**
Actb	Actin, cytoplasmic 1	**1972**	**1165**	**47**	**1995**	**1063**	**48**
Actn1	actinin alpha 1	**2631**	**141**	**35**	**2453**	**116**	**31**
Actn4	actinin alpha 4	**2575**	**128**	**36**	**2151**	**96**	**29**
Aldh2	aldehyde dehydrogenase 2 family	**1551**	**262**	**29**	**1555**	**245**	**33**
Aldoa	aldolase, fructose-bisphosphate A	**1401**	**182**	**18**	**1206**	**131**	**14**
Anxa1	Annexin A1	**2043**	**134**	**24**	**1963**	**134**	**24**
Anxa2	Annexin A2	**1679**	**180**	**21**	**1359**	**160**	**17**
Anxa3	Annexin A3	**1474**	**43**	**18**	869	24	14
Anxa4	Annexin A4	**1464**	**50**	**17**	**1018**	**29**	**12**
Anxa5	Annexin A5	**1574**	**44**	**20**	963	31	13
Anxa6	Annexin A6	**1727**	**80**	**18**	**1215**	**38**	**15**
Atp1a1	ATPase Na+/K+ transporting subunit alpha 1	**1058**	**49**	**11**	**1314**	**105**	**18**
Atp2a2	ATPase Ca2+ transporting 2	**1234**	**72**	**15**	**2257**	**155**	**26**
Atp5a1	ATP synthase, H+ transporting, complex alpha 1	**2090**	**331**	**25**	**2025**	**321**	**25**
Atp5b	ATP synthase, H+ transporting complex, beta 5	**2222**	**296**	**25**	**2205**	**301**	**25**
Atp6v1a	ATPase H+ transporting V1 subunit A	**1115**	**52**	**16**	**1807**	**104**	**24**
Calr	Calreticulin	**1533**	**159**	**22**	**1460**	**143**	**21**
Canx	Calnexin	**1538**	**87**	**19**	**1581**	**121**	**22**
Cap1	Adenylyl cyclase-associated protein 1	**1317**	**170**	**17**	**1179**	**76**	**16**
Capn1	Calpain-1	**1186**	**29**	**18**	759	21	11
Cat	Catalase	**1101**	**30**	**15**	**1347**	**58**	**21**
CD11b	Integrin alpha-M	**2995**	**244**	**38**	**2569**	**268**	**33**
CD13	Alanyl aminopeptidase, membrane	**1972**	**65**	**24**	**1874**	**67**	**23**
CD177	CD177 antigen Cd177	*1019*	*39*	*13*	**1283**	**43**	**18**
CD18	Integrin beta-2	**3098**	**178**	**39**	**3099**	**259**	**37**
CD206	mannose receptor C-type 1	**2224**	**48**	**27**	**1580**	**41**	**20**
Ckap4	Cytoskeleton-associated protein 4	**1713**	**54**	**20**	**1668**	**60**	**21**
Cltc	Clathrin heavy chain 1	**3779**	**99**	**49**	**3425**	**107**	**42**
Cndp2	CNDP dipeptidase 2	**1218**	**77**	**16**	857	40	13
Copa	coatomer protein complex subunit alpha	**1159**	**23**	**15**	**1033**	**34**	**15**
Ctsb	Cathepsin B	**1139**	**66**	**14**	746	25	10
Cybb	Cytochrome b-245 beta chain	*787*	*50*	*13*	**1053**	**130**	**14**
Dync1h1	dynein cytoplasmic 1 heavy chain 1	**2332**	**56**	**36**	**3537**	**89**	**53**
Eef2	Elongation factor 2	**2630**	**269**	**35**	**1538**	**167**	**21**
Ehd4	EH domain-containing 4	884	45	12	**1276**	**57**	**18**
Eif4a1	Eukaryotic initiation factor 4A1	**1254**	**135**	**15**	**1141**	**117**	**14**
Eno1	enolase 1	**2380**	**373**	**27**	**2238**	**280**	**25**
Esyt1	Extended synaptotagmin-1	**2753**	**217**	**37**	**3012**	**261**	**44**
Ezr	Ezrin	**1565**	**123**	**21**	**1370**	**91**	**17**
Fasn	Fatty acid synthase	**1018**	**25**	**15**	**1200**	**22**	**16**
Fermt3	Fermitin family member 3	**1519**	**111**	**21**	**1160**	**126**	**16**
Flna	Filamin-A	**5389**	**391**	**71**	**4448**	**314**	**54**
Ganab	glucosidase II alpha subunit	**1962**	**113**	**26**	**2094**	**115**	**29**
Gapdh	Glyceraldehyde-3-phosphate dehydrogenase	**1357**	**401**	**13**	**1158**	**243**	**11**
Gdi2	GDP dissociation inhibitor 2	**1335**	**92**	**15**	**1147**	**65**	**16**
Glg1	Golgi apparatus protein 1	920	32	10	**1423**	**50**	**18**
Glud1	Glutamate dehydrogenase 1	**1589**	**77**	**21**	**1793**	**131**	**26**
Gnai2	G protein alpha inhibiting 2	**1310**	**140**	**15**	**1340**	**221**	**17**
Got2	Aspartate aminotransferase, mitochondrial	**1177**	**93**	**19**	**1064**	**80**	**18**
Gpi	Glucose-6-phosphate isomerase	**1495**	**113**	**21**	**1125**	**67**	**15**

Table S3A cont

Synonym	Protein Description	MDSC *in vitro* Score[a]	MDSC *in vitro* Prot.M.	MDSC *in vitro* Pept.M.	MDSC *ex vivo* Score[a]	MDSC *ex vivo* Prot.M.	MDSC *ex vivo* Pept.M.
Gsn	Gelsolin	1257	48	15	1044	50	12
Hbb-b1	hemoglobin, beta adult major chain	1295	503	14	960	184	12
Hbb-b2	hemoglobin, beta adult minor chain	1130	299	14	854	117	12
Hnrnpa2b1	Heterogeneous nuclear ribonucleoproteins A2/B1	1286	82	17	479	10	5
Hsd17b4	hydroxysteroid 17-beta dehydrogenase 4	464	9	5	1082	44	11
Hsp90aa1	Heat shock protein HSP 90-alpha	1900	255	27	1289	173	18
Hsp90ab1	Heat shock protein HSP 90-beta	2507	419	33	1973	309	27
Hsp90b1	heat shock protein 90, beta (Grp94), member 1	2758	457	35	3265	525	42
Hspa5	heat shock protein 5	3362	544	38	3586	610	38
Hspa8	Heat shock cognate 71 kDa protein	3021	436	38	2745	330	35
Hspd1	heat shock protein family D (Hsp60) member 1	1943	69	24	861	44	10
Hyou1	Hypoxia up-regulated protein 1	2463	169	30	2426	161	31
Idh2	Isocitrate dehydrogenase [NADP] 2	920	63	14	1081	77	18
Immt	inner membrane mitochondrial protein	435	7	5	1078	44	14
Iqgap1	IQ motif containing GTPase activating protein 1	3437	233	43	2981	239	36
Lcp1	lymphocyte cytosolic protein 1	3082	486	38	2690	400	37
LDHA	L-lactate dehydrogenase A chain	1329	147	16	1064	129	16
Lmna	Lamin-A/C Lmna				1994	102	25
Lmnb1	Lamin-B1 Lmnb1	693	21	10	2297	131	27
Lnpep	Leucyl-cystinyl aminopeptidase	1122	37	15	1132	34	15
Ltf	Lactotransferrin	3279	223	41	2903	192	37
Mdh2	Malate dehydrogenase 2	1365	114	16	1281	88	16
Mpo	Myeloperoxidase	1761	149	21	1271	68	17
Msn	Moesin	2728	221	36	2573	158	34
Mvp	Major vault protein	1750	45	24	1791	55	25
Myh9	myosin heavy chain 9	5150	207	69	263	3	2
Myof	Myoferlin	191	4	3	3636	186	52
Napa	NSF attachment protein alpha	655	16	8	1066	31	14
Ncl	Nucleolin	1729	87	23	816	51	12
Oat	Ornithine aminotransferase	711	16	9	1119	48	16
Ogdh	oxoglutarate dehydrogenase	1079	38	15	1201	68	20
P4hb	prolyl 4-hydroxylase subunit beta	2412	286	31	2351	312	31
Pdia3	Protein disulfide-isomerase A3	2747	263	37	2655	335	39
Pdia4	Protein disulfide-isomerase A4	2505	182	32	2519	215	35
Pdia6	Protein disulfide-isomerase A6	1286	113	15	1358	155	14
Pgk1	Phosphoglycerate kinase 1	1289	107	13	960	69	13
Pgls	6-phosphogluconolactonase	1191	156	15	1018	113	13
Pkm	Pyruvate kinase PKM	2580	403	31	2108	298	27
Plek	Pleckstrin	352	14	5	1959	53	25
Por	cytochrome P450 oxidoreductase	1104	67	14	1248	92	15
Ppib	Peptidyl-prolylisomerase B	1043	55	13	1130	60	14
Ptpn6	protein tyrosine phosphatase, non-receptor type 6	1348	110	17	1182	106	15
Ptprc	protein tyrosine phosphatase, receptor type C	1229	85	17	1247	113	20
Pygl	Glycogen phosphorylase L	1534	106	22	838	41	13
Rab7a	RAB7A, member RAS oncogene family	1229	149	15	1009	88	13
Rack1	Receptor of activated protein C kinase 1	1145	81	16	762	53	12
Rdx	Radixin	1183	108	17	1075	80	14
Rnh1	ribonuclease/angiogenin inhibitor 1	1332	52	14	1035	50	12
Rpn1	ribophorin I	1331	169	21	1783	250	23
Rpn2	ribophorin II	1212	95	15	1371	150	17
Rrbp1	Ribosome-binding protein 1	1444	46	19	1724	54	24
Sdha	succinate dehydrogenase complex subunit A	497	19	6	1089	66	13
Serpinb1a	Leukocyte elastase inhibitor A	1377	156	19	913	64	12

Table S3A cont.

Synonym	Protein Description	MDSC *in vitro* Score[a]	MDSC *in vitro* Prot.M.	MDSC *in vitro* Pept.M.	MDSC *ex vivo* Score[a]	MDSC *ex vivo* Prot.M.	MDSC *ex vivo* Pept.M.
Slc25a12	solute carrier family 25 member 12	634	40	10	**1297**	**89**	**17**
Slc25a4	solute carrier family 25 member 4	868	109	13	**1169**	**171**	**16**
Slc25a5	solute carrier family 25 member 5	**1193**	**204**	**15**	**1328**	**280**	**18**
Snd1	Staphylococcal nuclease and tudor domain-1	***1459***	***51***	***21***	*483*	*17*	*5*
Sun2	SUN domain-containing protein 2	**1682**	**125**	**20**	**1539**	**107**	**19**
Thbs1	Thrombospondin-1	***1206***	***64***	***16***	*268*	*10*	*3*
Tkt	Transketolase	**1676**	**163**	**20**	**1625**	**113**	**20**
Tln1	Talin-1	**4912**	**297**	**57**	**5199**	**342**	**61**
Tuba1b	Tubulin alpha-1B	**1440**	**238**	**19**	**1360**	**157**	**18**
Tuba1c	Tubulin alpha-1C	**1357**	**222**	**18**	**1295**	**137**	**17**
Tuba4a	Tubulin alpha-4A	**1352**	**189**	**18**	**1189**	**122**	**16**
Tubb2a	Tubulin beta-2A	***1173***	***166***	***15***			
Tubb4b	Tubulin beta-4B	**1454**	**203**	**17**	**1332**	**160**	**17**
Tubb5	Tubulin beta-5	**1628**	**247**	**20**	**1459**	**179**	**19**
Uba1	Ubiquitin-like modifier-activating enzyme 1	**2006**	**101**	**23**	**1243**	**67**	**14**
Uggt1	UDP-glucose glycoprotein glucosyltransferase 1	**3039**	**116**	**39**	**2906**	**124**	**38**
Vcp	valosin containing protein	**2350**	**132**	**29**	**1970**	**173**	**24**
Vdac1	Voltage-dependent anion channel 1	**1431**	**64**	**15**	**1545**	**73**	**16**
Vim	Vimentin	**3056**	**371**	**42**	**2495**	**153**	**37**

[a] Score ≥1000: **bold**, ≥2-fold difference in significant peptide hits: *italics*

Table S3B

Peptides recovered at a significantly higher frequency in *in vitro* than *ex vivo* MDSC

	MDSC *in vitro*		MDSC *ex vivo*				
**Synonym**	**Prot.M.**	**Pept.M.**	**Prot.M.**	**Pept.M.**	**Protein Description**	**Protein Family**	**Reactome pathway**
**Ap1b1**	**33**	**12**	**17**	**2**	**AP-1 complex subunit beta-1**	membrane traffic.	protein transporter & binding
**Ap1m1**	**5**	**4**			**AP-1 complex subunit mu-1**	ECM receptor	vesicle biogenesis & transport
**Ap2b1**	**23**	**6**			**AP-2 complex subunit beta**	membrane traffic., prot. transporter	vesicle med. transport, endocyt., signaling
**Arg1**	**35**	**10**			**Arginase-1**	cysteine protease	scavenger receptor
**Arsb**	**5**	**3**			**Arylsulfatase B**	Hydrolase	
**Ca1**	**6**	**3**			**Carbonic anhydrase 1**	dehyratase	metabolism
**Ca2**	**21**	**7**	**6**	**2**	**Carbonic anhydrase 2**	dehyratase	metabolism
**Cbr2**	**5**	**4**			**Carbonyl reductase 2**	dehydrogenase	reductase, protein binding
**CD41**	**23**	**12**			**Integrin alpha-IIb**	Adhesion	adhesion and signaling
**CD61**	**14**	**6**			**Integrin beta-3**	Adhesion	adhesion and signaling
**Celf2**	**10**	**5**			**CUGBP Elav-like family member 2**	nucleic acid & RNA binding	
**Chp1**	**5**	**3**			**calcineurin like EF-hand protein 1**	phosphatase	metabolism, hyaluronan degradation
**Cmpk2**	**37**	**12**			**cytidine/uridine monophosph kinase 2**	nucleotide kinase	
**Cpd**	**6**	**3**			**Carboxypeptidase D**	metalloprotease	vesicle biogenesis
**Ddx39a**	**80**	**14**	**4**	**4**	**DEAD-box helicase 39A**	RNA helicase	mRNA processing
**Ddx3x**	**17**	**7**			**DEAD-box helicase 3, X-linked**	GTPase activity	protein-, RNA-, DNA-binding
**Elavl1**	**13**	**6**			**ELAV like RNA binding protein 1**	protein & RNA & nucleic acid binding	splicing
**Ethe1**	**6**	**3**			**ETHE1, persulfide dioxygenase**	hydrolase	ion binding
**Fam3c**	**10**	**4**			**family with similarity 3 member C**		platelet activation & signaling
**Glo1**	**12**	**6**	**1**	**1**	**glyoxalase I**	lyase	citric acid cycle, electron transport
**Gmfg**	**13**	**6**	**2**	**1**	**Glia maturation factor gamma**	signaling	Arp2/3 complex binding
**Grb2**	**13**	**7**	**1**	**1**	**Growth factor receptor-bound prot. 2**	non-receptor tyrosine kinase	multiple signaling pathways
**Gstm1**	**26**	**12**	**1**	**1**	**Glutathione S-transferase Mu 1**	transferase	
**Gsto1**	**13**	**6**			**Glutathione S-transferase omega-1**	cytoskel., transferase, elong. factor	metabolism
**Hadh**	**7**	**4**			**hydroxyacyl-CoA dehydrogenase**	dehydrogenase	metabolism
**Hck**	**12**	**4**			**HCK protooncogene, Src family TK**	tyrosine kinase	protein kinase, transferase
**Hdac1**	**9**	**6**			**Histone deacetylase 1**	reductase	protein binding, transcription factor activity
**Hnrnpa1**	**95**	**15**	**2**	**1**	**Heterogeneous ribonucleoprotein A1**	RNA-, nucleic acid binding	signaling
**Hnrnpa2b1**	**82**	**17**	**10**	**5**	**Heterogeneous ribonucleoprot. A2/B1**	RNA-, miRNA binding	
**Hnrnph1**	**67**	**13**	**10**	**4**	**Heterogeneous ribonucleoprotein H1**	protein & RNA & nucleic acid binding	splicing and signaling
**Hnrnpl**	**31**	**11**	**5**	**3**	**Heterogeneous ribonucleoprotein L**	protein, mRNA binding	splicing
**Hprt1**	**9**	**5**			**Hypoxanthine phosphoribosyltransf. 1**	glycoyltransferase	metabolism
**Hsd17b10**	**6**	**3**			**hydroxysteroid 17-β dehydrogenase 10**	dehydrogenase, reductase	metabolism
**Ifi44**	**27**	**9**			**IFN-induced protein 44**		molecular function
**Ifit1**	**40**	**12**			**IFN-induc. prot. w tetratricopep.rep.1**	protein & RNA binding	

Table S3B cont.

	MDSC *in vitro*		MDSC *ex vivo*				
**Synonym**	**Prot.M.**	**Pept.M.**	**Prot.M.**	**Pept.M.**	**Protein Description**	**Protein Family**	**Reactome pathway**
Ifit2	19	6			IFN-induc. prot. w tetratricopep. rep. 2		RNA binding
Ifit3	20	4			IFN-induc. prot. W tetratricopep. rep. 3		protein binding
Ilf2	5	3			Interleukin enhancer-binding factor 2	kinase activator	
Iqgap2	4	3			IQ motif contain. GTPase active. prot.2	G-protein modulator	Rho GTPase signaling
Irgm1	7	3			Immunity-relat. GTPase family M prot.1	hydrolase	
Isg15	52	4			ISG15 ubiquitin-like modifier	protein binding	cytokine signaling
Kpnb1	55	13	20	4	karyopherin subunit beta 1	transfer / carrier protein	apoptosis execution
Mcm4	7	4			minichromosome mainten. complex 4	DNA helicase, hydrolase	cell cycle
Mcm7	13	3			minichromosome mainten. complex 7	hydrolase, DNA helicase	cell cycle
Mcpt8	11	4			Mast cell protease 8 Mcpt8	serine protease	proteolysis
Mrps34	5	4			mitochondrial ribosomal protein S34		protein synthesis, ribosome constituent
Msra	5	3			Methionine sulfoxide reductase 1	reductase	metabolism
Mta2	10	6			metastasis assoc. 1 family member 2	chromatin binding	DNA & protein binding
Mybbp1a	29	12	2	1	Myb-binding protein 1A	transcription factor	protein & RNA-binding
Myh9	207	69	3	2	myosin heavy chain 9	G-protein modul., actin binding	protein binding
Myl12b	27	8			myosin light chain 12B	binding	Rho GTPase signaling
Nipsnap3b	8	4			NipSnap homolog 3B		vesicular trafficking
Npc2	7	4	0	0	NPC intracell. cholesterol transporter 2	cholesterol binding	cholesterol transport
Nsf	7	4			Vesicle fusing ATPase	hydrolase	COPI-,COPII-mediated vesicle transport
Nudt21	13	7			nudix hydrolase 21	hydrolase	mRNA splicing
Ostf1	9	6			Osteoclast-stimulating factor 1	SH3 domain binding	bone resorption
Park7	9	4			Parkinsonism assoc. deglycase, DJ-1	protease, transcription	scaffold protein binding
Pcmt1	5	3			protein-L-isoaspartate methyltransfer.	methyltransferase	
Pddc1	8	3			Parkinson disease 7 domain-cont. 1	RNA binding, transcription factor	
Pds5a	6	3			PDS5 cohesin associated factor A	chromatin binding	cell cycle
Pf4	4	3			Platelet factor 4	chemokine	CXCR3 signaling
Pfdn2	6	4	1	1	Prefoldin subunit 2	chaperone	protein folding
Ppil1	4	3			peptidylprolyl isomerase like 1	isomerase	splicing
Ppp1ca	36	13	7	4	protein phosphatase 1 subunit alpha	phosphatase	GPCR signailng
Ppp1cb	19	9			protein phosphatase 1 subunit beta	phosphatase	Rho GTPase signaling, cell cycle
Ppp1cc	25	11			protein phosphatase 1 subunit gamma	phosphatase	protein binding, phosphatase activity
Ppp2ca	12	7	1	1	protein phosphatase 2 subunit alpha	phosphatase	TLR4 cascade, MAPK signaling,
Ppp2r1a	15	7	3	2	Protein phosphatase 2A subunit A	phosphatase	TLR & multiple signaling
Prdx6	30	13			Peroxiredoxin-6	peroxidase	peroxidase, hydrolase, binding
Psma2	14	8	1	1	Proteasome subunit alpha 2	endopeptidase	multiple signaling pathways
Psma3	14	6	1	1	Proteasome subunit alpha 3	peptidase, hydrolase	signaling
Psma5	19	8			Proteasome subunit alpha 5	peptidase, hydrolase	signaling

Table S3B cont.

	MDSC *in vitro*		MDSC *ex vivo*				
**Synonym**	**Prot.M.**	**Pept.M.**	**Prot.M.**	**Pept.M.**	**Protein Description**	**Protein Family**	**Reactome pathway**
**Psma6**	**18**	**8**	**3**	**2**	**Proteasome subunit alpha 6**	peptidase, hydrolase	signaling
**Psma7**	**17**	**10**			**Proteasome subunit alpha 7**	endopeptidase	multiple signaling pathways
**Psmb10**	**6**	**3**			**Proteasome subunit beta 10**	endopeptidase	multiple signaling pathways
**Psmb3**	**11**	**5**			**Proteasome subunit beta 3**	peptidase	
**Psmb4**	**20**	**7**			**Proteasome subunit beta 4**	peptidase	
**Psmb8**	**14**	**6**			**Proteasome subunit beta 8**	endopeptidase	GPCR signaling
**Psmb9**	**15**	**5**	**3**	**1**	**Proteasome subunit beta 9**	endopeptidase	
**Psme1**	**38**	**11**	**4**	**3**	**proteasome activator subunit 1**	endopeptidase	multiple signailng pathways
**Ptbp3**	**19**	**8**	**9**	**3**	**Polypyrimidine tract-binding protein 3**	NA & RNA binding	regulator of differentiation
**S100a13**	**5**	**3**			**S100 calcium binding protein A13**	calmodulin	binding & signaling
**Sept7**	**15**	**3**			**Septin-7**	cytoskeleton	binding
**Sept9**	**9**	**4**			**Septin-9**	cytoskeleton	binding
**Set**	**16**	**4**			**SET nuclear proto-oncogene**	chaperon, phosphatase inhibitor	cell cycle
**Sf3a1**	**6**	**4**	**1**	**1**	**Splicing factor 3A subunit 1**	RNA & protein binding	cell cycle
**Sf3b3**	**32**	**9**			**Splicing factor 3B subunit 3**	RNA & protein binding	splicing factor
**Siglec1**	**6**	**5**	**0**	**0**	**Sialoadhesin**	Ig superfam. adhesion, signaling	protein & carbohydrate binding
**Skap2**	**5**	**3**			**Src kinase-assoc. phosphoprotein 2**	protein binding	signal regulator
**Smarca5**	**12**	**5**			**regulator of chromatin, subfamily a5**	DNA helicase	biological processes
**Snd1**	**51**	**21**	**17**	**5**	**Staphyloc. nuclease & tudor dom.1**	transcription cofactor	protein, NA, RNA binding
**Snrpa**	**8**	**3**			**small nuclear ribonucleoprotein A**	RNA binding	splicing factor
**Snrpa1**	**10**	**5**			**small nuclear ribonucleoprotein A'**	RNA bindingr	splicing factor
**Snx5**	**13**	**4**	**1**	**1**	**Sorting nexin-5**	trafficking regulator	vesicle biogenesis, -mediated transport
**Sptan1**	**7**	**5**			**Spectrin alpha, non-erythrocytic 1**	non-motor actin binding	multiple signaling
**Srsf10**	**4**	**3**			**Serine/arginine-rich splicing factor 10**	RNA & nucleic acid binding	splicing factor
**Ssb**	**14**	**7**	**2**	**2**	**Sjogren syndrome antigen B**	tRNA / RNA binding	splicing factor
**Ssr4**	**29**	**5**			**Stomatostatin receptor type 4**	GPCR	signal transduction
**Stip1**	**13**	**6**			**Stress-induced-phosphoprotein 1**	chaperone	axon guidance
**Tfrc**	**30**	**9**			**Transferrin receptor**	receptor	clathrin-mediated endocytosis
**Tgm2**	**21**	**6**			**transglutaminase 2**	acyltransferase	apoptosis
**Thbs1**	**64**	**16**	**10**	**3**	**Thrombospondin-1**	integrin-, fibrinogen-, collagen V-bind.	platelet aggreg., aniogenesis, inflamm., matrix adhesion
**Tln2**	**26**	**6**			**Talin-2**	actin family (cytoskeleton)	actin filament assembly
**Tmed4**	**12**	**5**	**2**	**1**	**transmembrane p24 trafficking prot. 4**	transfer protein, vesicle coat	signal transduction
**Tpr**	**14**	**9**			**translocated promoter**	RNA-, protein-binding	SLC-mediated transmembrane transport
**Tpt1**	**30**	**4**			**tumor protein, translation.-control. 1**	microtubule binding	regulator of growth and differentiation
**Trex1**	**14**	**5**			**Three-prime repair exonuclease 1**	nuclease, hydrolase, protein binding	mismatch repair
**Tubb2a**	**166**	**15**			**Tubulin beta-2A**	GTP binding	membrane trafficking, vesicle-mediated transport
**U2af2**	**9**	**5**			**U2 snRnP auxiliary factor 2**	RNA & protein binding	splicing factor

Table S3B cont.

	MDSC *in vitro*		MDSC *ex vivo*				
**Synonym**	**Prot.M.**	**Pept.M.**	**Prot.M.**	**Pept.M.**	**Protein Description**	**Protein Family**	**Reactome pathway**
**Trex1**	**14**	**5**			**Three-prime repair exonuclease 1**		nuclease, hydrolase, protein binding
**Tubb2a**	**166**	**15**			**Tubulin beta-2A**	GTP binding	membrane trafficking, vesicle-mediated transport
**U2af2**	**9**	**5**			**U2 snRnP auxiliary factor 2**	splicing factor	protein-, RNA-binding
**U2surp**	**4**	**3**			**U2 snRNP-associated SURP motif**	mRNA processing	splicing
**Vps29**	**4**	**3**			**vacuolar protein sorting 29 homolog**	vesicle coat	protein & ion binding
**Xpo2**	**9**	**4**			**Exportin-2**	GTPase, transporter	protein binding, Ran GTPase binding

Table S3C

Proteins recovered at a significantly higher level in *ex vivo* than *in vitro* MDSC

	MDSC *in vitro*		MDSC *ex vivo*				
**Synonym**	**Prot.M.**	**Pept.M.**	**Prot.M.**	**Pept.M.**	**Protein Description**	**Protein Family**	**Reactome pathway**
**Atp6ap2**			**9**	**5**	**ATPase H+ transporting 2, Renin receptor**		angiotensin metabolism
**Atp6v0a1**			**23**	**8**	**ATPase, H+ transporting, V0 subunit A1**	ATP synthase, hydrolase	InsR signal., ROS, transmembr. & ion transp.
**Capzb**			**17**	**4**	**capping protein muscle Z-line, beta**	non-motor actin binding	b-tubulin-, actin-, protein-binding
**Casp6**			**6**	**4**	**Caspase-6**	protease, protease inhibitor	apoptotic execution phase
**Chmp2a**			**5**	**3**	**Charged multivesicular body protein 2a**	.	vesicle biogenesis, ECSRT, traffic, budding
**Cpt2**	**1**	**1**	**10**	**7**	**Carnitine O-palmitoyltransferase 2**	transferase	lipid / lipidprotein metabolism
**D1Pas1**			**8**	**4**	**DNA segment, Chr 1, Pasteur Institute 1**	RNA helicase	ATP-, RNA-, DNA-binding
**Dnajc10**	**2**	**1**	**13**	**5**	**DnaJ Hsp family (Hsp40) member C10**	.	chaperone binding, Hsp70 binding
**Emc1**			**16**	**8**	**ER membrane protein complex subunit 1**	.	molecular function
**Erlin2**			**15**	**6**	**ER lipid raft associated 2**	.	transmembrane transport
**Fundc2**			**7**	**4**	**FUN14 domain-containing 2**	.	molecular function
**Galnt7**	**1**	**1**	**7**	**6**	**N-acetylgalactosaminyltransferase 7**	transferase	glycosylation
**Gas7**			**6**	**5**	**Growth arrest-specific 7**	actin family	membrane trafficking regulator
**Glrx**			**8**	**4**	**Glutaredoxin**	oxidoreductase	electron carrier, nucleotide metabolism
**Igf2r**			**5**	**3**	**insulin-like growth factor 2 receptor**	receptor	clathrin endocytos., memb. traffic, vesicle biogen.
**Lmna**			**102**	**25**	**Lamin-A/C Lmna**	intermediate filament	apoptotic execution, cell cycle
**Lmnb2**			**17**	**3**	**Lamin-B2**	intermediate filament	structural molecule
**Mmp12**	**1**	**1**	**18**	**6**	**matrix metallopeptidase 12**	.	ECM organization, ECM degradation
**Mrpl41**			**7**	**4**	**mitochondrial ribosomal protein L41**	ribosomal protein	mitochondrial biogenesis and maintenance
**Mrpl49**			**3**	**3**	**mitochondrial ribosomal protein L49**	ribosomal protein	mitochondrial biogenesis and maintenance
**Mtch2**			**16**	**5**	**Mitochondrial carrier 2**	transfer / carrier protein	molecular function
**Myof**	**4**	**3**	**186**	**52**	**Myoferlin**	membrane traffic	phospholipid binding
**Nono**			**6**	**3**	**Non-POU domain-con. octamer-binding**	mRNA splicing	protein-, RNA-, DNA-binding
**Nup155**			**8**	**5**	**nucleoporin 155**	.	heat stress regulation, SLC-mediated transport
**Nup210**	**3**	**1**	**45**	**12**	**nucleoporin 210**	membrane traffic protein	heat stress response, transmembrane transport
**Olfm4**			**8**	**5**	**Olfactomedin-4**	receptor, structural protein	protein binding

Table S3C cont.

	MDSC *in vitro*		MDSC *ex vivo*				
**Synonym**	**Prot.M.**	**Pept.M.**	**Prot.M.**	**Pept.M.**	**Protein Description**	**Protein Family**	**Reactome pathway**
**Parp1**			**4**	**3**	**poly (ADP-ribose) polymerase family 1**	DNA ligase	TGFbR complex signaling
**Pdha1**			**13**	**6**	**pyruvate dehydrogenase alpha 1**	dehydrogenase	RA signaling, electron transport, sign. transduct.
**Pfkp**	**2**	**1**	**13**	**6**	**phosphofructokinase, platelet**	carbohydrate kinase	glucose metabolism
**Ptbp1**			**18**	**5**	**Polypyrimidine tract-binding protein 1**	.	mRNA splicing, FGFR signaling
**Samm50**			**6**	**3**	**SAMM50 sorting and assembly machinery**	protein binding and sorting	protein assembly
**Sel1l**	**7**	**4**	**23**	**13**	**SEL1L ERAD E3 ligase adaptor subunit**	enzyme modulator	transmembrane transport, NOTCH signaling
**Slc25a13**			**47**	**8**	**solute carrier family 25 member 13**	AA transporter, protein transfer	glucose metabolism
**Snap29**			**5**	**3**	**Synaptosomal-associated protein 29**	SNARE protein	membrane traffic, vesicle-mediated transport
**Snx12**			**4**	**3**	**Sorting nexin-12**	phosphatidyl & lipid bindingr	membrane traffick regulato
**Ssr2**	**0**	**0**	**46**	**4**	**signal sequence receptor subunit 2**	G-protein-coupled receptor	GPCR signaling
**Timm50**			**6**	**4**	**translocase of inner mitoch. membrane 50**	phosphatase	IL2R binding
**Tomm40**			**14**	**5**	**translocase of outer mitoch. membrane 40**	membrane trafficking	protein channel, transmembrane transport
**Trpv2**	**2**	**1**	**18**	**5**	**Transient receptor cation channel V 2**	ion channel	transmembrane transport
**Uqcrc1**	**7**	**2**	**46**	**7**	**ubiquinol-cytochrome c reductase I**	metalloprotease	electron transport

Table S4

Protein profile of MDSC-Exosomes from in vitro / ex vivo MDSC

Table S4A

Top score peptides of MDSC-Exosomes from *in vitro* / *ex vivo* MDSC[a]

		MDSC-Exo *in vitro*		MDSC-Exo *ex vivo*	
**Synonym**	**Protein Description**	**Prot.M.**	**Pept.M.**	**Prot.M.**	**Pept.M.**
Actb	Actin, cytoplasmic 1	477	42	589	38
Actr3	Actin-related protein 3	34	12	47	13
Ahcy	Adenosylhomocysteinase	21	7	53	10
Anxa1	Annexin A1	88	18	23	11
Anxa11	Annexin A11	53	10	29	6
Anxa2	Annexin A2	108	18	34	15
Anxa4	Annexin A4	56	14	23	10
Anxa5	Annexin A5	43	17	34	13
Anxa6	Annexin A6	64	19	10	3
Ap2m1	AP-2 complex subunit mu	21	10	2	1
Arpc2	Actin-related protein 2/3 complex subunit 2	.		24	12
C3	Complement C3	119	16	152	15
Cap1	Adenylyl cyclase-associated protein 1	19	11	38	10
Ccdc47	Coiled-coil domain-containing 47	33	14		
Cct2	chaperonin containing TCP1 subunit 2	39	12	18	9
Cct3	chaperonin containing TCP1 subunit 3	32	10	4	4
Cct4	chaperonin containing TCP1 subunit 4	32	10	7	4
CD11b	Integrin alpha-M	83	21	39	16
CD13	Alanyl aminopeptidase, membrane	36	16		
CD18	Integrin beta-2	78	27	32	17
Clic1	Chloride intracellular channel 1	26	10	26	7
Cltc	Clathrin heavy chain 1	208	50	14	9
Coro1a	Coronin-1A	41	10	24	8
Ctsb	Cathepsin B	9	5	47	10
Ctsd	Cathepsin D	25	6	52	10
Dera	Deoxyribose-phosphate aldolase	11	6	20	12
Eea1	Early endosome antigen 1	113	13	91	12
Eif3a	Eukaryotic translation initiation factor 3 A	127	23		
Eif3b	Eukaryotic translation initiation factor 3 B	42	17		
Eif3c	Eukaryotic translation initiation factor 3 C	128	19		
Eif3e	Eukaryotic translation initiation factor 3 E	49	18	13	8
Eif3l	Eukaryotic translation initiation factor 3 L	32	10		
Eif4a1	Eukaryotic initiation factor 4A1	46	15	26	12
Emilin2	elastin microfibril interfacer 2	20	10		
Eno1	enolase 1	194	20	152	23
Ezr	Ezrin	33	12		
Fasn	Fatty acid synthase	27	16	2	1
Fermt3	Fermitin family member 3	33	14	65	18
Flna	Filamin-A	27	16	23	10
Fn1	Fibronectin 1	580	71	71	25
Fth1	Ferritin heavy chain	171	15	132	12
Ftl1	Ferritin light chain 1	226	14	271	13
Gapdh	Glyceraldehyde-3-phosphate dehydrogenase	210	13	215	13
Gdi2	GDP dissociation inhibitor 2	21	12	13	8
Gsn	Gelsolin	86	11		
H2-Ab1	histocompatibility 2, class II antigen A, beta 1	53	10	37	9
Hbb-b1	hemoglobin, beta adult major chain	205	10	507	13
Hbb-b2	hemoglobin, beta adult minor chain	122	10	306	13
Hmgb1	High mobility group box 1	95	11	16	4
Hmgb2	High mobility group box 2	52	10	26	7
Hsp90ab1	Heat shock protein HSP 90-beta	133	17	102	18

Table S4A cont.

Synonym	Protein Description	MDSC-Exo *in vitro* Prot.M.	MDSC-Exo *in vitro* Pept.M.	MDSC-Exo *ex vivo* Prot.M.	MDSC-Exo *ex vivo* Pept.M.
**Hspa5**	**heat shock protein 5**	**43**	**11**	**22**	**4**
**Hspa8**	**Heat shock cognate 71 kDa protein**	**217**	**25**	**208**	**25**
**Ighm**	**Ig mu chain C region**	**9**	**5**	**86**	**14**
**Iqgap1**	**IQ motif containing GTPase activating protein 1**	**46**	**15**	**2**	**1**
**Klhdc4**	**Kelch domain-containing 4**	**27**	**11**		
**Krt73**	**Keratin 73**	**31**	**4**	**67**	**13**
**LDHA**	**L-lactate dehydrogenase A chain**	**49**	**9**	**38**	**10**
**Lgals3bp**	**Galectin-3-binding protein Lgals3bp**	**149**	**21**	**116**	**19**
**Lpl**	**Lipoprotein lipase**	**174**	**12**	**59**	**9**
**Ltf**	**Lactotransferrin**	**98**	**29**	**89**	**26**
**Mfge8**	**Lactadherin**	**120**	**18**	**50**	**14**
**Mmp12**	**matrix metallopeptidase 12**	**324**	**22**	**229**	**20**
**Mmp19**	**matrix metallopeptidase 19**	**56**	**14**	**39**	**10**
**Mpo**	**Myeloperoxidase**	**15**	**9**	**56**	**16**
**Msn**	**Moesin**	**52**	**19**	**22**	**8**
**Mvp**	**Major vault protein**	**26**	**14**		
**Myh1**	**myosin heavy chain 1**	**.**		**54**	**13**
**Myh4**	**myosin heavy chain 4**	**.**		**63**	**15**
**Myh9**	**myosin heavy chain 9**	**235**	**60**	**69**	**20**
**Ncl**	**Nucleolin**	**302**	**30**	**13**	**7**
**Pdcd6ip**	**Programmed cell death 6-interacting protein**	**118**	**27**	**12**	**6**
**Pgam1**	**Phosphoglycerate mutase 1**	**19**	**9**	**19**	**10**
**Pkm**	**Pyruvate kinase PKM**	**.**		**186**	**24**
**Ppib**	**Peptidyl-prolylisomerase B**	**39**	**12**	**26**	**9**
**Prdx1**	**Peroxiredoxin-1**	**79**	**10**	**16**	**6**
**Prkcd**	**protein kinase C delta**	**222**	**25**		
**Psma1**	**Proteasome subunit alpha 1**	**34**	**9**	**38**	**11**
**Psma5**	**Proteasome subunit alpha 5**	**29**	**8**	**33**	**10**
**Psma6**	**Proteasome subunit alpha 6**	**34**	**10**	**39**	**10**
**Psma7**	**Proteasome subunit alpha 7**	**30**	**11**	**43**	**10**
**Psmb8**	**Proteasome subunit beta 8**	**34**	**8**	**49**	**11**
**Psmd11**	**proteasome 26S subunit, non-ATPase 11**	**37**	**13**	**7**	**4**
**Psmd2**	**proteasome 26S subunit, non-ATPase 2**	**32**	**19**	**3**	**3**
**Psmd3**	**proteasome 26S subunit, non-ATPase 3**	**24**	**13**	**2**	**1**
**Psmd6**	**proteasome 26S subunit, non-ATPase 6**	**35**	**16**	**19**	**10**
**Rab11b**	**RAB11B, member RAS oncogene family**	**35**	**11**	**30**	**9**
**Rack1**	**Receptor of activated protein C kinase 1**	**33**	**12**	**50**	**14**
**Rplp0**	**ribosomal protein P0**	**36**	**7**	**38**	**10**
**Ruvbl2**	**RuvB like AAA ATPase 2**	**20**	**10**	**4**	**3**
**Ssrp1**	**structure specific recognition protein 1**	**48**	**10**		
**Tcp1**	**T-complex 1**	**42**	**14**	**2**	**1**
**Thbs1**	**Thrombospondin-1**	**60**	**17**	**104**	**23**
**Tln1**	**Talin-1**	**24**	**14**		
**Tuba1b**	**Tubulin alpha-1B**	**127**	**18**	**210**	**36**
**Tuba1c**	**Tubulin alpha-1C**	**115**	**17**	**145**	**17**
**Tuba4a**	**Tubulin alpha-4A**	**91**	**16**	**144**	**18**
**Tubb4b**	**Tubulin beta-4B**	**107**	**19**	**120**	**19**
**Tubb5**	**Tubulin beta-5**	**115**	**20**	**124**	**19**
**Uba1**	**Ubiquitin-like modifier-activating enzyme 1**	**33**	**10**	**12**	**5**
**Vcp**	**valosin containing protein**	**226**	**41**	**37**	**14**

[a] Only proteins are shown with ≥10 significant peptides by at least one MDSC-Exo population

Table S4B

Higher recovery in Exosomes from *in vitro* MDSC

	Exo - *in vitro*		Exo - *ex vivo*				
**Synonym**	**Prot.M.**	**Pept.M.**	**Prot.M.**	**Pept.M.**	**Protein Description**	**Molecular function**	**Reactome pathway**
**Actn1**	8	4			**actinin alpha 1**	integrin, actin, vinculin binding	cell junction organization, cell-cell communication
**Adam10**	9	5			**Disintegrin and metalloproteinase 10**	metallopeptidase	NOTCH, Ephrin, EGFR signaling
**Anp32b**	5	3			**Acidic nuclear phosphoprotein 32B**	RNA & histone & protein binding	apoptosis, differentiation
**Anxa6**	64	19	10	3	**Annexin A6**	protein complex & cholesterol binding	contraction
**Ap2a2**	10	4			**AP-2 complex subunit alpha-2**	lipid & protein binding	clathrin endocytosis, lipid transport, vesicle transport
**Ap2b1**	28	6			**AP-2 complex subunit beta**	transporter	lipid & vesicle transport, clathrin endocytosis, signaling
**Ap2m1**	21	10	2	1	**AP-2 complex subunit mu**	binding, signaling	lipid transport, Gap junction traffic, clathrin endoc.
**Arrb2**	13	6			**arrestin beta 2**	enzyme modulator	clathrin endocyt., vesic.-med.transport, GPCR signaling
**Ass1**	16	8			**Argininosuccinate synthase 1**	binding, ligase	AA & polyamine metabolism
**Bin2**	17	4			**Bridging integrator 2**	phospholipid binding,kinase	brassinosteroid signaling pathway
**Brox**	4	3			**BRO1 domain and CAAX motif**	protein binding, molecular function	
**Capg**	7	4			**capping actin protein, gelsolin like**	actin binding, protein complex binding	actin filament capping
**Capns1**	9	3			**Calpain small subunit 1**	endopeptidase	
**Cbx3**	11	5			**Chromobox 3**	transcription factor	epigenetic regul. of gene expression
**Ccdc47**	33	14			**Coiled-coil domain-containing 47**	ion & RNA binding	ER organization, osteopblast differention
**Ccl9**	13	3			**C-C motif chemokine 9**	cytokine & chemokine activity	GPCR signaling
**Cct6a**	20	7	7	2	**chaperonin TCP1 subunit 6A**	chaperonin	protein folding
**Cct7**	5	3			**chaperonin containing TCP1 subunit 7**	ATP & protein binding	chaperon- & protein folding
**Cct8**	13	7	2	1	**chaperonin TCP1 subunit 8**	chaperonin	protein folding
**CD13**	36	16			**Alanyl aminopeptidase 13**	metalloprotease	angiogenesis, signaling, proteolysis
**CD316**	24	8	3	2	**Immunoglobulin superfamily 8**	protein binding	CD81 partner, cell component movement
**CD36**	14	7	1	1	**CD36, platelet glycoprotein 4**	lipoprotein binding	TLR cascade, vesicle-mediated transport
**Cltc**	208	50	14	9	**Clathrin heavy chain 1**	vesicle coat	lipid transport, vesicle transport & biog., clathrin endoc.
**Copb2**	10	5			**coatomer complex beta 2**	vesicle coat, structural molecule	transport, vesicle-med.trasport,, membrane traffic.
**Csnk2a1**	6	4	2	1	**Casein kinase II alpha 1**	PTK, phosphatase regulator	signaling, cell cycle
**Cyp4f3**	17	6	3	1	**cytochrome P450 family 4F3**	oxygenase	Lipid metabolism
**Ddost**	6	3			**DDoligosaccharide-glycosyltransferase**		T cell activ., neutrophil degranulation, cytokine response
**Dek**	38	9			**DEK proto-oncogene**	DNA & histone binding	regulation of gene expression & AP-2 transcription
**Dhx9**	5	4			**DEAH-box helicase 9**	hydrolase, protein binding	MyD88, TLR, type I IFN signaling cascade
**Dip2b**	4	3			**Disco-interacting protein 2 homolog B**	molecular function, catalytic activity	
**Dnaja2**	6	3			**DnaJ heat shock protein member A2**	glycoprotein & HSP binding	cochaperone activity, protein refolding
**Dync1h1**	32	21	3	2	**dynein cytoplasmic 1 heavy chain 1**	microtubule activity	Vesicle & vesicle-med. transport, organelle biogenesis
**Eftud2**	14	8			**elongation factor Tu GTP binding 2**	hydrolase, translation initiat & elong.	splicing
**Eef1d**	3	3			**Elongation factor 1-delta**	translation elongation	translation, protein metabolism
**Eif3a**	127	23			**Eukaryotic translation initiation 3 A**	translation initiation	
**Eif3b**	42	17			**Eukaryotic translation initiation 3 B**	translation initiation	

Table S4B cont.

	Exo - *in vitro*		Exo - *ex vivo*				
**Synonym**	**Prot.M.**	**Pept.M.**	**Prot.M.**	**Pept.M.**	**Protein Description**	**Molecular function**	**Reactome pathway**
**Eif3c**	**128**	**19**			**Eukaryotic translation initiation 3 C**	translation initiation	
**Eif3i**	**11**	**6**	**1**	**1**	**Eukaryotic translation initiation 3 I**	translation initiation, protein binding	
**Eif3l**	**32**	**10**			**Eukaryotic translation initiation 3 L**	translation initiation	
**Emilin2**	**20**	**10**			**elastin microfibril interfacer 2**	ECM protein	cell adhesion
**Esyt1**	**22**	**9**	**1**	**1**	**Extended synaptotagmin-1**	ion & lipid binding	lipid metabolism
**Ezr**	**33**	**12**			**Ezrin**	cytoskeletal protein binding	Netrin1 signaling
**Fasn**	**27**	**16**	**2**	**1**	**Fatty acid synthase**	hyrolysae, transferase, ligase	lipid metabolism
**Fgl2**	**11**	**5**			**Fibroleukin**	peptidase, signaling	signaling
**Gca**	**4**	**3**			**Grancalcin**	dimerization, metal ion binding	membrane fusion, neutrophil degranulation
**Glg1**	**5**	**3**			**Golgi apparatus protein 1**	fibroblast growth factor binding	hemostasis
**Gm2a**	**6**	**3**			**GM2 ganglioside activator**	enzyme activator, hydrolase	lipid metabolism
**Gpi**	**5**	**3**			**Glucose-6-phosphate isomerase**	growth factor activity	carbohydrate metabolism
**Gsn**	**86**	**11**			**Gelsolin**	actin & myosin binding	apoptosis, programmed cell death
**Hnrnpk**	**18**	**5**	**4**	**1**	**Heterogeneous ribonucleoprotein K**	enzyme modulator	splicing
**Iqgap1**	**46**	**15**	**2**	**1**	**IQ motif GTPase activating protein 1**	G-protein modulator	Rho GTPase effector signaling
**Klhdc4**	**27**	**11**			**Kelch domain-containing 4**	molecular function	
**Lamp2**	**5**	**3**			**Lysosome-associated glycoprotein 2**	protein domain specific binding	platelet activation & signaling
**Lcp1**	**20**	**9**	**3**	**2**	**lymphocyte cytosolic protein 1**	actin & protein binding	T cell activation, IL12 & PKA signaling
**M6pr**	**11**	**4**	**1**	**1**	**mannose-6-phosphate receptor**	GPCR activity	clathrin endocytosis, vesicle transport
**Man2a1**	**12**	**8**			**mannosidase 2, alpha 1**	hydrolase, binding	protein transport & modification
**Mvp**	**26**	**14**			**Major vault protein**	phosphatase & kinase binding	InsR signaling, transport, endocytosis
**Myadm**	**10**	**3**			**Myeloid-assoc. differentiation marker**		raft organization, migration
**Mybbp1a**	**10**	**3**			**Myb-binding protein 1A**	transcription corepressor, prot.binding	epigenetic regulation
**Myh9**	**235**	**60**	**69**	**20**	**myosin heavy chain 9**	actin & protein & RNA binding	membrane traffic, Rho GTPase signaling
**Ncl**	**302**	**30**	**13**	**7**	**Nucleolin**	RNA & protein binding	LIF response, angiogenesis
**Nrdc**	**10**	**7**			**nardilysin convertase**	metalloprotease	migration, proliferation
**Opn**	**20**	**3**			**secreted phosphoprotein 1,Osteopontin**	ECM binding, cytokine activity	ECM organization, signal transduction
**Pabpc1**	**15**	**8**			**poly(A) binding protein 1**	RNA & protein binding	splicing, RNA silencing, translation
**Pcbp1**	**5**	**3**			**Poly(rC)-binding protein 1**	DNA & protein binding	splicing
**Pdcd6ip**	**118**	**27**	**12**	**6**	**Programmed cell death 6-interact**	protein binding	protein binidng
**Pfkp**	**4**	**3**			**phosphofructokinase, platelet**	transferase and kinase activity	Glucose metabolism
**Pfn1**	**82**	**7**			**Profilin-1**	binding	Rho GTPase signaling
**Prkcd**	**222**	**25**			**protein kinase C delta**	non-receptor kinase	IFN & cytokine & multiple signaling pathways
**Psma2**	**21**	**8**			**Proteasome subunit alpha 2**	endopeptidase	ABC transporter, multiple signaling pathways
**Psmb5**	**7**	**3**			**Proteasome subunit beta 5**	endopeptidase	multiple signaling pathways
**Psmc3**	**7**	**3**			**proteasome 26S subunit, ATPase 3**	hydrolase	multiple signaling pathways
**Psmd1**	**12**	**7**	**2**	**1**	**proteasome 26S subunit 1**	enzyme regulator	multiple signaling cascades

Table S4B cont.

	Exo - *in vitro*		Exo - *ex vivo*				
**Synonym**	**Prot.M.**	**Pept.M.**	**Prot.M.**	**Pept.M.**	**Protein Description**	**Molecular function**	**Reactome pathway**
**Psmd11**	**37**	**13**	**7**	**4**	**proteasome 26S subunit 11**	protein binding, structural molecule	multiple signaling cascades
**Psmd13**	**19**	**7**	**1**	**1**	**proteasome 26S subunit 13**	endopeptidase. structural molecule	APC transport, multiple signaling pathways
**Psmd2**	**32**	**19**	**3**	**3**	**proteasome 26S subunit 2**	enzyme regulator	multiple signaling pathways
**Psmd3**	**24**	**13**	**2**	**1**	**proteasome 26S subunit 3**	enzyme regulator	multiple signaling pathways
**Pygl**	**14**	**7**			**Glycogen phosphorylase L**	phosphorylase	carbohydrate metabolism
**Rab10**	**26**	**3**			**RAB10, member RAS oncogene family**	GTPase, myosinV binding	membrane traffic, vesicle transport
**Rap2b**	**4**	**3**			**RAP2B, member of RAS oncogene fam.**	GTPase	signal transduction, platelet activation
**Rdx**	**29**	**10**			**Radixin**	actin binding	RAP & PKA signal., junction organiz., endosome traffic.
**Rnh1**	**6**	**4**			**ribonuclease/angiogenin inhibitor 1**	nuclease, hydrolase	
**Rpl3**	**9**	**4**			**ribosomal protein L3**	RNA binding	RNA processing
**Rtn4**	**28**	**8**	**6**	**2**	**Reticulon-4**	protein binding	signal transduction
**Ruvbl1**	**17**	**8**	**4**	**2**	**RuvB like AAA ATPase 1**	protein binding	protein metabolism, signal transduction
**Ruvbl2**	**20**	**10**	**4**	**3**	**RuvB like AAA ATPase 2**	protein binding	DNA repair, transcription
**Scamp3**	**8**	**4**			**secretory carrier membrane protein 3**	ubiquitin ligase binding	vesicle-mediated transport
**Sf3b3**	**12**	**6**			**Splicing factor 3B subunit 3**	RNA & protein binding	splicing
**Slc25a5**	**9**	**4**	**1**	**1**	**solute carrier family 25 member 5**	transporter	energy metabolism
**Smarca5**	**15**	**5**			**Regulator of chromatin a5**	hydrolase, protein binding	epigenetic gene regulation
**Snrpb**	**10**	**3**			**Small nuclear ribonucleoprotein B & B1**	protein & RNA binding	splicing
**Ssrp1**	**48**	**10**			**structure specific recognition protein 1**	transcription factor, signaling	transcriptional regulation
**Stk24**	**17**	**6**			**Serine/threonine-protein kinase 24**	protein kinase	Apoptosis, programmed cell death
**Tcp1**	**42**	**14**	**2**	**1**	**T-complex 1**	peptidase, chaperonin	chaperonin & protein folding, protein metabolism
**Tln1**	**24**	**14**			**Talin-1**	cytoskeleton organization, binding	integrin signaling
**Tm9sf2**	**15**	**9**			**Transmembrane 9 member 2**	transporter, molecular function	
**Tm9sf3**	**8**	**3**			**Transmembrane 9 superfamily 3**	transporter, molecular function	
**Tmed4**	**7**	**4**			**transmembrane p24 trafficking protein 4**	signal transducer	transmembrane transport
**Tmed9**	**11**	**5**	**2**	**1**	**transmembrane p24 protein 9**	transfer / carrier, vesicle coat	transport & vesicle-med. transport
**Tra2b**	**36**	**4**	**1**	**1**	**Transformer-2 beta homolog**	nucleic acid & RNA & protein binding	splicing
**U2surp**	**16**	**6**			**U2 snRNP-associated SURP**	mRNA processing	splicing
**Vat1**	**10**	**5**			**vesicle amine transport 1**	reductase, AA transporter	transmembrane transporter
**Vcp**	**226**	**41**	**37**	**14**	**valosin containing protein**	hydrolase, protein binding	APC & small molecule transporter, stress response
**Vim**	**20**	**7**			**Vimentin**	structural protein	apoptotic cleavage, programmed cell death
**Vrk1**	**7**	**4**	**2**	**1**	**vaccinia related kinase 1**	protein kinase, ATP binding	cell cycle
**Wdr5**	**10**	**5**	**1**	**1**	**WD repeat domain 5**	esterase, kinase inhibitor, splicing	HOX gene activation
**Ybx1**	**16**	**5**	**1**	**1**	**Y-box binding protein 1**	transcription activator	transcription, splicing
**Ypel5**	**7**	**4**			**yippee-like 5**	metal ion binding	neutrophil degranulation

Table S4C

Higher recovery in Exosomes from *ex vivo* MDSC

	Exo - *in vitro*		Exo - *ex vivo*				
**Synonym**	**Prot.M.**	**Pept.M.**	**Prot.M.**	**Pept.M.**	**Protein Description**	**Molecular function**	**Reactome pathway**
**Adh5**	.		5	3	**Alcohol dehydrogenase class-3**	oxidoreductase, ion & fatty acid binding	metabolism, oxidation
**Alad**	.		13	7	**aminolevulinate dehydratase**	lyase, ion & protein binding	Porphyrin & heme metabolsim
**Apoe**	10	2	22	8	**Apolipoprotein E**	protein & lipid binding	vesicle & lipid transport, signaling
**Aprt**	.		8	3	**Adenine phosphoribosyltransferase**	transferase, AMP binding	purine metabolism
**Arf4**	.		22	4	**ADP-ribosylation factor 4**	GTP & protein binding	organelle biogenesis, trafficking & transport
**Arpc2**	.		24	12	**Actin-related protein 2/3 subunit 2**	cytoskeleton, protein complex binding	Rho GTPase & Ephrin signal., endocyt., ves.transport
**C1qc**	3	1	5	4	**complement C1q C chain**	endopeptidase	complement cascade, granuloc. & Mϕ differentiation
**Ca2**	2	1	8	4	**Carbonic anhydrase 2**	dehydrase, lyase, metal ion binding	O2/CO2 metabolism
**CD147**	.		4	3	**Basigin**	carbohydrate binding	ECM organization, membrane transport
**CD177**	3	2	14	9	**CD177 antigen Cd177**	molecular function	fibrin clot formation
**CD41**	2	1	6	5	**Integrin alpha-IIb**	protein binding	integrin signaling, ECM organization
**Cfp**	.		143	7	**complement factor properdin**	ECM component	complement activation
**Ctsg**	1	1	13	4	**Cathepsin G**	serine protease	ECM organization, MMP activation, IGF transport
**Hgd**	.		3	3	**Homogentisate 1,2-dioxygenase**	oxidoreductase	AA metabolism
**Hspa1a**	.		38	6	**Heat shock 70 kDa protein 1A**	protein binding	stress response
**Hspa1l**	.		41	5	**Heat shock 70 kDa protein 1-like**	protein folding, GPCR binding	
**Ilk**	.		7	6	**Integrin-linked kinase**	non-receptor tyrosine kinase	ECM & cell junction organization
**Itih2**	.		100	7	**Inter-alpha-trypsin inhibitor H2**	protease inhibitor	hyaluronan & protein metabolism
**Itm2b**	.		5	4	**Integral membrane protein 2B**	protein binding	protein metabolism, amyloid precursor regulation
**Jup**	.		12	7	**Junction plakoglobin**	protein binding	junction organization, VEGF signaling
**Myh1**	.		54	13	**myosin heavy chain 1**	G-protein modulator	
**Myh4**	.		63	15	**myosin heavy chain 4**	G protein modulator	membrane & vesicle med. trafficking
**Ngp**	1	1	19	4	**Neutrophilic granule protein**	peptidase inhibitor	defense, angigoenesis regulation
**Pkm**	.		186	24	**Pyruvate kinase PKM**	catalytic activity, protein binding	carbohydrate metabolism
**Prdx2**	1	1	9	4	**Peroxiredoxin-2**	protein binding, oxidase	stress response
**Prdx6**	.		10	6	**Peroxiredoxin-6**	binding, catalytic & hydrolase activity	stress response
**Psap**	1	1	8	6	**Prosaposin**	immune defense, surfactant	TLR signaling
**Rab5a**	.		17	5	**RAB5A, RAS oncogene family**	GTPase	clathrin endocyt., membr. & ves. transport, ves. biogen
**Rpl7**	7	1	18	5	**ribosomal protein L7**	DNA & RNA binding	translation initiation
**Rps17**	.		8	4	**ribosomal protein S17**	structural ribosome constituent	translation, mRNA catabolism
**Tfrc**	.		20	5	**Transferrin receptor**	receptor activity, protein binding	clathrin endocyt., membr. & ves. transport, ves. biogen.
**Ube2n**	.		4	3	**Ubiquitin-conjugating enzyme E2 N**	transferase, protein binding	IFN-, cytokine-,TLR signaling
**Vcl**	.		5	3	**Vinculin**	Rho GTPase, protein & actin binding	platelet activation & signaling
**Ywhaq**	.		18	3	**14-3-3 protein theta**	chaperone, protein binding	Rho GTPase sign., BAD activ., vesicle transport

Table S5

Differences in MDSC versus MDSC-Exo protein profile

Table S5A

Preferential peptide recovery in MDSC-Exo

	MDSC		MDSC-Exo				
**Synonym.**	**Prot.M.**	**Pept.M.**	**Prot.M.**	**Pept.M.**	**Protein Description**	**Function**	**Reactome pathway**
**Ap2m1**	**5**	**2**	**21**	**10**	**AP-2 complex mu**	protein binding, receptor	trafficking, transmission, endocytosis, signaling
**Ap2S1**	**.**		**11**	**5**	**AP-2 complex sigma**	vesicle coat protein	lipid & vesicel transport, endocytosis, cargo recognition, signaling
**Arrb2**	**2**	**1**	**13**	**6**	**arrestin beta 2**	enzyme modulator	multiple signaling pathways (HH, WNT5A, FZD4, GPCR),
**Atp6ap2**	**.**		**9**	**6**	**ATPase H+ transporting 2**	enzyme binding, receptor	metabolism (angiotensin)
**C1qtnf3**	**.**		**7**	**2**	**C1q and TNF related protein 3**	protein binding	complement cascade
**C3**	**19**	**5**	**119**	**16**	**Complement C3**	cytokine, protease inhibitor	GPCR signaling, complement activation
**Casp6**			**5**	**4**	**Caspase-6**	peptidase	apoptosis execution
**Ccdc47**	**3**	**2**	**33**	**14**	**Coiled-coil domain- 47**	calcium & RNA binding	Ca++ homeostasis, ER organization, osteoblast differentiation
**Ccl9**	**.**		**13**	**4**	**C-C chemokine 9**	chemokine	GPCR signaling
**Cct4**	**9**	**3**	**32**	**10**	**chaperonin TCP1 subunit 4**	chaperonin	protein folding & metabolism
**CD316**	**.**		**24**	**8**	**Immunoglobulin superfamily 8**	protein binding	proliferation, cell component movement
**CD81**	**.**		**14**	**5**	**CD81 antigen**	adhesion, signaling	immune regulation
**Col1a1**	**.**		**4**	**4**	**collagen type I alpha 1**	protein binding, ECM	ECM organization
**CSN3**	**.**		**3**	**2**	**COP9 signalosome subunit 3**	molecular function	endocytosis, cargo recogn., membrane traffic, vesicle transport
**CSN4**	**.**		**4**	**4**	**COP9 signalosome subunit 4**	protein binding, protease	excision repair, endocytosis, vesicle transport
**CSN6**	**.**		**4**	**4**	**COP9 signalosome subunit 6**	transcription, peptidase	transcription, endocytosis, membrane traffic, vesicle transport
**CSN8**	**.**		**3**	**2**	**COP9 signalosome subunit 8**	protein binding, signaling	endocytosis, trafficking, cargo recognition, vesicle transport
**Cyp4f3**	**3**	**2**	**17**	**6**	**cytochrome P450 family 4F3**	oxygenase	lipid metabolism
**Dek**	**4**	**2**	**38**	**9**	**DEK proto-oncogene**	DNA / RNA binding	regulation of gene expressio
**Dera**	**.**		**11**	**6**	**Deoxyribose-phosphate aldolase**	lyase	carbohydrate metabolism
**Eea1**	**5**	**4**	**113**	**13**	**Early endosome antigen 1**	ion/protein binding	TLR cascades
**Eftud2**	**.**		**14**	**8**	**elongation factor Tu 2**	G protein, hydrolase	splicing
**Eif3a**	**9**	**4**	**121**	**21**	**Eukaryotic initiation factor 3 A**	translation	translation initiation
**Eif3b**	**21**	**4**	**42**	**17**	**Eukaryotic initiation factor 3 B**	protein/RNA binding, transcript.	translation, metabolism
**Eif3c**	**3**	**2**	**128**	**19**	**Eukaryotic initiation factor 3 C**	translation	translation initiation
**Eif3e**	**2**	**2**	**49**	**18**	**Eukaryotic initiation factor 3 E**	binding, translation	translation initiation
**Eif3f**	**8**	**1**	**42**	**9**	**Eukaryotic initiation factor 3 F**	peptidase, translation	translation, protein metabolism
**Eif3g**	**1**	**1**	**20**	**5**	**Eukaryotic factor 3 G**	binding, translation	translation initiation
**Eif3l**	**1**	**1**	**32**	**10**	**Eukaryotic initiation factor 3 L**	translation	translation initiation
**Eif3m**	**2**	**1**	**27**	**7**	**Eukaryotic initiation factor 3 M**	translation	translation, protein metabolism
**Emilin2**	**.**		**20**	**10**	**elastin microfibril interfacer 2**	ECM protein	cell adhesion
**Fgl2**	**.**		**11**	**5**	**fibroleukin**	signaling, peptidase	neutrophil degranulation
**Fn1**	**.**		**580**	**71**	**Fibronectin 1**	binding, signaling	matrix formation, multiple sign.
**H2afy**	**.**		**82**	**14**	**Core histone H2A.1**	histone, binding	cell cycle, rRNA regulation

Table S5A cont.

	MDSC		MDSC-Exo				
**Synonym.**	**Prot.M.**	**Pept.M.**	**Prot.M.**	**Pept.M.**	**Protein Description**	**Function**	**Reactome pathway**
**Hist1h3a**	**.**		**64**	**5**	**Histone H3.1**	histone	multiple signaling pathways, stress response
**Hist1h3b**	**.**		**69**	**5**	**Histone H3.2**	histone, binding	cytokine/interleukin signaling, stress response
**Hist2h2aa1**	**.**		**107**	**7**	**Histone H2A type 2A**	histone	metalloprotease, cell cycle regulation
**Ighm**	**.**		**9**	**5**	**Ig mu chain C region**	binding	BCR signaling
**Klhdc4**	**.**		**27**	**11**	**Kelch domain-containing 4**	molecular function	
**Lpl**	**.**		**174**	**12**	**Lipoprotein lipase**	esterase, lipase	retinoid/lipid metabolsim/transport
**Mfge8**	**19**	**5**	**120**	**18**	**Lactadherin**	binding, protease, transporter	signaling, transporter
**Mmp12**	**1**	**1**	**324**	**22**	**matrix metallopeptidase 12**	peptidase	ECM organization/degradation
**Mmp19**	**.**		**56**	**14**	**matrix metallopeptidase 19**	metallopeptidase	ECM degradation
**Nrdc**	**.**		**10**	**7**	**nardilysin convertase**	metalloprotease	migration, proliferation
**Opn,Spp1**	**.**		**20**	**4**	**Osteopontin**	cytokine	ECM organization, signaling
**Paics**	**.**		**8**	**5**	**p-ribosylamin lase/synthase**	ligase, lyase, binding	metabolism
**Pdcd6ip**	**6**	**3**	**118**	**27**	**Programmed cell death 6-interact.**	binding	TJ & MVB & Exo assembly, protein transport
**Plscr3**	**.**		**5**	**3**	**Phospholipid scramblase 3**	transfer/carrier protein	apoptosis, cholesterol & glucose homeostasis
**Polr2c**	**.**		**10**	**6**	**RNA polymerase II subunit C**	DNA & protein binding	splicing, transcription, signaling
**Prkcd**	**6**	**4**	**222**	**25**	**protein kinase C delta**	transferase, kinase, carrier	multiple signaling pathways
**Psmd1**	**1**	**1**	**12**	**7**	**proteasome 26S, non-ATPase 1**	enzyme modulator	multiple sign.pathways
**Psmd13**	**1**	**1**	**19**	**7**	**proteasome 26S, non-ATPase 13**	enzyme modulator	multiple signaling pathways
**Psmd2**	**7**	**6**	**32**	**19**	**proteasome 26S, non-ATPase 2**	binding, enzyme modulator	multiple signaling pathways
**Psmd3**	**5**	**3**	**24**	**13**	**proteasome 26S, non-ATPase 3**	enzyme modulator	multiple signaling pathways
**Psmd6**	**1**	**1**	**35**	**16**	**proteasome 26S, non-ATPase 6**	regulator	multiple signaling pathways,
**Ruvbl1**	**2**	**1**	**17**	**8**	**RuvB like AAA ATPase 1**	binding, hydrolase	protein metabolism, ß-cat/TCF activation
**Ruvbl2**	**4**	**3**	**20**	**10**	**RuvB like AAA ATPase 2**	protein & ATP binding	DNA repair, transcription activation, protein folding
**Sbno1**	**.**		**2**	**2**	**strawberry notch homolog 1**	molecular function	transcription regulation
**Scimp**	**.**		**6**	**2**	**SLP adapter & CSK-interact. p.**	protein binding	immune synapse formation
**Sdcbp**	**4**	**1**	**68**	**6**	**syndecan binding protein**	binding, traffic regul.	Ephrin signaling
**Sec13**	**.**		**4**	**2**	**SEC13, COPII coat complex**	traffic. reulator	Rho signaling, cell cycle, vesicle transport, membrane traffic.
**Serpinc1**	**.**		**41**	**6**	**serpin family C member 1**	protease inhibitor	clotting cascade
**Srsf1**	**6**	**2**	**67**	**8**	**Serine/arginine-rich splicing F1 1**	RNA & protein binding	splicing, mRNA transport
**Ssrp1**	**.**		**48**	**10**	**structure recognition protein 1**	HMG box TF, signaling	transcription
**Stk24**	**.**		**17**	**6**	**Serine/threonine-protein kinase 24**	kinase, transferase	apoptosis execution
**Sub1**	**1**	**1**	**58**	**8**	**SUB1 transcriptional regulator**	transcription coactiv.	transcription regulation, SMAD signaling
**Supt16h**	**1**	**1**	**7**	**5**	**SPT16 chromatin remodeling**	RNA binding, transcript. cofactor	transcription
**Tnfsf13**	**.**		**4**	**2**	**TNF superfamily member 13**	cytokine	cytokine signaling (TNFR)
**Tspan14**	**1**	**1**	**9**	**5**	**Tetraspanin-14**	adhesion, signaling	plasma membrane organization, NOTCH sign. neutrophil degranulation
**Vps28**	**.**		**11**	**5**	**VPS28, ESCRT-I subunit**	binding	ESCRT, vesicle transport
**Wdr5**	**1**	**1**	**10**	**5**	**WD repeat domain 5**	esterase, protein binding	kinase inhibitor

Table S5B

Higher peptide recoveryn in MDSC than MDSC-Exo

		MDSCinvitro		MDSC in vitro-Exo	
**Synonym.**	**Protein Description**	**Prot.M.**	**Pept.M.**	**Prot.M.**	**Pept.M.**
Acaa1a	acetyl-CoA acyltransferase 1A	20	7		
Acad	Acyl-CoA dehydrogenase	4	9		
Aco2	Aconitate hydratase, mitochondrial	78	20		
Acsl1	Acyl-CoA synthetase long-chain family member 1	30	9		
Actn1	actinin alpha 1	141	35	8	4
Actn4	actinin alpha 4	128	36		
Adpgk	ADP-dependent glucokinase	36	11		
Agps	alkylglycerone phosphate synthase	23	5		
Ak2	Adenylate kinase 2	30	9		
Akr1a1	aldo-keto reductase family 1 member A1	43	11		
Aldh2	aldehyde dehydrogenase 2 family	262	29	7	5
Alox5	Arachidonate 5-lipoxygenase	49	13		
Ankfy1	Ankyrin repeat and FYVE domain containing 1	8	5		
Anp32b	Acidic nuclear phosphoprotein 32 B	30	14	5	3
Ap1b1	AP-1 complex subunit beta-1	33	12		
Api5	Apoptosis inhibitor 5	8	5		
Apmap	Adipocyte plasma membrane-associated protein	8	6		
Arf4	ADP-ribosylation factor 4	38	8		
Arg1	Arginase-1	35	10		
Arhgap1	Rho GTPase-activating protein 1	12	5		
Arl8a	ADP-ribosylation factor-like GTPase 8A	23	8		
Arl8b	ADP-ribosylation factor-like GTPase 8B	37	11	4	3
Arpc2	Actin-related protein 2/3 complex subunit 2	83	15		
Asah1	N-acylsphingosine amidohydrolase 1	12	6		
Atp1a1	ATPase Na+/K+ transporting subunit alpha 1	49	11	3	2
Atp2a2	ATPase Ca2+ transporting 2	72	15		
Atp2a3	ATPase Ca2+ transporting 3	29	6		
Atp5a1	ATP synthase, H+ transporting F1 alpha subunit 1	331	25		
Atp5b	ATP synthase, H+ transporting F1 beta subunit	296	25	8	4
Atp5c1	ATP synthase, H+ transporting F1 gamma subunit	46	6		
Atp5f1	ATP synthase, H+ transporting, F0 subunit B1	30	11		
Atp5h	ATP synthase, H+ transporting, F0 subunit D	21	8		
Atp5i	ATP synthase, H+ transporting, F0 subunit E	12	5		
Atp5o	ATP synthase, H+ transporting, F1 subunit O	42	9		
Atp6v1a	ATPase H+ transporting V1 subunit A	52	16		
Atp6v1b2	ATPase H+ transporting V1 subunit B2	20	5	1	1
B4galnt1	Beta-1,4 N-acetylgalactosaminyltransferase 1	17	12	1	1
Bax	BCL2 associated X, apoptosis regulator	14	6		
Bcap31	B-cell receptor-associated protein 31	10	5		
Blvrb	biliverdin reductase B	10	5		
Bzw1	Basic leucine zipper and W2 domain 1	14	8		
Ca2	Carbonic anhydrase 2	21	7	2	1
Calr	Calreticulin	159	22		
Canx	Calnexin	87	19		
Capn1	Calpain-1	29	18	1	1
Cat	Catalase	30	15		
CD166	CD166 antigen, Alcam	24	12	2	1
CD177	CD177 antigen	39	13	3	2
CD180	CD180 antigen	15	8		
CD206	mannose receptor C-type 1	48	27	1	1
CD29	Integrin beta-1	27	9	6	3
CD41	Integrin alpha-IIb	23	12	2	1
CD61	Integrin beta-3	14	6		
CD74	CD74 antigen, invariant chain associated	23	5	3	1
Celf2	CUGBP Elav-like family member 2	10	5		
Chil3	Chitinase-like 3	63	13	5	2
Ckap4	Cytoskeleton-associated protein 4	54	20		
Cmpk2	cytidine/uridine monophosphate kinase 2	37	12		
Cndp2	CNDP dipeptidase 2	77	16	6	3

Table S5B cont.

Synonym.	Protein Description	MDSCinvitro Prot.M.	MDSCinvitro Pept.M.	MDSC in vitro-Exo Prot.M.	MDSC in vitro-Exo Pept.M.
Colgalt1	collagen beta(1-O)galactosyltransferase 1	6	5		
Copz1	Coatomer protein complex subunit zeta 1	11	7		
Cox4i1	cytochrome c oxidase subunit IV isoform 1	34	9		
Cox5a	cytochrome c oxidase subunit 5A	12	5		
Cox6c	Cytochrome c oxidase subunit 6C	11	5		
Cs	Citrate synthase	47	11	2	2
Ctsa	cathepsin A	15	7		
Ctsc	cathepsin C	20	6	4	2
Ctsg	Cathepsin G	23	8	1	1
Ctsz	Cathepsin Z	32	7	2	1
Cyb5r3	cytochrome b5 reductase 3	33	11		
Cybb	Cytochrome b-245 beta chain	50	13		
Cycs	Cytochrome c, somatic	19	7		
Dars	Aspartate--tRNA synthase	14	8		
Ddost	dolichyl-diphosphooligosacch glycosyltransfer.	53	11	6	3
Ddx39a	DExD-box helicase 39A	80	14	1	1
Ddx39b	DExD-box helicase 39B	64	6		
Ddx3x	DEAD-box helicase 3, X-linked	17	7		
Ddx5	DEAD-box helicase 5	50	12	4	2
Dhx9	DEAH-box helicase 9	30	11	4	3
Diaph1	diaphanous related formin 1	16	8		
Dlat	dihydrolipoamide S-acetyltransferase	10	5		
Dld	dihydrolipoamide dehydrogenase	27	10		
Dlst	dihydrolipoamide S-succinyltransferase	27	6		
Dnajb11	DnaJ heat shock protein family B11	12	6	3	2
Dnajc3	DnaJ heat shock protein family C3	9	6		
Dock8	Dedicator of cytokinesis 8	7	5		
Dpysl2	Dihydropyrimidinase like 2	18	11		
Dstn	Destrin	46	6	2	2
Ear2	eosinophil-associated, ribonuclease A2	22	7	4	2
Echs1	Enoyl-CoA hydratase, short chain 1	14	8		
Eef1b	Elongation factor 1-beta	301	15	1	1
Eef1d	Elongation factor 1-delta	19	6	2	2
Eef1g	Elongation factor 1-gamma	152	12	8	4
Eef2	Elongation factor 2	269	35	29	8
Ehd1	EH domain-containing 1	29	13	6	2
Ehd4	EH domain-containing 4	45	12	8	4
Eif1	Eukaryotic translation initiation factor 1	10	6		
Eif4a3	Eukaryotic initiation factor 4A3	27	6		
Eif5a	Eukaryotic translation initiation factor 5A	51	14	4	1
Elane	elastase, neutrophil expressed	26	5		
Elavl1	ELAV like RNA binding protein 1	13	6		
Emb	Embigin	22	6	1	1
Entpd1	Ectonucleos. triphosphate diphosphohydrolase 1	7	5		
Erap1	Endoplasmic reticulum aminopeptidase 1	32	5		
Ero1a	endoplasmic reticulum oxidoreductase 1 alpha	79	12		
Erp29	Endoplasmic reticulum protein 29	37	9	3	2
Erp44	Endoplasmic reticulum protein 44	23	8		
Esyt1	Extended synaptotagmin-1	217	37	22	9
Etfa	Electron transfer flavoprotein alpha subunit	30	8	2	1
Etfb	Electron transfer flavoprotein beta subunit	19	8		
Fabp5	Fatty acid-binding protein 5	75	15	1	1
Fam129b	family with sequence similarity 129 member B	29	9		
Fam162a	family with sequence similarity 162 member A	13	5		
Fam49b	family with sequence similarity 49 member B	23	9	3	2
Fh	Fumarate hydratase	16	10		
Fis1	fission, mitochondrial 1	14	5		
Fkbp2	FK506 binding protein 2	11	5		
Fkbp3	FK506 binding protein 3	8	5		

Table S5B cont.

Synonym.	Protein Description	MDSCinvitro Prot.M.	MDSCinvitro Pept.M.	MDSC in vitro-Exo Prot.M.	MDSC in vitro-Exo Pept.M.
Flna	Filamin-A	391	71	27	16
G3bp1	GTPase activating protein binding protein 1	12	5		
Ganab	glucosidase II alpha subunit	113	26		
Gba	Glucosylceramidase beta	10	6		
Glb1	galactosidase, beta 1	8	6		
Glg1	Golgi apparatus protein 1	32	10	5	3
Glo1	glyoxalase I	12	6		
Glud1	Glutamate dehydrogenase 1	77	21		
Gmfg	Glia maturation factor gamma	13	6		
Gnai2	guanine nucleotide binding protein alpha inhibit 2	140	15	19	5
Gnb1	guanine nucleotide binding protein beta 1	28	8		
Gnb2	guanine nucleotide binding protein beta 2	52	12	3	2
Got2	Aspartate aminotransferase, mitochondrial	93	19		
Gpd2	Glycerol-3-phosphate dehydrogenase 2	30	9		
Gpi	Glucose-6-phosphate isomerase	113	21	5	3
Gpx1	Glutathione peroxidase 1	9	5		
Grb2	Growth factor receptor-bound protein 2	13	7	2	2
Gsr	Glutathione disulfide reductase	16	5		
Gstm1	Glutathione S-transferase Mu 1	26	12	1	1
Gsto1	Glutathione S-transferase omega-1	13	6		
H2-D1	histocompatibility 2, D region locus 1	132	11	7	4
H2-Eb1	histocompatibility 2, class II antigen E beta	18	6		
Hadha	hydroxyacyl-CoA dehydrogenase alpha subunit	33	12		
Hadhb	hydroxyacyl-CoA dehydrogenase beta subunit	25	9		
Hars	histidyl-tRNA synthetase	10	6		
Hdac1	Histone deacetylase 1	9	6		
Hexa	Hexosaminidase subunit alpha	14	8		
Hexb	Hexosaminidase subunit beta	18	11		
Hk1	Hexokinase-1	74	15		
Hk2	Hexokinase-2	53	12		
Hk3	Hexokinase-3	45	13		
Hmox1	Heme oxygenase 1	26	7		
Hmox2	Heme oxygenase 2	27	10		
Hnrnpa1	Heterogeneous ribonucleoprotein A1	95	15	2	1
Hnrnpa2b1	Heterogeneous ribonucleoproteins A2/B1	82	17	2	1
Hnrnpa3	Heterogeneous ribonucleoprotein A3	94	11		
Hnrnpab	Heterogeneous ribonucleoprotein A/B	33	6	1	1
Hnrnpd	Heterogeneous ribonucleoprotein D0	34	8	2	1
Hnrnpf	Heterogeneous ribonucleoprotein F	23	6	4	2
Hnrnph1	Heterogeneous ribonucleoprotein H1	67	13		
Hnrnpk	Heterogeneous ribonucleoprotein K	105	12	14	3
Hnrnpl	Heterogeneous ribonucleoprotein L	31	11	1	1
Hnrnpm	Heterogeneous ribonucleoprotein M	24	6		
Hpcal1	Hippocalcin-like 1	9	5		
Hprt1	Hypoxanthine phosphoribosyltransferase 1	9	5		
Hsd11b1	hydroxysteroid 11-beta dehydrogenase 1	24	5		
Hsd17b11	hydroxysteroid 17-beta dehydrogenase 11	28	6	2	1
Hsd17b12	hydroxysteroid 17-beta dehydrogenase 12	31	6		
Hsd17b4	hydroxysteroid 17-beta dehydrogenase 4	9	5		
Hsp90aa1	Heat shock protein HSP 90-alpha	255	27		
Hsp90b1	heat shock protein 90, beta 1	457	35	20	4
Hspa1a	Heat shock 70 kDa protein 1A	31	7		
Hspa5	heat shock protein 5	544	38	43	11
Hspd1	heat shock protein family D (Hsp60) member 1	69	24		
Hspe1	heat shock protein family E (Hsp10) member 1	16	6		
Hyou1	Hypoxia up-regulated protein 1	169	30		
Idh2	Isocitrate dehydrogenase [NADP] 2	63	14		
Idh3a	Isocitrate dehydrogenase [NAD] alpha	41	9		
Ifi44	Interferon-induced protein 44	27	9		

Table S5B cont.

Synonym.	Protein Description	MDSCinvitro Prot.M.	Pept.M.	MDSC in vitro-Exo Prot.M.	Pept.M.
Ifit1	Interferon-induced protein 1	40	12		
Ifit2	Interferon-induced protein 2	19	6		
Immt	inner membrane mitochondrial protein	7	5		
Kctd12	potassium channel 12	28	7	4	2
Kpnb1	karyopherin subunit beta 1	55	13	1	1
Lap3	leucine aminopeptidase 3	19	12		
Lbr	Lamin-B receptor	38	8		
Lcp1	lymphocyte cytosolic protein 1	486	38	20	9
Lgals1	Galectin-1	30	8	1	1
Lgals9	Galectin-9	14	5		
Lmnb1	Lamin-B1 Lmnb1	21	10		
Lnpep	Leucyl-cystinyl aminopeptidase	37	15		
Lpcat2	Lysophosphatidylcholine acyltransferase 2	17	5		
Lrpap1	LDL receptor related protein associated protein 1	26	10		
Lrpprc	leucine rich pentatricopeptide repeat containing	11	5		
Lrrc59	Leucine-rich repeat-containing protein 59	43	9		
Lta4h	Leukotriene A-4 hydrolase	64	13		
Man2b1	mannosidase alpha class 2B member 1	23	10	1	1
Mapre1	Microtubule-associated protein RP/EB member 1	11	5		
Mcm2	minichromosome maintenance complex 2	7	5		
Mdh1	Malate dehydrogenase 1	16	9	5	3
Mdh2	Malate dehydrogenase 2	114	16	11	5
Me2	Malic enzyme 2	17	8		
Mogs	Mannosyl-oligosaccharide glucosidase	20	6		
Mta2	metastasis associated 1 family member 2	10	6		
Mthfd1l	methylenetetrahydrofolate dehydrogenase 1-like	14	6		
Mybbp1a	Myb-binding protein 1A	29	12	10	3
Naga	Alpha-N-acetylgalactosaminidase	9	6		
Naa50	N-alpha-acetyltransferase 50	16	8		
Napa	NSF attachment protein alpha	16	8	3	2
Nceh1	Neutral cholesterol ester hydrolase 1	17	6		
Ncf1	Neutrophil cytosol factor 1	41	10		
Ncf2	Neutrophil cytosol factor 2	24	10	3	2
Ncf4	Neutrophil cytosol factor 4	18	9		
Ncstn	Nicastrin	17	7	3	2
Ndufa13	NADH:ubiquinone oxidoreductase A13	17	5		
Ndufa8	NADH:ubiquinone oxidoreductase A8	13	6		
Ndufb10	NADH:ubiquinone oxidoreductase B10	10	7		
Ndufs3	NADH:ubiquinone oxidoreductase S3	7	5		
Nme1	NME/NM23 nucleoside diphosphate kinase 1	49	9		
NOMO1	Nodal modulator 1	14	7		
Nucb1	Nucleobindin-1	10	7		
Nudt21	nudix hydrolase 21	13	7		
Oas1a	2'-5'-oligoadenylate synthase 1A	47	11		
Oat	Ornithine aminotransferase	16	9		
Ogdh	oxoglutarate dehydrogenase	38	15		
Osbpl8	Oxysterol-binding protein like 8	20	7	6	2
Ostf1	Osteoclast-stimulating factor 1	9	6		
Oxct1	3-oxoacid CoA-transferase 1	19	6		
P4hb	prolyl 4-hydroxylase subunit beta	286	31		
Pai2	Plasminogen activator inhibitor 2	19	10		
Pcbp2	Poly(rC)-binding protein 2	52	6		
Pdcd10	Programmed cell death 10	9	5		
Pdia3	Protein disulfide-isomerase A3	263	37	3	2
Pdia4	Protein disulfide-isomerase A4	182	32		
Pdia6	Protein disulfide-isomerase A6	113	15	3	2
Phb	Prohibitin	36	8		
Phb2	Prohibitin-2	34	13		
Pitpna	Phosphatidylinositol transfer protein alpha	9	7		

Table S5B cont.

Synonym.	Protein Description	MDSCinvitro Prot.M.	Pept.M.	MDSC in vitro-Exo Prot.M.	Pept.M.
Pkm	Pyruvate kinase PKM	403	31		
Plbd1	phospholipase B domain containing 1	15	7	2	1
Pld4	Phospholipase D4	19	7		
Plec	Plectin	33	9		
Plek	Pleckstrin	14	5	1	1
Pnkp	polynucleotide kinase 3'-phosphatase	8	5		
Pnp	Purine nucleoside phosphorylase	65	11		
Por	cytochrome P450 oxidoreductase	67	14		
Ppa1	pyrophosphatase 1	8	5		
Ppif	Peptidyl-prolylisomerase F	8	5		
Ppp1ca	protein phosphatase 1 catalytic subunit alpha	36	13	2	2
Ppp1cb	protein phosphatase 1 catalytic subunit beta	19	9		
Ppp1cc	protein phosphatase 1 catalytic subunit gamma	25	11		
Ppp2ca	protein phosphatase 2 alpha	12	7	5	2
Ppp2r1a	Protein phosphatase 2A subunit A	15	7		
Prdx2	Peroxiredoxin-2	15	5	1	1
Prdx3	Peroxiredoxin-3	45	5		
Prdx4	Peroxiredoxin-4	50	5		
Prdx5	Peroxiredoxin-5	67	12	4	3
Prdx6	Peroxiredoxin-6	30	13		
Prtn3	proteinase 3	24	6		
Psma4	Proteasome subunit alpha 4	18	6		
Psme1	proteasome activator subunit 1	38	11		
Ptbp3	Polypyrimidine tract-binding protein 3	19	8		
Ptgr1	Prostaglandin reductase 1	7	5		
Ptgs1	prostaglandin-endoperoxide synthase 1	22	8		
Ptpn1	protein tyrosine phosphatase, non-receptor type 1	48	9	2	1
Ptprc	protein tyrosine phosphatase, receptor type C	85	17	1	1
Pycard	PYD and CARD domain containing	8	5		
Pygl	Glycogen phosphorylase L	106	22	14	7
Rab18	RAB18, member RAS oncogene family	13	6		
Rab21	RAB21, member RAS oncogene family	21	8		
Rab27a	RAB27A, member RAS oncogene family	10	5		
Rab2a	RAB2A, member RAS oncogene family	39	9	5	2
Rab31	RAB31, member RAS oncogene family	11	5		
Rab5a	RAB5A, member RAS oncogene family	20	6		
Ralb	RAS like proto-oncogene B	11	5		
Rap2c	RAP2C, member of RAS oncogene family	13	6		
Rbbp4	RB binding protein 4	16	5	1	1
Rbbp7	RB binding protein 7	23	5	2	1
Rbmxl1	RNA binding motif protein, X-linked-like-1	22	5		
Rnf213	ring finger protein 213	8	6		
Rnh1	ribonuclease/angiogenin inhibitor 1	52	14	6	4
Rpl13	ribosomal protein L13	22	8	8	2
Rpl13a	ribosomal protein L13a	18	5		
Rpl15	ribosomal protein L15	12	5	2	1
Rpl17	ribosomal protein L17	24	5	2	1
Rpl18a	ribosomal protein L18a	30	6		
Rpl4	ribosomal protein L4	79	12	5	2
Rpl7	ribosomal protein L7	31	13	7	1
Rpn1	ribophorin I	169	21		
Rpn2	ribophorin II	95	15		
Rps14	ribosomal protein S14	33	7		
Rps16	ribosomal protein S16	50	8	14	2
Rps17	ribosomal protein S17	21	7		
Rps9	ribosomal protein S9	38	12		
Rrbp1	Ribosome-binding protein 1	46	19		
Samhd1	SAM and HD domain cont. triphosphohydrolase 1	49	16	1	1
Sars	seryl-tRNA synthetase	10	5		

Table S5B cont.

Synonym.	Protein Description	MDSCinvitro Prot.M.	MDSCinvitro Pept.M.	MDSC in vitro-Exo Prot.M.	MDSC in vitro-Exo Pept.M.
Scp2	sterol carrier protein 2	20	10		
Sdf2l1	Stromal cell-derived factor 2-like 1	20	6	1	1
Sdha	succinate dehydrogenase complex flavoprotein A	19	6		
Sec11c	SEC11 homolog C, signal peptidase complex	9	5	1	1
Sec22b	SEC22 homolog B, vesicle trafficking protein	12	5		
Sec61a1	Sec61 translocon alpha 1	12	5		
Sept11	Septin-11	23	8		
Serpinb1a	Leukocyte elastase inhibitor A	156	19	3	2
Sfpq	Splicing factor, proline- and glutamine-rich	19	7		
Sfxn1	Sideroflexin-1	15	6		
Sgpl1	Sphingosine-1-phosphate lyase 1	17	6		
Sh3bgrl	SH3 domain binding glutamate rich protein like	9	5		
Siglec1	sialic acid binding Ig-like lectin 1, sialoadhesin	6	5		
Slc25a11	solute carrier family 25 member 11	33	6		
Slc25a12	solute carrier family 25 member 12	40	10		
Slc25a3	solute carrier family 25 member 3	93	11	2	1
Slc25a4	solute carrier family 25 member 4	109	13		
Slc25a5	solute carrier family 25 member 5	204	15	9	4
Slc3a2	solute carrier family 3 member 2	91	16	11	5
Snd1	Staphylococcal nuclease & tudor domain 1	51	21	1	1
Snx2	Sorting nexin-2	12	6		
Sod2	Superoxide dismutase 2	11	5		
Spcs2	Signal peptidase complex subunit 2	16	5	2	1
Sptan1	Spectrin alpha, non-erythrocytic 1	7	5		
Ssr4	signal sequence receptor subunit 4	29	5	1	1
Stat1	Signal transducer and activator of transcription 1	27	7		
Stfa1	Stefin A1	17	6		
Stfa3	Stefin A3	13	6		
Stip1	Stress-induced-phosphoprotein 1	13	6		
Stt3a	STT3A, oligosaccharyltransferase complex	18	6		
Stx7	Syntaxin-7	17	6	4	2
Sun2	SUN domain-containing protein 2	125	20		
Surf4	Surfeit 4	24	5		
Syncrip	synaptotagmin binding protein	19	9		
Tagln2	Transgelin-2	66	12	4	2
Taldo1	Transaldolase 1	52	15	8	3
Tap1	transporter 1, ATP binding cassette subfamily B	16	6		
Tap2	transporter 2, ATP binding cassette subfamily B	21	8		
Tapbp	TAP binding protein	66	11	4	1
Tfrc	Transferrin receptor	30	9		
Tgm2	transglutaminase 2	21	6		
Tkt	Transketolase	163	20	1	1
Tln1	Talin-1	297	57	24	14
Tln2	Talin-2	26	6		
Tmem43	Transmembrane protein 43	15	9	1	1
Tmpo	thymopoietin	12	6		
Tor1aip1	Torsin-1A-interacting protein 1	23	9		
Tpi1	Triosephosphate isomerase	43	10	6	3
Tpr	translocated promoter region	14	9		
Trap1	TNF receptor associated protein 1	20	5		
Trex1	Three-prime repair exonuclease 1	14	5		
Tubb2a	Tubulin beta-2A	166	15		
Txn	Thioredoxin	8	5		
Txndc5	Thioredoxin domain containing 5	11	6		
U2af2	U2 small nuclear RNA auxiliary factor 2	9	5	1	1
Ube2n	Ubiquitin-conjugating enzyme E2 N	15	5		
Ube2v1	Ubiquitin-conjugating enzyme E2 variant 1	8	5		
Uggt1	UDP-glucose glycoprotein glucosyltransferase 1	116	39		
Ugt1a7c	UDP glucuronosyltransferase 1 polypeptide A7C	35	9		

Table S5B cont.

Synonym.	Protein Description	MDSCinvitro Prot.M.	MDSCinvitro Pept.M.	MDSC in vitro-Exo Prot.M.	MDSC in vitro-Exo Pept.M.
Uqcrc2	ubiquinol-cytochrome c reductase core protein II	22	11		
Uso1	USO1 vesicle transport factor	17	6		
Vamp8	Vesicle-associated membrane protein 8	12	5	2	1
Vapa	VAMP associated protein A	21	5		
Vars	valyl-tRNA synthetase	15	7		
Vcl	Vinculin	27	14		
Vdac1	Voltage-dependent anion channel 1	64	15	2	2
Vdac2	Voltage-dependent anion channel 2	63	11		
Vdac3	Voltage-dependent anion channel 3	40	7		
Vim	Vimentin	371	42	20	7
Vps35	vacuolar protein sorting 35 homolog	49	13	6	2
Vwa5a	von Willebrand factor A domain 5A	24	12		
Wdr1	WD repeat domain 1	57	14	1	1
Xpo1	Exportin-1	11	5		
Ywhaq	14-3-3 protein theta	48	6		

Table S6

MDSC-Exo peptides recovered in pulldown from Sepharose-coupled lymph node cell membranes[a]

		MDSC-Exo over LNC membrane		cellular localization
**Synonym**	**Protein Description**	**Prot.M.**	**Pept.M**	
Acaa2	acetyl-CoA acyltransferase 2	14	9	mitochondrion
Acadvl	acyl-CoA dehydrogenase, very long chain	21	12	mitochondrial inner membr.
Aco2	Aconitate hydratase, mitochondrial	8	5	mitochondrion, myelin sheet
Acta2	actin, alpha 2, smooth muscle, aorta	68	20	cytoskeleton, cytoplasm
Aldh2	aldehyde dehydrogenase 2 family	15	9	mitochondrion
Ank1	Ankyrin-1	6	5	cytoskel., plasma membrane
Anxa11	Annexin A11	9	5	cytoskel., membrane
Anxa4	Annexin A4	8	5	plasma membrane
Arhgdib	Rho GDP-dissociation inhibitor beta	7	6	cytoplasm, membrane
Arhgef1	Rho guanine nucleotide exchange factor 1	6	5	cytoplasm, plasma membrane
Atp1a1	ATPase Na+/K+ transporting subunit alpha 1	22	11	ER & plasma membrane, raft
Atp2a1	ATPase ER Ca2+ transporting 1	27	13	ER membrane
Atp2a3	ATPase ER Ca2+ transporting 3	14	8	ER membrane
Cap1	CAP, adenylate cyclase-associated protein 1	17	8	cytoskeleton, raft
Capg	capping actin protein, gelsolin like	9	5	nucleus, cytoplasm
CD11b	Integrin alpha-M	7	5	plasma membrane
Copg1	coatomer protein complex subunit gamma 1	6	5	cytoplasmic vesicle coat
Cyc1	Cytochrome c1	10	5	nucleus, mitoch. inner membr.
Ddx39b	DExD-box helicase 39B	9	5	nucleus, cytopl.,spliceosome
Ddx5	DEAD-box helicase 5	11	7	nucleus, cytopl.,spliceosome
Dync1h1	dynein cytoplasmic 1 heavy chain 1	8	6	cytopl., cytoskel. (complex)
Eftud2	elongation factor Tu GTP binding domain containing 2	28	15	nucleus, spliceosome compl.
Eif3b	eukaryotic translation initiation factor 3 subunit B	9	5	cytoplasm (complex)
Eif4a2	Eukaryotic initiation factor 4A-II Eif4a2	13	6	nucleolus, cytoplasm
Eno1	enolase 1	17	8	raft, synaptic membrane
Etfa	electron transfer flavoprotein alpha subunit	10	6	mitochondrion (complex)
Etfdh	electron transfer flavoprotein dehydrogenase	8	5	mitochondrial membrane
Fgg	Fibrinogen gamma chain	7	5	cytopl,plasm.membr. (extern.)
Flna	Filamin-A	17	9	nucleus, cytosol, pl.membrane
Gimap4	GTPase IMAP family member 4	9	6	cytoplasm
Gpd2	glycerol phosphate dehydrogenase 2, mitochondrial	20	11	membrane (mitochondr.)
H2afx	H2A histone family member X	20	6	nucleus
H2afy	H2A histone family member Y	15	7	nucleus
H2-D1	histocompatibility 2, D region locus 1	18	8	ER, plasma membr., raft
Hadha	hydroxyacyl-CoA dehydrogenase, alpha subunit	43	20	mitochondrion (complex)
Hadhb	hydroxyacyl-CoA dehydrogenase, beta subunit	12	7	mitochondrial membrane
Hist1h1c	histone cluster 1 H1 family member c	13	6	nucleus
Hist1h1e	histone cluster 1 H1 family member e	11	5	nucleosome
Hist1h2bb	histone cluster 1 H2B family member b	84	5	nucleus, cytoplasm
Hnrnpk	heterogeneous nuclear ribonucleoprotein K	11	5	nucleus, spliceosome compl.
Hnrnpu	heterogeneous nuclear ribonucleoprotein U	15	7	mitotic spindle, spliceosome
Hsp90ab1	heat shock protein 90 alpha, class B member 1	30	16	plasma & vesicle membrane
Idh3a	isocitrate dehydrogenase 3 (NAD+) alpha	17	7	mitochondrion, myelin sheet
Krt8	Keratin 8	10	6	cytoskel., plasma membrane
Kpnb1	karyopherin (importin) beta 1	11	6	nuclear membr., ER network
Lman1	lectin, mannose binding 1	8	5	organelles (membrane bound)
Lmnb1	Lamin-B1 Lmnb1	10	6	nucl. membr., intermed.filam.
Msn	Moesin	7	5	plasma membrane (cytopl)
Mtco2	Cytochrome c oxidase subunit 2 Mtco2	16	4	mitochondrial inner membr.
Myh14	Myosin-14	14	7	stress fibers (complex)
Myh4	Myosin-4	156	72	cytoplasm, myosin filament

Table S6 cont.

Synonym	Protein Description	MDSC-Exo over LNC membrane Prot.M.	Pept.M	cellular localization
Myh9	Myosin-9	161	70	pl.membr.,signalos.,imm.synapse
Myl1	Myosin light chain 1/3, skeletal muscle isoform	21	8	myosin complex (fibres)
Mylpf	Myosin regulatory light chain 2, skeletal muscle isoform	10	6	lysosome complex
Ndufs1	NADH-ubiquinone oxidoreductase 75 kDa subunit	8	5	mitoch.inner membrane
Ogdh	oxoglutarate dehydrogenase	8	5	mitochondrion (complex)
Pcbp1	Poly(rC)-binding protein 1	14	7	nucleus, ribosome complex
Pcbp2	Poly(rC)-binding protein 2	9	5	nucleus, cytopl., membrane
Pdia6	protein disulfide isomerase family A member 6	6	5	ER (complex), pl.membr.
Pfn1	Profilin-1	5	3	cytoskeleton, membrane
Phgdh	phosphoglycerate dehydrogenase	7	5	myelin sheath
Pkm	Pyruvate kinase, muscle	19	10	cytopl.,mitochondr. (complex)
Pml	promyelocytic leukemia	8	5	nucleus, cytosol, ER membr.
Psmd1	proteasome 26S subunit, non-ATPase 1	9	6	proteasome complex
Psmd13	proteasome 26S subunit, non-ATPase 13	14	7	proteasome complex
Psmd2	proteasome 26S subunit, non-ATPase 2	9	6	proteasome complex
Psmd6	proteasome 26S subunit, non-ATPase 6	17	9	proteasome complex
Psme1	Proteasome activator subunit 1	9	5	proteasome complex
Ptprc	protein tyrosine phosphatase, receptor type C	6	5	plasma membr. (outer side)
Rack1	receptor for activated C kinase 1	14	7	cytopl., ribosome, pl.membr.
Rpl12	ribosomal protein L12	9	5	ribosome complex
Rpn1	ribophorin I	9	6	ER membrane
Sdha	succinate dehydrogenase complex flavoprotein subunit A	16	8	mitoch.inner membr.(complex)
Sf3b3	Splicing factor 3B subunit 3	15	8	spliceosome complex
Slc25a11	solute carrier family 25 member 11	4	3	mitochondrial inner membrane
Slc25a12	solute carrier family 25 member 12	18	11	mitochondr.inner membrane
Slc25a4	solute carrier family 25 member 4	15	8	mitoch.membr., cytoskel., raft
Sptb	spectrin beta, erythrocytic	71	32	cytoskel.,pl.membr., (complex)
Stat1	Signal transducer and activator of transcription 1	12	7	nucleus, cytoplasm (complex)
Tap2	transporter 2, ATP binding cassette subfamily B member	7	5	ER membrane (complex)
Tfrc	Transferrin receptor	15	9	endosome & plasma membr.
Tgtp1	T cell specific GTPase 1	10	4	ER membrane
Tln1	Talin-1	38	19	cytoskel.,plasma membrane
Tmed10	transmembrane p24 trafficking protein 10	9	5	organelle (membr., compl.)
Ucp1	uncoupling protein 1 (mitochondrial, proton carrier)	10	7	mitochondr.inner membrane
Vcp	valosin containing protein	13	9	proteas.compl., ER membr.
Vdac1	voltage-dependent anion channel 1	20	9	mitochonr. & plasma membrane
Vdac2	voltage-dependent anion channel 2	12	7	mitochonr. & plasma membr., raft
Vdac3	voltage-dependent anion channel 3	11	5	mitochondrial membrane
Vim	Vimentin	12	6	cytoskel., plasma membrane
Xpo1	Exportin-1	10	*5*	cytopl., organelle membr.

[a] proteins with ≥3 peptide hits are shown

Table S7

***In vivo* impact of MDSC-Exo on AA spleen cells**

Table S7A

Increased mRNA recovery in spleen cells of MDSC-Exo treated AA mice

**Synonym**	**AA SC**	**Exo-treated**	**Protein name**	**function**	**biological processes**[a]
Abca1	1595	10791	ATP-binding cassette sub-family A member 1	transport, lipoprotein biosynthesis, LPS response	lipoprotein metab, endosomal transport, phagocytosis
Abcg1	1370	5488	ATP-binding cassette sub-family G member 1	transport, lipoprotein biosynthesis	lipoprotein metabolism, ABC family transport
Adgre1	1664	7722	Adhesion G protein-coupled receptor E1	signal transduction, GPCR signaling	IR regulation
Agap1	316	1005	Arf-GAP with GTPase,ANK repeat & PH domain prot.1	ARP3 complex regulation	membrane traffic, cytoskeleton organization
Aldh1l2	238	1042	Mitochondrial 10-formyltetrahydrofolate dehydrogenase	oxidation-reduction	vitamin metabolism
Als2cl	3427	12879	ALS2 C-terminal-like protein	GTPase	endosome organization
Angptl2	301	1478	Angiopoietin-related protein 2	endothelial growth factor	organism development
Ankrd55	270	1993	Ankyrin repeat domain-containing protein 55	protein-protein interaction	suggested involvement in autoimmune diseases
Arg1	14325	57553	Arginase-1	hydrolase, ion binding	AA metabolism, T proliferation↓, IFNγ signaling↓
Atf3	1102	3326	Cyclic AMP-dependent transcription factor ATF-3	transcription, gluconeogenesis	TRAIL-activation, apoptotic signaling
Atf5	2046	6599	Cyclic AMP-dendent transcription factor ATF-5	chromatin binding, transcription factor	cell cycle regulation, proliferation↓, apoptosis↓
Atn1	3185	9859	Atrophin-1	transcription corepressor	CNS development, neuron apoptosis
Atp6v0d2	1684	6401	V-type proton ATPase subunit d2	ion transport	ROS/RNS production, endocytosis, transport
C3ar1	1476	8124	C3a anaphylatoxin chemotactic receptor	C3a binding, GPCR activ.	chemotaxis, inflamm.IR, C' cascade, GPCR signaling
Cacna1d	329	1108	Voltage-dependent L-type calcium channel alpha-1D	ion chanel, ankyrin binding	transmembrane transport
Cacnb1	284	1131	Voltage-dependent L-type calcium channel beta-1	calcium transport	transmembrane transport, synaptic transmission
Ccl2	1445	8996	C-C motif chemokine 2	cytokine activity, CCR2 binding, chemotaxis	IL1, IFNγ, TNF, TGFß, GPCR signaling
Ccl7	262	2216	C-C motif chemokine 7	cytokine activity, CCR2 binding, chemotaxis	GPCR signaling, IL1, IFNγ, TNF response
Ccnd1	299	1254	G1/S-specific cyclin D1	transcription	cell cycle regul., Wnt signaling, DNA damage response
Ccr9	420	1790	C-C chemokine receptor type 9	chemotaxis, chemokine / GPCR signaling	IR regulation
Cd200	820	3180	OX-2 membrane protein	protein binding, adhesion	IR (lymphoid-nonlymphoid) regulation
Cd302	473	1423	CD302 antigen	C-type lectin transmembrane receptor	myeloid DC migration, phagocytosis
Cd36	2234	11144	Platelet glycoprotein 4	cytokine secretion	antigen cross-present.,vesicl.-med.transport, TLR sign.
Cd83	2025	11032	CD83 antigen	humoral IR	CD4 different., IL2 & IL10 production
Cd86	656	2229	T-lymphocyte activation antigen	TLR & PI3K & BCR & FGFR signaling	CD28-dependent T cell stimulation
Cdk18	422	1483	Cyclin-dependent kinase 18	transferase	phosphorylation
Cdkn2a	279	1003	Tumor suppressor ARF	cell cycle regulation	cell growth (T & B cells) ↓, NFκB signaling↓
Ch25h	123	1868	Cholesterol 25-hydroxylase	oxidation-reduction	lipid & lipoprotein metabolism, B cell chemotaxis
Ckb	295	1416	Creatine kinase B-type	kinase activity, IL6, IL12, TNF production ↓,	innate IR↓, Myd88/TLR, NFκB, IL1 signaling ↓
Clec4a1	239	1181	C-type lectin domain family4 member a1	dendritic cell inhibitory receptor, inflamm. monocytes	
Clec4a2	361	1233	C-type lectin domain family4 member a2	carbohydrate binding	innate & adaptive IR
Clec4d	1784	6243	C-type lectin domain family4 member d	Ig receptor binding	FcγR signaling, IR regulation
Clmp	308	1901	CXADR-like membrane protein	cell-cell adhesion	
Colec12	213	1060	Collectin-12	pattern recognition receptor	phagocytosis, TLR signaling
Csf1r	3625	10806	Macrophage colony stimulating factor 1 receptor	RTK, cytokine binding, migration	RTK, STAT, ERK1/2 signaling, innate IR, hemopoiesis
Ctsk	509	8274	Catheppsin K	peptidase, proteolysis	TLR cascades
Ctsl	29630	225943	Catheppsin L1	peptidase, histone binding	ECM organiz, MHCII antig.present., hair follicle morph..
Cxcl1	265	1346	Growth-regulated alpha protein	chemokine activ, CXCR binding	chemotaxis regulation, inflamm. IR, GPCR signaling
Cxcl16	1035	3221	C-X-C motif chemokine 16	chemokine & cytokine activity, CXCR binding	T cell chemotaxis, TNF, IFNγ, GPCR signaling
Cxcl2	1539	5103	C-X-C motif chemokine 2	chemokine & cytokine activity, CXCR binding	chemotaxis, inflammatory IR, GPCR signaling

Table S7A cont.

Synonym	AA SC	Exo-treated	Protein name	function	biological processes
Cxcl3	1929	10842	C-X-C motif chemokine 3	chemokine & cytokine activity, CXCR binding	chemotaxis, inflammatory IR, GPCR signaling
Cxcl5	247	2461	C-X-C motif chemokine 5	chemokine activity, CXCR binding, chemotaxis	cytokine production, inflamm.IR, GPCR, JAK/Stat sign.
Dab2	1152	3841	Disabled homolog 2	AP2 complex binding, clathrin adaptive activity	myeloid cell diff., endocytosis, vesicle-med. transport
Dtx1	476	1729	E3 ubiquitin-prootein ligase DTX1	ion binding, transferase	T cell differentiation↓, NOTCH signaling
Dusp10	1832	8026	Dual specificity protein phosphatase 10	MAPK, JNK cascade↓	Treg differentiation, innate IR regulation
Ebf1	342	1128	Transcription factor COE1	transcription	adipocyte differentiation
Ephx1	783	2807	Epoxide hydrolase 1	hydrolase, catalytic activity	response to toxic substances
Fam19a3	1563	5337	Protein FAM19A3	C-C chemokine family	microglia cell activation
Fam20c	943	3954	Extracellular serine/threonine protein kinase FAM20C	protein serine/threonine kinase	muscle development
Flrt2	617	3655	Leucine-rich repeat transmembrane protein FLRT2	protein kinase inhibitor	adhesion, migration, FGFR1 signaling
Foxp3	544	2328	Forkhead box protein P3	transcription factor, IL2, IL5, IL10, IFNγ production	T cell prolif.↓,chronic inflammation, Treg differentiation
Fpr1	809	2453	fMet-Leu-Phe receptor	chemotaxis, leukocyte migration	GPCR signaling
Gja1	307	1080	Gap junction alpha-1 protein	channel activity, signaling	Junction assembly, membr.trafficking, vesicle- transport
Gm15056	80	1496	Protein Gm15056	beta defensin gene family	
Gpnmb	22678	105624	Transmembrane glycoprotein nMB	receptor-ligand activity, TNF production	T cell activation ↓, ERK1/2 signaling
Hdac9	209	1557	Histone deacetylase 9	transcription factor binding	transcript.regulation, inflamm. IR, B cell activation
Hmox1	9634	49122	Heme oxygenase 1	oxidation-reduction	hypoxia resp., intrinsic apoptotic sign., IκB-NFκB sign.
Ifngr2	1310	4129	Ifngr2 protein	IL10 receptor activity	IR regulation, IFNγ signaling regulation
Igf1	194	4505	Insulin-like growth factor 1	receptor binding & signaling	IGF1R signaling, hemostasis, development
Igsf23	332	1321	Igsf23 protein	IR gene family	
Il1r1	301	1675	Interleukin-1 receptor type 1	IL-1 signaling	IR regulation
Il6st	3286	10126	Interleukin6 receptor subunit beta	signaling	IL6, oncostatinM, NOTCH, IL27 signaling, T cell prolif.
Inadl	1109	4672	InaD-like protein	protein binding	multimeric complex organization, TJ, signaling
Inhba	814	2779	Inhibin beta A chain	cytokine activ, receptor binding, cell cycle	extrinsic apoptosis, SMAD phosphoryl., hair follicle ↓
Irak3	1023	3230	Interleukin-1 receptor-associated kinase 3	kinase activity, neg.regul.IL6, IL12, TNF product.,.	innate IR↓, Myd88/TLR, NFκB sign. ↓, IL1 signaling
Jag1	289	1143	Protein jagged-1	regulation of proliferation	NOTCH signaling
Kcna2	544	2961	Potassium voltage-gated channel subfamily A 2	ion channel activity	ion transmembrane transport
Lgmn	3430	10461	Legumain	peptidase, proteolysis	TLR cascade, MHCII antigen processing
Lhfpl2	1198	4169	Lipoma HMGIC fusion partner-like 2 protein	tetraspanin family, platelet degranulation	hemostasis
Lpl	1797	11860	Lipoprotein lipase	lipid metabolism, chemokine secretion	lipid & lipoprotein metabolism
Lrp1	2537	10001	Prolow-density lipoprotein receptor-related protein 1	endocytosis, transcytosis	actin organiz., GPCR signaling, lysosomal catabolism
Lrpap1	5607	18250	Alpha-2-macroglobulin receptor-assoc. protein	lipase binding	regulation endocytosis & cell death
Lrrc25	505	1584	Leucine-ricj repeat-cont. protein 25	binding, monocytes and plamacytoid dendtritc cells	
Maf	902	2842	Transcription factor Maf	transcription regulation	cell development, cytokine production
Marcks	726	2765	Myristoylated alanine-rich C-kinase	actin filament assembly	energy metabolisms
Marco	66	1218	Macrophage receptor Marco	endocytosis, apoptotic clearance	GPCR signaling, vesicle-mediated transport
Mfge8	2382	22112	Lactadherin	adhesion, phagocytosis	angiogenesis, phagocytosis, apoptotic cell clearance
Mmp12	33388	179224	Matrix metalloproteinase 12	binding, proteolysis, virus IR regulation	ECM organization, regulation IFNα/ß signaling
Mmp13	2876	32434	Matrix metalloproteinase 13	proteolysis	ECM disassembly
Mmp14	982	3534	Matrix metalloproteinase 14	peptidase	regulation cell migration, proteolysis, ECM organization
Mmp9	1637	13706	Matrix metalloproteinase 9	proteolysis, leukocyte migration,collagen metabolism	axon guidance, EPH-Ephrin signaling
Mrc1	930	5855	Macrophage mannose receptor 1	endocytosis, LPS response	IL4 & IFNγ response
Msr1	3838	16063	Macrophage scavenger receptor types i and II	endocytosis, phagocytosis, cholesterol transport	Ligand uptake, vesicle-mediated transport

Table S7A cont.

Synonym	AA SC	Exo-treated	Protein name	function	biological processes
Nrp2	2457	8104	Neuropilin	protein binding, migration, chemotaxis	LIF response, VEGFR signaling
Pacsin1	563	2113	PKC and casein kinase substrate in neurons protein1	phospholipid & cytoskeleton binding	endocytosis, actin filament organization
Pcdh7	325	1321	Prootein Pcdh7	protein binding	platelet activation & signaling
Pdlim4	156	1026	PDZ and LIM domain protein 4	actin cytoskeleton organization	bone marrow development
Pdpn	1397	6282	Podoplanin	binding	cell-cell adhesion, morphogenesis, platelet activation
Pid1	295	2137	PTB-containing, cubilin and LRP1-interacting protein	response to IL6 & TNF	regulation of ROS response
Plau	1864	6029	Urokinase-type plasminogen activator	peptidase	regulation proliferation, migration, hemostasis
Pltp	650	2297	Phospholipid transfer protein	lipid transporter	lipid metabolism & transport
Pmepa1	746	2509	Protein TMEPAI	R-SMAD binding	SMAD & TGFßR signaling ↓
Pou2f2	834	2612	POU domain, class 2, transcription factor 2	regulation of transcription	B cell maturation
Ppap2b	536	1947	Phospholipid phosphatase 3	cell-cell adhesion, regul.phosphorylation	lipid & lipoprotein metabolism, Wnt signaling
Pros1	1325	4996	Vitamin K-dependent protein S	phagocytosis, negative regulation coagulation	clotting cascade
Ptges	330	1688	Prostagalandin E synthase	lipid metabolism	prostaglandin synthesis
Qsox1	5343	21041	Sulfhydryl oxidase 1	oxidation-reduction	hemostasis
Rgl1	987	3411	Ral guanine nucleotide dissociation stimulator-like 1	GTPase signal transduction	regulation of GTPase activity & lipid metabolism
Rin2	462	1406	Ras and Rab interactor 2	GTPase activator,	endocytosis, signaling
Rragd	208	1273	Ras-related GTP-binding protein D	GTPase	PI3K & TOR signaling
Rxra	657	2037	Retinoic acid receptor RXR-alpha	regulation of transcription	impact on organ development
Saa3	2181	13528	Serum amyloid A-3 protein	chemotaxis, response to IL-1	TLR signaling
Sash1	434	1884	SAM and SH3 domain containing protein 1	protein complex scaffold activity	LPS & JUN & NFκB & p38MAPK signaling
Sbf2	473	1538	Protein Sbf2	phosphatase regulator	membrane organization, myelination
Sdc4	1157	5059	Syndecan 4	fibronectin binding, thrombospondin receptor activity	ECM organiz., focal adhesion, Exo assembly & secret.
Serpinb8	241	1042	Serpin B8	peptidase inhibitor	cell-cell adhesion, platelet activation
Siglec1	184	1303	Sialoadhesin	endocytosis, extrinsic apoptosis signaling	T cell apoptotic process
Slc15a3	1830	5825	Solute carroer family 15 member 3	peptide, protein transport	transmembrane transport
Slc6a8	370	1314	Sodium- nd chloride-dependent creatine transporter 1	ion transport	amino acid metabolism
Slc7a2	6214	29655	Cationic amino acid transporter 2	AA transport, NO production, Mf activation	transmembrane transport, inflammatory IR regulation
Slfn5	2137	7068	Schlafen family member 5	ATP binding	cell differentiation
Snx29	272	1148	Sortin nexin-29	phosphatidylinositol binding	
Spib	1227	4146	Transcription factor Spi-B	transcription	Mϕ differentiation
Spsb1	269	1736	SPRY domain-containing SOCS box	ubiquitin transferase	MHCI antigen process./present., adaptive IS response
Stab1	387	1270	Stabilin-1	scavenger receptor	inflammatory IR, vesicle-mediated transport
Stac2	323	1025	SH3 and cystein-rich domain-containing protein2	ion binding, intracellular signaling	ion channel inactivation
Tgfbi	2818	12397	Transforming growth factor-beta-induced protein ig-h3	adhesion	ECM organization, angiogenesis
Timp2	638	2240	Metalloproteinase inhibitor 2	cytokine response, proteolysis ↓	ECM organization,regul. Ras, cAMP, MAPK signaling
Tlr8	294	1240	Toll-like recptor 8	IL1ß, IL6, IL8, IFNα/ß regulation/secretion, inflamm.IR	MyD88-TLR, IκB-NFκB, sign., MAPK, TLR7/8,9 activ.
Tmie	278	1580	Transmembrane inner ear expressed proteii		sensory hair development inner ear
Tnfrsf4	889	2817	Tumor necrosis factor receptor superfamily member 4	regulation IR & AICD	cytokine signaling, TNFR2-NFκB signaling
Tnfsf8	870	3307	Tumor necrosis factor ligand superfamily member 8	CD8 T cell differentiation, response to bacterium	IR
Trat1	238	2001	T-cell receptor-associated transmembrane adaptor 1	calcium-mediated signaling	receptor recycling↓, T cell receptor signaling
Trem2	602	3204	Triggering receptor expressed on myeloid cells 2	transmembrane signaling receptor,	IR (lymph.-nonlymph.), CCR7, ERK1/2, CD40 signaling
Vcan	1087	5151	Versican core protein	adhesion	GAG synthesis, ECM organization
Wfdc17	1545	5877	Activated macrophage/microglia WAP domain protein	endopeptidase inhibitor	.

Table S7B

Reduced mRNA recovery in spleen cells of MDSC-Exo treated AA mice

Synonym	AA SC	Exo-treated	Protein name	function	biological processes
Adamts14	2537	356	Adamts14	peptidase	ECM organization
Add2	2158	44	Beta-adducin	actin & protein kinase binding	transmembrane transport, leukocyte migration
Alox12	3067	445	Arachidonate 12-lipoxygenase 12S	oxidoreductase activity	lipid & lipoprotein metabolism
Angpt1	1447	217	Angiopoietin-1	angiogenesis	multiple signaling pathways
Aqp1	4415	183	Aquaporin-1	transporter	transmembrane transport
Arsb	5461	969	Arylsulfatase B	hydrolase	autophagy
Atp1b2	3492	106	Sodium/potassium-transport ATPase subunit ß2	ATPase activity	ion transport
B4galnt2	2486	298	Beta-1,4 N-acetylgalactosaminyltransferase 2	glycosylation	negative regulation adhesion
Bzrap1	1884	295	Peripheral-type benzodiazepine rec.-assoc. protein1	protein binding	synapse formation
Camp	1519	43	Cathelicidin antimicrob. Peptide	antimicrobial peptide	innate IR, chemotaxis
Capn5	3836	702	Calpain 5	protease inhibition	proteolysis
Car1	9846	404	Carbonic anhydrase 1	carbon metabolism	O2/CO2 exchange (erythrocytes)
Car2	10370	2065	Carbonic anhydrase 2	carbon metabolism	chloride transport
Casp12	2002	204	Caspase 12	protease	intrinsic apoptosis signaling
Ccnb2	3305	620	G2/mitotic-specific cyclin-B2	cyclin-dependent serine/threonine kinase activity	cell cycle, T cell homeostasis
Cd160	1718	240	CD160 antigen	MHCI receptor activity	IR (bacterium)
Cd177	3385	99	CD177	fibrin clot formation	clotting cascade
Cd244	3150	515	NK receptor 2B4	NK & mDC activation	hemostasis
Cd34	4550	739	Hematopoieitc progenitor cell antigen CD34	binding	hemopoiesis, TGFß&IL10 production, leukoc. migr.
Ces2g	2691	327	Carboxylic ester hydrolase	oxidation	
Chil1	1095	193	Chitinase-3-like protein-1	glycosidase	Inflamm. IR, apoptosis, PKB & NFκB&ERK1/2 activ.
Clca3a1	1379	226	Calcium-sens. chloride conductane protein1	peptidase, ion channel	ion transport
Clec12a	1465	260	C-type lectin domain fam.12memberA	signaling	immune response regulation
Clnk	1719	321	Cytokine-dep. hematopoietic cell linker	transmembrane receptor	tyrosine kinase signaling, IR
Clstn3	1407	185	Calsystenin-3	ion binding	synapse assembly and transmission
Cpa3	43212	6709	Mast cell carboxypeptidase A	proteolysis	peptide hormone metabolism
Ctla2a	13192	2223	Protein CTLA-2a	peptidase	regulation Treg differentiation
Ctla2b	1894	221	CTLA-2b	peptidase inhibitor	Treg conversion
Dixdc1	1248	205	Dixin	enzyme modulator, signaling	microtubule organization, JNK cascade
Dsc2	2697	57	Desmocollin-2	ion binding	cell adhesion
Elane	14228	162	Neutrophil elastase	inflammatory response, phagocytosis, proteolysis	ECM organization & degradation
Epx	5245	186	Eosinophil peroxidase	defense to nematode	oxidative stress response
Ermap	1811	49	Erythroid membr.-assoc. protein	erythroid membrane receptor	
Esco2	1810	336	N-acetyltransferase	DNA replication	cell cycle
Fads2	1463	136	Fatty acid desaturase 2	oxidorectuctase	lipid & lipoprotein metabolism
Fam64a	1393	278	Protein FAM64A	controls metaphase to anaphase transition	cell cycle
Fcrl6	3671	45	Fc receptor-like protein6	MHCII ligand	CTL & NK receptor, upregulated in chronic imm.stim.
Fdps	1967	289	Farnesyl pyrophosphate synthase	acyltransferase	lipid & lipoprotein metabolism
Gfi1b	1565	251	Zinc finger protein Gfi-1b	regul.transcription	hemopoiesis
Gp1ba	2834	520	Platelet glycoprotein Ib-α	coagulation	clotting cascade
Gp5	1396	253	Platelet glycoprotein V	collagen binding	clotting cascade
Gp9	1090	126	Platelet glycoprotein IX	adhesion	clotting cascade

Table S7B cont.

Synonym	AA SC	Exo-treated	Protein name	function	biological processes
Gucy1a3	1838	321	Guanylate cyclase soluble subunit α3	cGMP biosynthesis	hemostasis
Gucy1b3	1844	341	Guanylate cyclase soluble subunit β1	cGMP biosynthesis, Hsp90 binding	NO-mediated signaling
Gzma	91251	5036	Granzyme A	peptidase	proteolysis, cytolysis
Gzmb	149194	12697	Granzyme B	peptidase	cytolysis, T cell cytotoxicity
Gzmc	43167	1693	Granzyme C	peptidase	proteolysis, cytolysis
Gzmd	75163	898	Granzyme D	peptidase	proteolysis, cytolysis
Gzme	28887	345	Granzyme E	peptidase	proteolysis, cytolysis
Gzmf	26081	359	Granzyme F	peptidase	proteolysis, cytolysis
Gzmg	96614	1092	Granzyme G	peptidase	proteolysis
Havcr2	8662	649	Hepatatis A virus receptor 2	adaptive and innate IR	cytokine signaling
Hmgcs1	10861	1759	Hydroxymeth.-CoA synthase	metabolism	lipid & lipoprotein metabolism
Ifitm1	52676	10125	IFN-induced transmembrane protein	response to virus	immune regulation (nonlymphoid-lymphoid)
Ifng	1637	255	Interferon γ	regulation of cytokine production	cytokine signaling in the IS
Itga2b	23212	4422	Integrin α-Ib	leukocyte migration, platelet aggregation	hemostasis, multiple signaling pathways
Kalrn	1263	171	Kalirin	guanyl exchange factor	MAPK & Rho & GPCR signaling, death signaling
Klrg1	1073	57	Killer cell lectin-like receptor G1	innate IR	Immunoregulation (lymphoid-nonlymphoid)
Lipg	4652	620	Endothelial lipase	phospholipase	lipid & lipoprotein metabolism
Ltbp1	1839	206	Latent transforming growth factor ß-binding protein 1	TGFß binding	ECM organization, TFGßR signaling
Ltf	4922	181	Lactotransferrin	protease, ROS/RNS production	regulation cytokine production, innate IR
Ly6c1	6129	768	Lymphocyte antigen 6C1	monocyte marker	inflammatory process orchestration
Ly6c2	9867	1265	Lymphocyte antigen 6C2	monocyte marker	inflammatory process orchestration
Maob	1138	149	Amine oxidase B	oxidation-reduction	biological oxidation
Mcpt8	18692	2308	Mast cell protease	proteolysis	inflammatory respone in the skin
Mfsd2b	3503	511	Major facilitator superfam.dom.-contain.protein2B	enzyme activity	transport
Mns1	1283	140	Meiosis-specific nuclear struct.protein 1	protein binding	cilium organization
Mpl	1661	229	Thrombopoietic receptor	cytokine receptor activity	regulation of hemopoiesis and homeostasis
Mpo	23160	789	Myeloperoxidase	oxidation-reduction process	azurophilic granules, innate IR
Mrvi1	5207	768	Mrvi1	regulation of smooth muscle contraction	cGMP signaling
Ms4a2	7186	1234	High affinity Igepsilon R subunit ß	FcεRI signaling	MAPK & NFκB activation, inflammatory IR
Ms4a3	2383	80	Membr.-spanning 4 domain subfamily A3	CD20L,cell cycle regulation	IS signal transduction, neutrophil degranulation
Myb	5422	809	Transcriptional activator Myb	transcription factor	TH & B & myeloid cell differentiation
Mycn	1669	268	N-myc proto-oncogene protein	transcription	regulation cell death
Myl10	1050	180	Myosin regulatory light chain 10	cytoskeletal protein	regulation focal adhesion, leukocyte migration
Mylk	3936	642	Myosin light chain kinase	actin binding	regul. migration & transport, RhoGTPase signaling
Ngp	31130	394	Neutrophilic granule protein	peptidase inhibition	regulation defense response
Nkg7	17727	2786	Protein NKG7	cytokeletal protein	adhesion, transporter
Nlrp6	5308	912	NACHT,LRR & PYD dom. containing prot.6	transcription cofactor	IR ↓, TLR&MAPK&IκB/NFκB signaling ↓
Nqo1	1689	276	NAD(P)H dehydrogenase 1	regulation of oxidate and apoptotic processes	oxidation-reduction process
Nrgn	1471	206	Neurogranin	synaptic potentiol	intracellular signaling
Pkd2l1	1615	298	Polycystic kidney disease 2-like 1	ion transport	channel activity
Podxl	5715	920	Podocalyxin	protein binding	regulation leukocyte adhesion & migration
Prf1	39787	2235	Perforin	wide pore formation	channel activity, apoptosis, cytolysis
Prg2	3593	335	Bone marrow proteoglycan	component of eosinophil granule	IR to nematode & bacterium

Table S7B cont.

Synonym	AA SC	Exo-treated	Protein name	function	biological processes
Prss34	6940	934	Mast cell protease 11	peptide crosslinking & proteolysis	basophil mediated tissue damage
Prtn3	5128	168	Myeloblastin	protease inhibition	proteolysis, phagocytosis
Rab27b	3767	698	Ras-related protein Rab-27B	GTPase activity	Exo sorting & exocytosis
Rab38	4129	787	Ras-relat. protein Rab-38	GTPase activity	organelle organization & transport
Rhag	1136	110	Ammonium transporter Rh type A	iron homeostasis	transmembrane transport
S100a4	9322	692	Protein S100-A4	actin binding	IκB/NFκB signaling
S100a6	14291	2415	Protein S100-A6	calcium and protein binding	ion transport, plasma membrane tubulation
S100a8	37682	4415	Protein S100-A8	neutrophil chemotaxis, apoptosis	inflammatory response
S100a9	57146	4653	Protein S100-A9	chemotaxis, inflamm.response, apoptosis	TLR cascade
Samd14	1645	304	Sterile alpha motic dom.cont. protein 14	aberrant promoter methylation	
Scin	14700	2765	Adseverin	actin binding	actin nucleation, apoptosis regulation
Sdpr	1753	268	Serum-depriv. response protein	PKC substrate, phosphatidyl binding	plasma membrane tabulation
Serpinb9b	3296	376	Serpinb9b	cytolysis	protection from cell-mediated cytotoxicity
Sh2d5	1812	123	SH2 domain-contain.protein 5	protein binding	
Slc6a4	1510	259	Sodium-depend. serotonin transporter	transporter	synapse transmission
Spns2	2092	317	Protein spinster homolog 2	lipid transport	B & T cell homeostasis, lymphocyte migration
Spta1	1842	66	Spectrin α chain,erythrocytic-1	actin organization	regul.hemopoiesis, T cell prolif., multiple signaling
Sytl2	3530	462	Synaptogamin-like protein2	membrane traffic regulator	vesicle docking & exocytosis
Tfr2	1478	240	Transferrin receptor protein2	regulation iron ion homeostasis	regulation of endocytosis
Timp3	6914	995	Metalloproteinase inhibitor 3	protease inhibition	regulation proteolysis, TRAIL signaling pathway
Tjp1	1380	211	TJ protein ZO1	GAP junction formation	membrane & vesicle-mediated transport
Tmem119	4546	467	Transmembrane protein 119	CD11b+ cells in microglia	osteoblast differentiation, spermatogenesis
Tmem40	1096	104	Transmembrane protein 40		biological process
Treml1	1496	255	Trem-like transcript 1 protein	Ig receptor	lymphoid-nonlymph. interaction, neg.regul. signaling
Tubb1	1651	154	Tubulin β1 chain	GTPase	organelle biog., membr.traff., vesicle-med. transport
Vash1	3055	345	Vasohibin1	protein binding	angiogenesis regulation, cellular sensecence
Vcam1	1055	39	Vascular cell adhesion protein1	integrin binding	IR regulation (lymphoid-nonlymphoid)
Vwf	13563	2121	vonWillebrand factor	blood coagulation	clotting cascade, beta3 signaling
Zbtb32	5045	816	Zinc finger and BTB domain-cont. protein 32	transcription corepressor	osteoblast different.,hematopoietic prog. proliferation

[a] AA: amino acid, AICD: activation-induced cell death, DC: dendritic cells, ECM: extracellular matrix, IS: immune system, IR: immune response, TJ: tight junction, ↓: reduced recovery / activity, ↑: pronounced recovery / activity, red: engagement in hair follicle organization

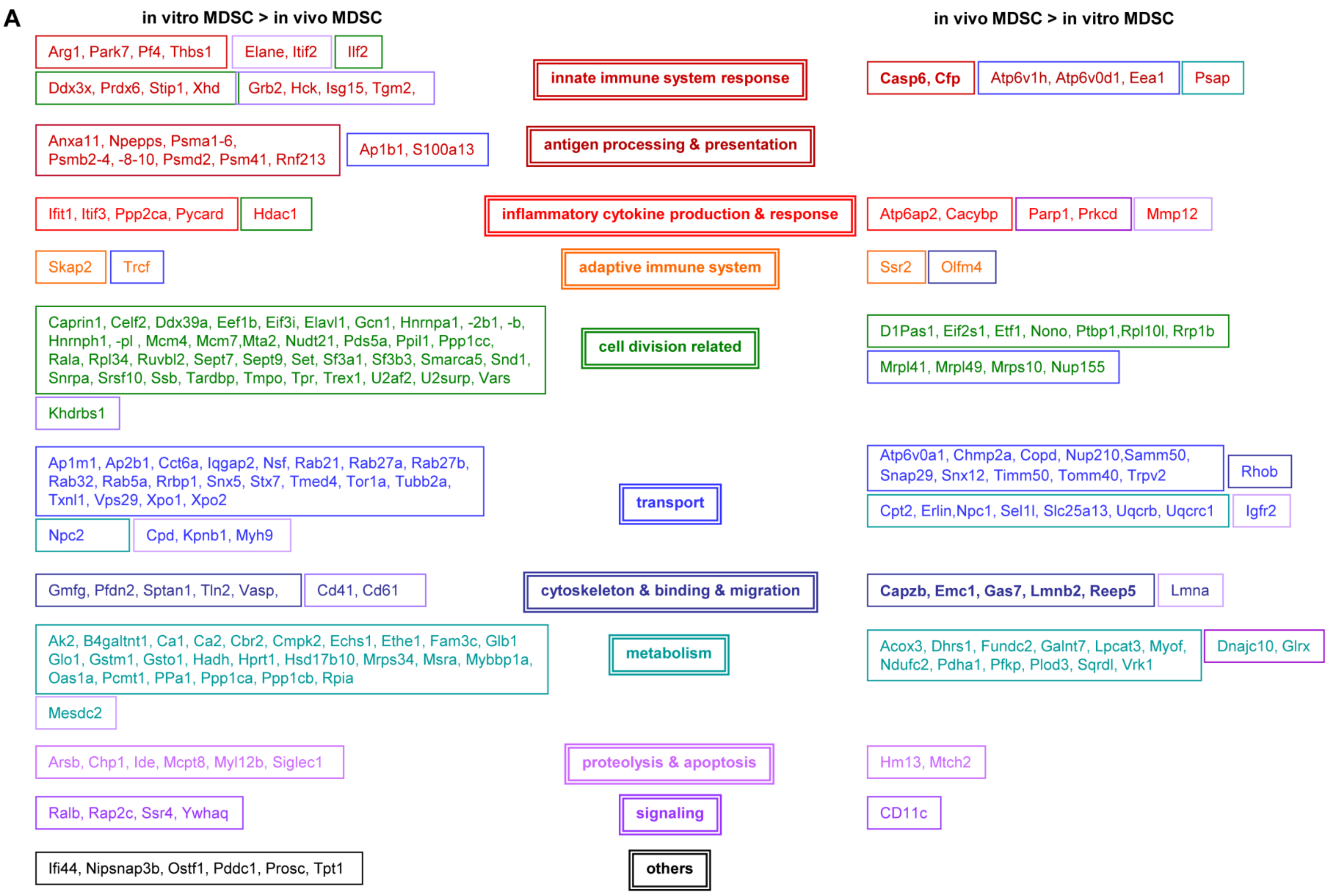
A
in vitro MDSC > in vivo MDSC
in vivo MDSC > in vitro MDSC
Arg1, Park7, Pf4, Thbs1
Elane, Itif2
Ilf2
Ddx3x, Prdx6, Stip1, Xhd
Grb2, Hck, Isg15, Tgm2,
innate immune system response
Casp6, Cfp
Atp6v1h, Atp6v0d1, Eea1
Psap
Anxa11, Npepps, Psma1-6, Psmb2-4, -8-10, Psmd2, Psm41, Rnf213
Ap1b1, S100a13
antigen processing & presentation
Ifit1, Itif3, Ppp2ca, Pycard
Hdac1
inflammatory cytokine production & response
Atp6ap2, Cacybp
Parp1, Prkcd
Mmp12
Skap2
Trcf
adaptive immune system
Ssr2
Olfm4
Caprin1, Celf2, Ddx39a, Eef1b, Eif3i, Elavl1, Gcn1, Hnrnpa1, -2b1, -b, Hnrnph1, -pl , Mcm4, Mcm7,Mta2, Nudt21, Pds5a, Ppil1, Ppp1cc, Rala, Rpl34, Ruvbl2, Sept7, Sept9, Set, Sf3a1, Sf3b3, Smarca5, Snd1, Snrpa, Srsf10, Ssb, Tardbp, Tmpo, Tpr, Trex1, U2af2, U2surp, Vars
Khdrbs1
cell division related
D1Pas1, Eif2s1, Etf1, Nono, Ptbp1,Rpl10l, Rrp1b
Mrpl41, Mrpl49, Mrps10, Nup155
Ap1m1, Ap2b1, Cct6a, Iqgap2, Nsf, Rab21, Rab27a, Rab27b, Rab32, Rab5a, Rrbp1, Snx5, Stx7, Tmed4, Tor1a, Tubb2a, Txnl1, Vps29, Xpo1, Xpo2
Npc2
Cpd, Kpnb1, Myh9
transport
Atp6v0a1, Chmp2a, Copd, Nup210,Samm50, Snap29, Snx12, Timm50, Tomm40, Trpv2
Rhob
Cpt2, Erlin,Npc1, Sel1l, Slc25a13, Uqcrb, Uqcrc1
Igfr2
Gmfg, Pfdn2, Sptan1, Tln2, Vasp,
Cd41, Cd61
cytoskeleton & binding & migration
Capzb, Emc1, Gas7, Lmnb2, Reep5
Lmna
Ak2, B4galtnt1, Ca1, Ca2, Cbr2, Cmpk2, Echs1, Ethe1, Fam3c, Glb1 Glo1, Gstm1, Gsto1, Hadh, Hprt1, Hsd17b10, Mrps34, Msra, Mybbp1a, Oas1a, Pcmt1, PPa1, Ppp1ca, Ppp1cb, Rpia
Mesdc2
metabolism
Acox3, Dhrs1, Fundc2, Galnt7, Lpcat3, Myof, Ndufc2, Pdha1, Pfkp, Plod3, Sqrdl, Vrk1
Dnajc10, Glrx
Arsb, Chp1, Ide, Mcpt8, Myl12b, Siglec1
proteolysis & apoptosis
Hm13, Mtch2
Ralb, Rap2c, Ssr4, Ywhaq
signaling
CD11c
Ifi44, Nipsnap3b, Ostf1, Pddc1, Prosc, Tpt1
others

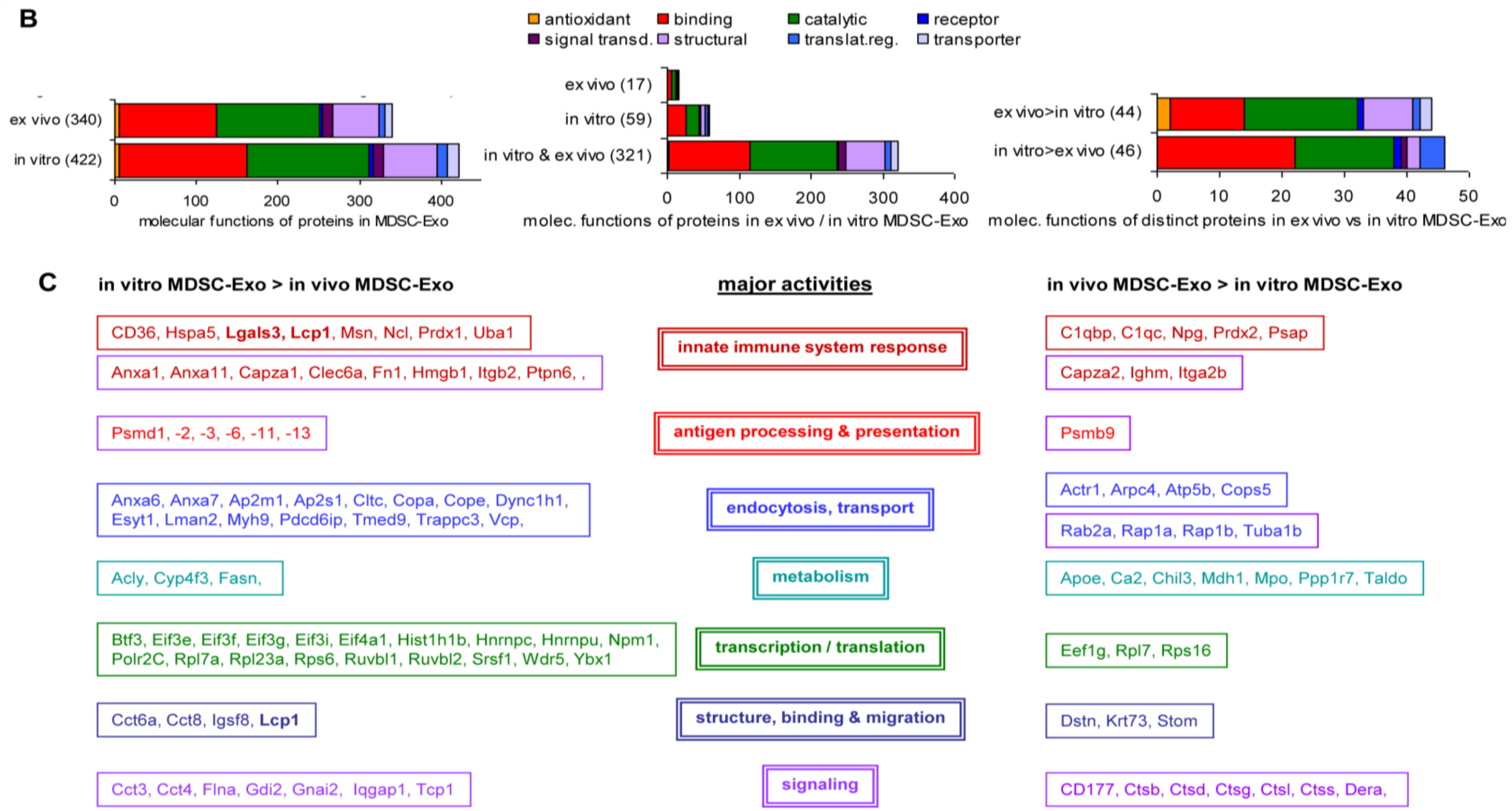

Figure S1 **Proteome analysis of *ex vivo* and *in vitro* generated MDCS and MDSC-exosomes.** (A) MDSC were either isolated from SC by magnetic bead sorting for CD11b+Gr1+ cells or were generated *in vitro* from BMC cultured in the presence of GMCSF, IL6 and PGE2. After SDS-Page and digestion, proteins were identified by mass spectrometry. Proteins preferentially recovered in *ex vivo* or *in vitro* generated MDSC were clustered using Reactome according to their engagement in immune response, cell cycle, transport and cytoskeleton organization, proteolysis / apoptosis and signaling. Proteins engaged in two clusters are identified by a distinct frame color. (B,C) Exo were collected from MDSC as described above after 2d culture in medium with 3% Exo-depleted FCS. After purification, SDS-Page and digestion, proteins were identified by mass spectrometry. (B) Comparison of abundantly recovered proteins in MDSC-Exo derived from *ex vivo* versus *in vitro* generated MDSC including proteins that are selectively recovered from the two distinct MDSC preparations and (C) MDSC-Exo preferentially recovered from *ex vivo* or *in vitro* generated MDSC were clustered according to major functional activities as described for MDSC.

A proteome analysis confirmed no significant differences in *ex vivo* versus *in vitro* generated MDSC according to immune system-related functions. The abundance of distinctly in *in vitro* generated MDSC relying mostly on pronounced cell division and associated metabolic processes due to the culture condition. This also accounts for the differential recovery in Exo from *ex vivo* or *in vitro* MDSC, where the significant increase in proteins engaged in cell division and metabolic processes is also reflected in Exo from culture-derived MDSC.

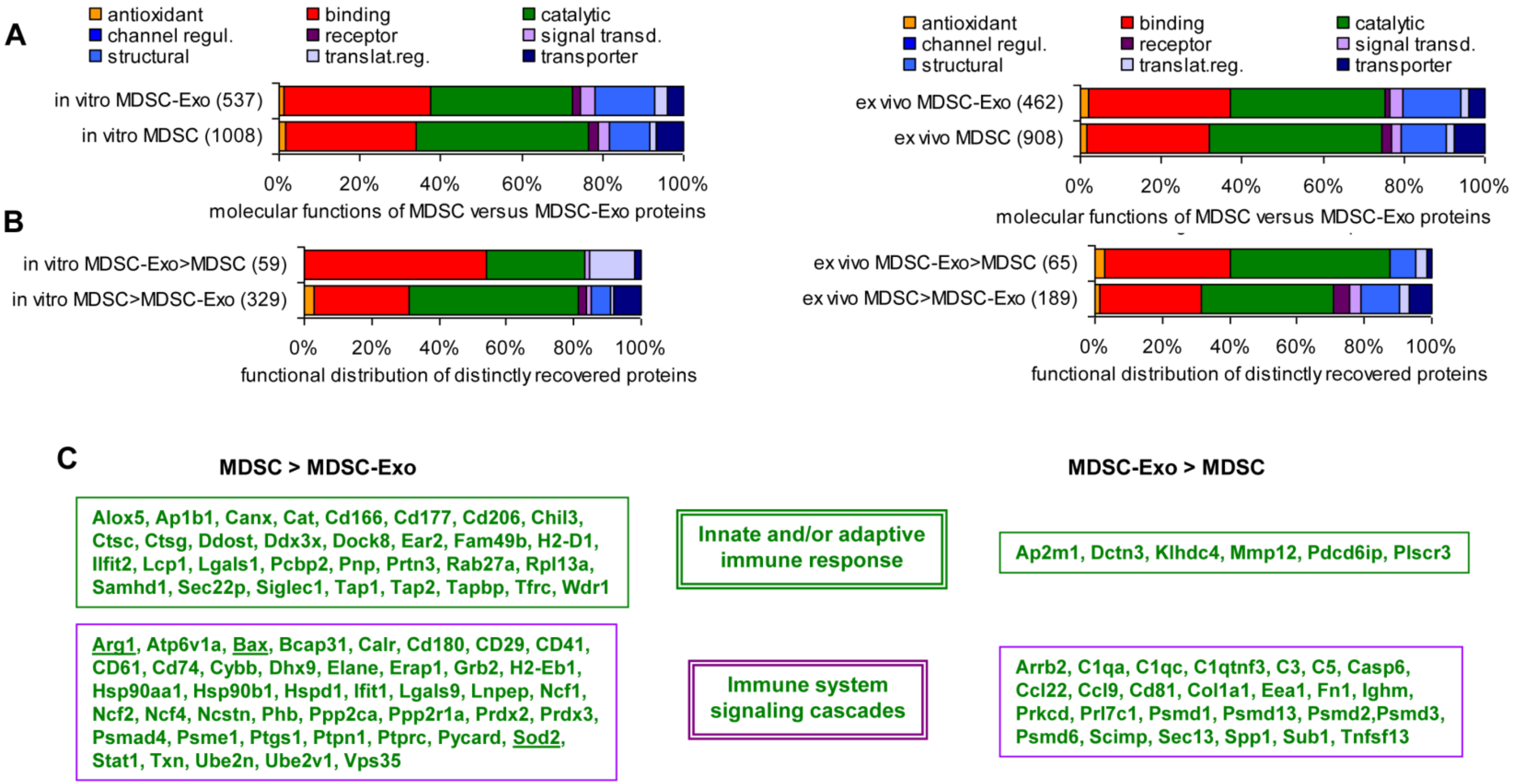

Figure S2 **Proteome analysis comparing *ex vivo* and *in vitro* generated MDCS with MDSC-exosomes.** MDSC were generated *in vitro* from BMC. After maturation, MDCS were cultured for 2d in medium with 3% FCS that was Exo-depleted. MDSC and MDSC-Exo proteins were identified by mass spectrometry. (A) Panther Gene Analysis of molecular functions of *in vitro* and *ex vivo* MDSC versus MDSC-Exo; (B) Panther Gene Analysis of molecular functions of proteins that are distinctly recovered in *in vitro* and *ex vivo* MDSC versus MDSC-Exo. (C) Reactome analysis of distinctly recovered proteins in BMC-derived MDSC versus MDSC-Exo, which are engaged in immune response regulation including activation of signaling cascades.

Three proteins engaged in MDSC activity, Arg1, Sod2 and Bax were recovered at a lower level in MDSC-Exo. On the other hand, tetraspanins, C' and proteasome subunits were more abundant in MDSC-Exo than MDSC. Hypothesizing that pro and contra of function-relevant proteins in MDSC versus MDCS-Exo could possibly be balanced, we proceeded comparing their effectivity in immune response regulation.

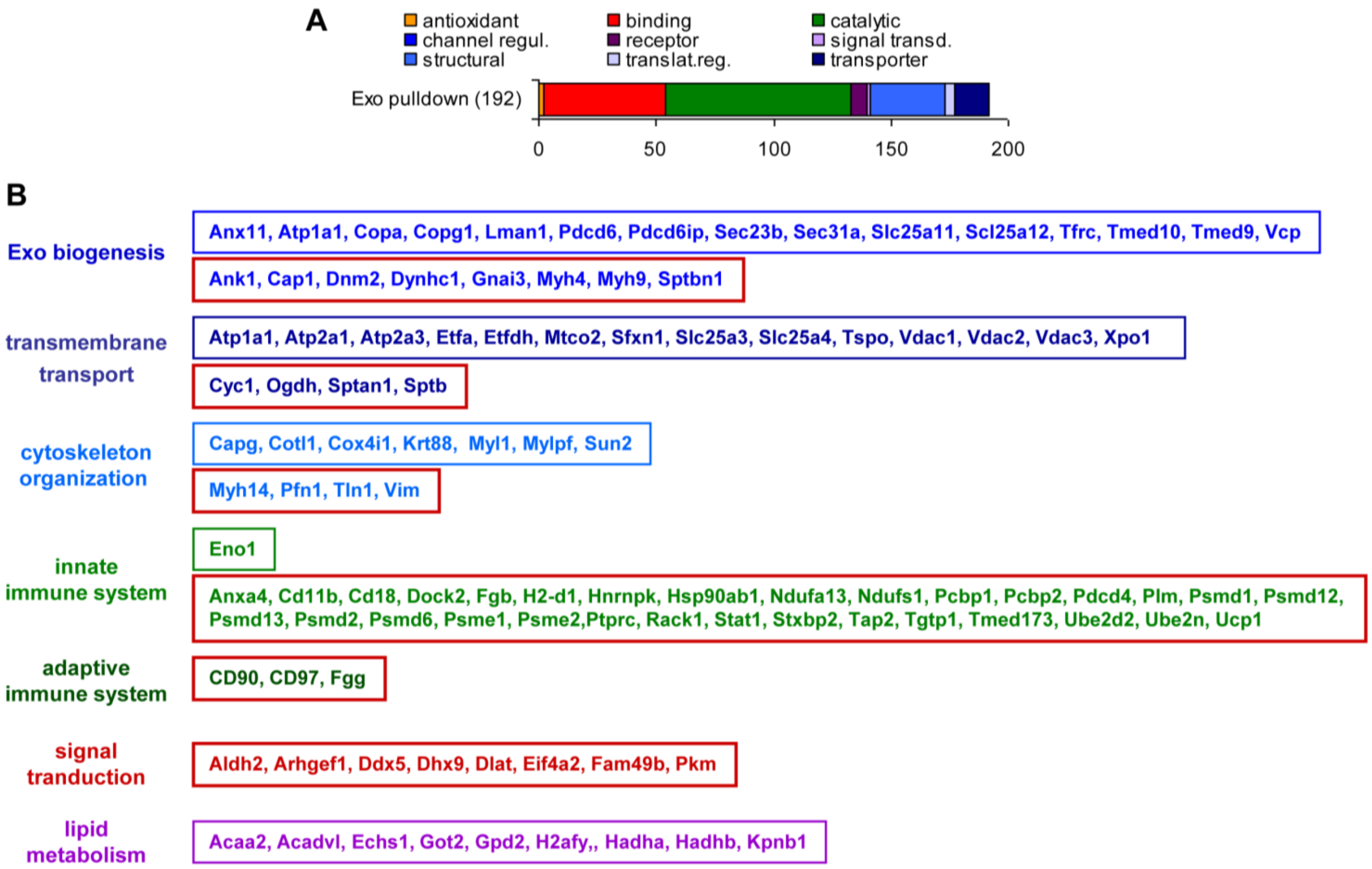

Figure S3 **MDSC-exosome membrane attached proteins**. A lymphocyte membrane preparation was coupled to Sepharose. MDSC-Exo were lysed (mild conditions, Lubrol) and were passed over the lymphocyte-membrane Sepharose column. After washing, Exo lysates were eluted, dialyzed, concentrated and subjected to SDS-PAGE for proteome analysis. (A) Pulled-down MDSC-Exo proteins were clustered according to molecular functions (Panther Gene Analysis) and (B) using Reactome according to proteins engaged in Exo biogenesis, transport, cytoskeleton organization, engagement in innate and adaptive immune responses as well as lipid metabolism. Proteins engaged in signal transduction are framed in red.

The pulldown of MDSC-Exo indicated that invaginated membrane domains remain intact during Exo biogenesis including attached cytoskeletal and signaling molecules. The latter may well contribute to immune target cell modulation.

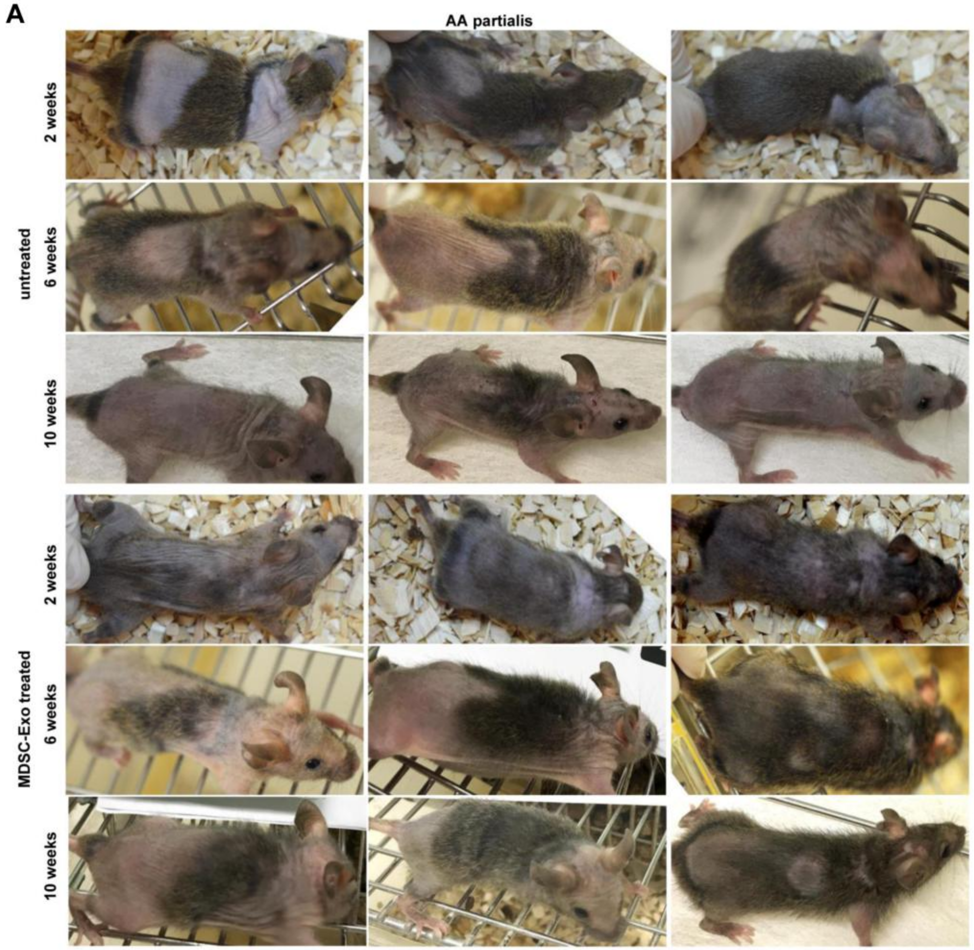
A
AA partialis
untreated
2 weeks
6 weeks
10 weeks
MDSC-Exo treated
2 weeks
6 weeks
10 weeks

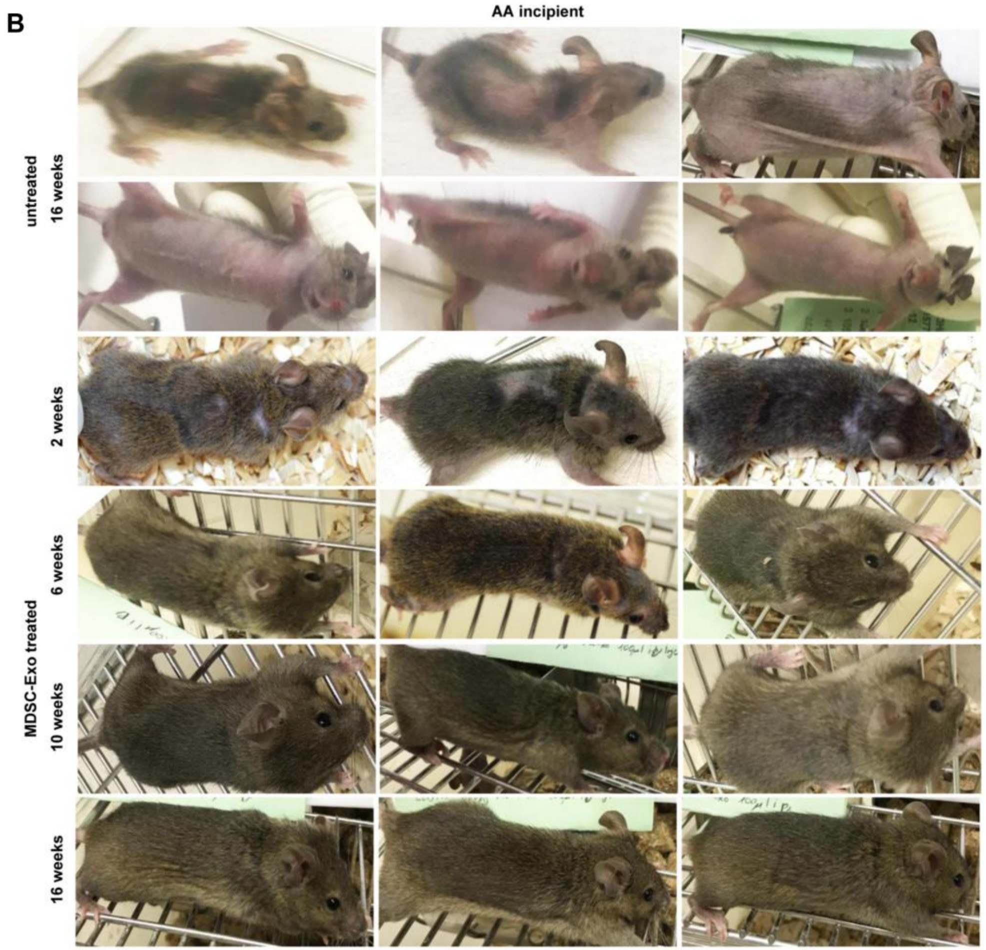

Figure S4 **Examples of hair regrowth in MDSC exosome-treated mice with AA partialis and incipient**. (A) Hair loss progression in untreated mice with AA partialis and partial hair growth recovery in MDSC-Exo treated mice is demonstrated after 2wk, 6wk and 10wk (3 mice / group); (B) Hair loss progression in untreated mice with AA incipient 16wk after initiating MDSC-Exo application; hair growth in MDSC-Exo treated mice is shown after 2wk, 6wk, 10wk and 16wk.

MDSC-Exo treatment is curative when started at a very early stage of hair loss. At an intermediate state it prevents progression. Partial hair regrowth proceeds from remaining non-affected areas.
